# Confronting the Challenge of Modeling Cloud and Precipitation Microphysics

**DOI:** 10.1029/2019MS001689

**Published:** 2020-07-31

**Authors:** Hugh Morrison, Marcus van Lier‐Walqui, Ann M. Fridlind, Wojciech W. Grabowski, Jerry Y. Harrington, Corinna Hoose, Alexei Korolev, Matthew R. Kumjian, Jason A. Milbrandt, Hanna Pawlowska, Derek J. Posselt, Olivier P. Prat, Karly J. Reimel, Shin‐Ichiro Shima, Bastiaan van Diedenhoven, Lulin Xue

**Affiliations:** ^1^ National Center for Atmospheric Research Boulder CO USA; ^2^ NASA Goddard Institute for Space Studies and Center for Climate Systems Research Columbia University New York NY USA; ^3^ NASA Goddard Institute for Space Studies New York NY USA; ^4^ Department of Meteorology and Atmospheric Science The Pennsylvania State University University Park PA USA; ^5^ Institute of Meteorology and Climate Research Karlsruhe Institute of Technology Karlsruhe Germany; ^6^ Observation Based Research Section Environment and Climate Change Canada Toronto Ontario Canada; ^7^ Atmospheric Numerical Prediction Research Environment and Climate Change Canada Dorval Quebec Canada; ^8^ Institute of Geophysics, Faculty of Physics University of Warsaw Warsaw Poland; ^9^ Jet Propulsion Laboratory California Institute of Technology Pasadena CA USA; ^10^ North Carolina Institute for Climate Studies North Carolina State University Asheville NC USA; ^11^ University of Hyogo and RIKEN Center for Computational Science Kobe Japan

**Keywords:** microphysics, clouds, modeling

## Abstract

In the atmosphere, *microphysics* refers to the microscale processes that affect cloud and precipitation particles and is a key linkage among the various components of Earth's atmospheric water and energy cycles. The representation of microphysical processes in models continues to pose a major challenge leading to uncertainty in numerical weather forecasts and climate simulations. In this paper, the problem of treating microphysics in models is divided into two parts: (i) how to represent the population of cloud and precipitation particles, given the impossibility of simulating all particles individually within a cloud, and (ii) uncertainties in the microphysical process rates owing to fundamental gaps in knowledge of cloud physics. The recently developed Lagrangian particle‐based method is advocated as a way to address several conceptual and practical challenges of representing particle populations using traditional bulk and bin microphysics parameterization schemes. For addressing critical gaps in cloud physics knowledge, sustained investment for observational advances from laboratory experiments, new probe development, and next‐generation instruments in space is needed. Greater emphasis on laboratory work, which has apparently declined over the past several decades relative to other areas of cloud physics research, is argued to be an essential ingredient for improving process‐level understanding. More systematic use of natural cloud and precipitation observations to constrain microphysics schemes is also advocated. Because it is generally difficult to quantify individual microphysical process rates from these observations directly, this presents an inverse problem that can be viewed from the standpoint of Bayesian statistics. Following this idea, a probabilistic framework is proposed that combines elements from statistical and physical modeling. Besides providing rigorous constraint of schemes, there is an added benefit of quantifying uncertainty systematically. Finally, a broader hierarchical approach is proposed to accelerate improvements in microphysics schemes, leveraging the advances described in this paper related to process modeling (using Lagrangian particle‐based schemes), laboratory experimentation, cloud and precipitation observations, and statistical methods.

## Key Points

- Microphysics is an important component of weather and climate models, but its representation in current models is highly uncertain
- Two critical challenges are identified: representing cloud and precipitation particle populations and knowledge gaps in cloud physics
- A possible blueprint for addressing these challenges is proposed to accelerate progress in improving microphysics schemes

**Figure 1:**
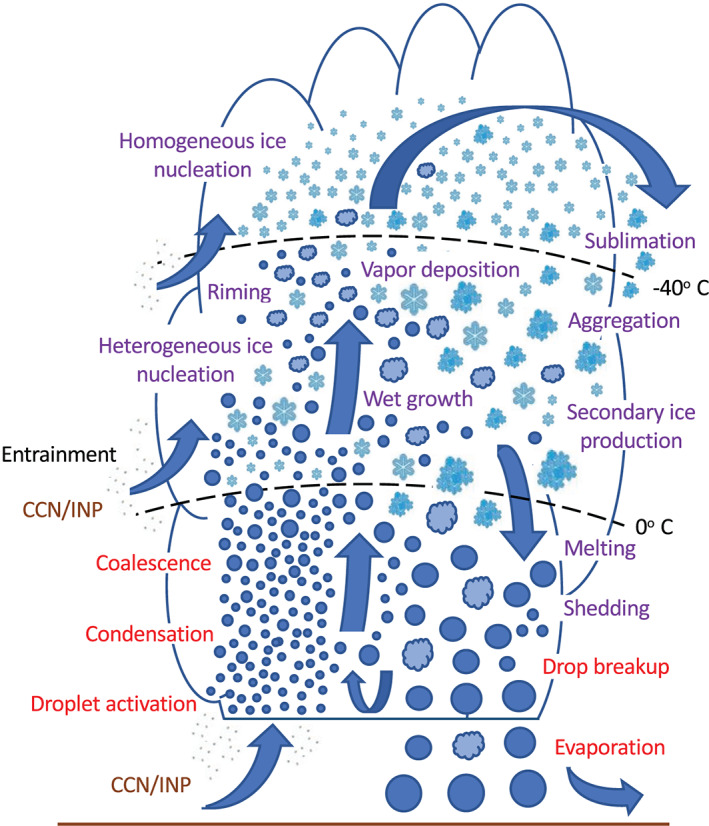
Schematic illustration of microphysical processes within a typical cumulonimbus cloud, highlighting the complexity of microphysics in the atmosphere. Specific microphysical processes are listed in red (involving only liquid drops) and purple (involving ice particles only or both liquid and ice). Cloud *droplet activation* occurs on aerosol particles serving as cloud condensation nuclei (CCN) in supersaturation conditions; cloud droplets then grow by *condensation*. Further growth by collision‐coalescence produces raindrops. Above the 0°C level, there is *heterogeneous ice nucleation* on aerosols serving as ice nucleating particles (INP). Ice particles grow by *vapor deposition* and *riming* (i.e., accretion and freezing of supercooled drops). If riming is especially heavy, not all of the collected liquid water freezes onto the ice particles and some is shed, representing *wet growth*. Above approximately the −40°C level, *homogeneous ice nucleation* can generate additional ice particles. *Sublimation* of ice particles detrained from the cloud occurs in subsaturated conditions. Ice crystals can grow by aggregation when they collide and stick together. *Secondary ice production*, not associated with heterogeneous or homogeneous ice nucleation, can generate more ice particles. Below the 0°C level, ice particle *melting* generates raindrops, and *shedding* of meltwater occurs for some ice particles. Raindrop collision‐coalescence produces larger drops, while raindrop *breakup* produces smaller ones. Below cloud base, *evaporation* of falling raindrops occurs in subsaturated air.

In the atmosphere, microphysics refers to the small‐scale (from sub‐micron to cm) processes driving the formation and evolution of cloud and precipitation particles. These processes include nucleation, condensation growth by vapor diffusion, collision and coalescence, freezing, and melting, among others (Figure 1). Microphysics is extremely complicated because of the huge number of particles present in clouds, the wide variety of ice particle shapes, and the complex, nonlinear interactions among specific processes. Microphysics parameterization schemes in atmospheric models attempt to represent the behavior of cloud and precipitation particle populations and their effects on weather and climate. Microphysics schemes strongly influence forecasts of high impact weather events from localized severe convective storms to tropical cyclones and snow storms. Microphysics schemes also have a critical impact on how simulated clouds interact with incoming solar radiation and Earth's outgoing longwave radiation, and thus on simulated climate. For example, a recent paper (Hofer et al., 2019) showed that the phase of cloud particles (liquid vs. ice) had a strong influence on simulations of future Greenland ice sheet melting. One of the key ways in which microphysics affects climate is through the influence of pollution aerosols on the size and number of cloud particles, and this is one of the largest uncertainties in assessments of climate change (IPCC, 2013).

Microphysics schemes face two major challenges: (i) how to represent the population of cloud and precipitation particles, given the impossibility of simulating all particles individually even within a small cloud, and (ii) uncertainties in microphysical process rates owing to critical gaps in cloud physics knowledge. These uncertainties are especially large for ice‐phase processes such as vapor diffusional growth, melting, and aggregation (sticking and collection of ice particles) owing to the complicated and intricate shapes of atmospheric ice particles.

The traditional approach for representing particle populations within a grid volume, extending back to the earliest development of microphysics schemes in the 1950s and 1960s, relies on predicting continuous‐medium, Eulerian cloud and precipitation variables. *Bulk* microphysics schemes predict one or a few variables that describe bulk properties of cloud within a grid volume, such as the cloud mass. *Bin* schemes represent particle distributions explicitly and predict variables such as the cloud mass within a model volume over some size interval of the distribution. Bin schemes have many more predicted variables to evolve the microphysical properties than bulk schemes, providing much more flexibility and degrees of freedom, but are computationally costly. The approach of using continuous‐medium, Eulerian variables in both bulk and bin schemes leads to several conceptual and practical challenges. A much different parameterization approach has gained traction within the past 10 years—the Lagrangian particle‐based method. In Lagrangian particle‐based schemes, the particle population is represented by a discrete sampling of cloud and precipitation particles (called “super‐particles”), each representing some multitude of real particles that follow trajectories in the modeled flow. Besides addressing several practical challenges of bulk and bin schemes, particle‐based schemes have a fundamental conceptual advantage: as the number of super‐particles approaches the number of actual particles, and the model grid resolution decreases to resolve all scales of atmospheric motion and turbulence (down to ~1 mm scale), particle‐based schemes converge to detailed turbulence models that represent all particles individually. In principle, this provides a rigorous path toward numerical convergence for cloud modeling, which is not possible using traditional bulk and bin schemes that fundamentally cannot represent discrete particles moving in a fluid, as occurs in real clouds.

For addressing critical gaps in cloud physics knowledge, which lead to major uncertainties in all models including those using the Lagrangian particle‐based approach, we advocate sustained investment for observational advances from laboratory experiments, new probe development, and next‐generation instruments in space. Because laboratory experimentation provides a direct way to quantify individual microphysical process rates in a controlled setting, they are a critical part of advancing cloud physics knowledge. Nonetheless, there has been an apparent decline in laboratory work over the past several decades relative to other research areas in cloud physics. We advocate increased support for laboratory work to address major gaps in cloud physics knowledge and to provide data for developing physically based parameterizations for models. We also advocate sustained support for new airborne and ground‐based instrument development and next‐generation instruments in space to provide field data needed to evaluate and constrain microphysics schemes in regional and global models.

A major challenge using the wealth of natural cloud and precipitation observations to constrain microphysics schemes is that it is generally very difficult to obtain individual microphysical process rates directly from these observations; essentially, they provide snapshots of cloud and precipitation properties that result from various processes acting over time. This presents an inverse problem: microphysical process rates in schemes can generally be constrained only indirectly by comparing model output with observations. We propose that this inverse problem can be viewed probabilistically through Bayesian statistics. Centered on this idea, we propose a statistical‐physical approach for parameterizing microphysics that uses Bayesian inference to constrain scheme parameters and model structure using cloud and precipitation observations rigorously and systematically. This contrasts with the traditional approach for microphysics scheme development based on a purely “physical” approach combined with heuristics and often ad hoc “tuning” of parameter values. Besides providing rigorous observational constraint, a major advantage of Bayesian methods is that uncertainty is quantified systematically. While such methods have had little use in microphysical modeling, they have been widely incorporated into land surface and hydrological modeling, which face similar challenges to microphysics owing to extreme complexity and poorly understood chemical‐physical‐biological processes. Finally, we propose a broader hierarchical approach to accelerate improvements in microphysics schemes, leveraging the advances described in this paper related to process modeling using Lagrangian particle‐based schemes, laboratory experimentation, cloud and precipitation observations, and statistical methods.

**General references for further reading:**

^*^Hofer, S., A. J. Tedstone, X. Fettweis, and J. L. Bamber (2019), Cloud microphysics and circulation anomalies in future Greenland melt, *Nature Clim. Change*, 9, 523‑528.

Houze, R. A., Jr. (2014), Cloud dynamics, 2nd edition, Elsevier Inc., 431 pp.

^*^Khain, A. P., and Coauthors (2015), Representation of microphysical processes in cloud‐resolving models: Spectral (bin) microphysics versus bulk parameterization, *Rev. Geophys*., 53, 247‑322.

^*^Khain, A. P., and M. Pinsky (2018), *Physical processes in clouds and cloud modeling*, Cambridge University Press, 626 pp.

Kreidenweis, S. M., M. Petters, and U. Lohmann (2019), 100 years of progress in cloud physics, aerosols, and aerosol chemistry, Meteor. Monog. (in press), doi:/10.1175/AMSMONOGRAPHS‐D‐18‐0024.1.

^*^IPCC (2013), *Climate change 2013: The physical science basis. Contribution of Working Group I to the Fifth Assessment Report of the Intergovernmental Panel on Climate Change* [Stocker, T.F., D. Qin, G.‐K. Plattner, M. Tignor, S.K. Allen, J. Boschung, A. Nauels, Y. Xia, V. Bex and P.M. Midgley (eds.)]. Cambridge University Press, Cambridge, United Kingdom and New York, NY, USA, 1535 pp, doi:10.1017/CBO9781107415324.

Tao, W.‐K. and Coauthors (2019), Microphysics in Goddard multi‐scale modeling systems: A review, in *Current trends in the representation of physical processes in weather and climate models*, Springer, 253‐316.

^*^Also referenced in the main text.

## 1 The Problem of Representing Cloud and Precipitation Microphysics in Models

In the atmosphere, *microphysics* refers to the physical and chemical processes occurring at the scale of individual cloud and precipitation particles, or hydrometeors (sub‐micron to several centimeters). Such processes include the nucleation of cloud particles, their diffusional growth from water vapor, collision and coalescence, freezing, melting, and evaporation. These processes determine the characteristics of cloud particle populations and drive the formation of precipitation; these effects have to be accounted for in cloud, weather, and climate models. Changes in thermal energy from water phase changes, for example, from condensation and melting, affect the buoyancy of air parcels and are therefore key drivers of cloud dynamics. Microphysical properties (e.g., shape, size, and phase of particles) are critical to radiative transfer in clouds, which is crucial for climate. The representation of microphysical processes can strongly influence cloud‐climate feedbacks in global climate models (e.g., Bodas‐Salcedo et al., 2019). Cloud‐radiative interaction is also modulated by aerosols via cloud microphysics, which is one of the major uncertainties in anthropogenic climate change (IPCC, 2013).

**Figure 2:**
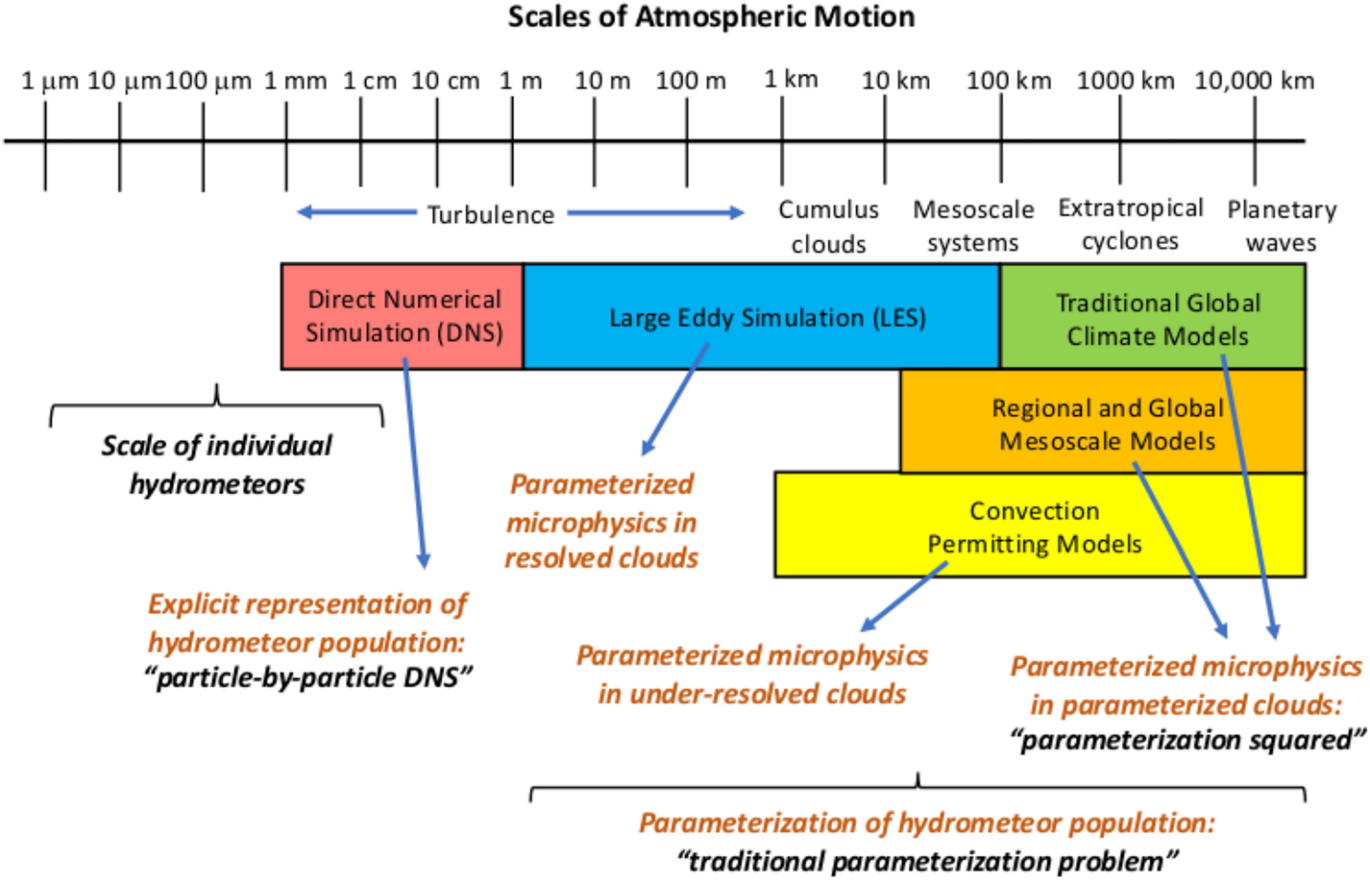
Hierarchy of atmospheric models and the scales of atmospheric motion they represent (colored boxes), inspired by Krueger (2000). The hydrometeor population is explicitly represented in particle‐by‐particle DNS but must be parameterized in all other models (the “traditional parameterization problem”). Individual clouds and their dynamical motions are increasingly unresolved moving from left to right in the diagram and are almost entirely unresolved in traditional global climate models (the “parameterization squared” problem).

*A hallmark of microphysics is its extreme complexity*. Microphysics is characterized by a large number of individual processes and pathways by which hydrometeors interact, a huge range of hydrometeor sizes and array of ice particle shapes, and complicated feedbacks between hydrometeor populations and their thermodynamic and dynamic environments over a multitude of scales (Figure 1). Cloudy air is also generally turbulent. The most complete model representation of a turbulent cloud is direct numerical simulation (DNS), considering all particles within a volume and their hydrodynamic interactions (e.g., L.‐P. Wang et al., 2009), which we will call “particle‐by‐particle DNS.” Individual hydrometeors and turbulent flow are modeled explicitly, but extremely fine resolution is required, down to at least the Kolmogorov scale (~1 mm in Earth's atmosphere). Together with the huge number of hydrometeors present in even small cloudy volumes, typically ~10^8^ in 1 m^3^, particle‐by‐particle DNS is currently limited to volumes of at most ~1 m^3^ owing to computational cost. In all other models, individual hydrometeors cannot be represented explicitly. Instead, the hydrometeor population within a grid volume must be parameterized, from large eddy simulation models (LES) with horizontal grid scale, Δ*x*, of order 10 m all the way to large‐scale models with Δ*x* of ~100 km or more (Figure 2). In these models, microphysical parameterization schemes (hereafter simply “schemes”) attempt to represent unresolved microphysical processes and hydrometeor populations statistically. This is a manifestation of the classical *parameterization problem*, in which models must represent the effects of unresolved features on the resolved‐scale model variables.

Microphysics represents one part of the broader cloud parameterization problem in weather and climate models, which has been called “a problem that refuses to die” (Randall et al., 2003). Framed within this wider context, the nature of the parameterization problem for microphysics varies widely across model types (Figure 2). Cloud‐scale motions are explicitly resolved in LES but are almost entirely unresolved in large‐scale models. In these coarse‐resolution models, parameterized microphysics must be coupled with parameterization(s) for the unresolved cloud structure and cloud dynamics—a “parameterization squared” problem. Here we lump together parameterizations for subgrid‐scale cloud structure and dynamics under the umbrella of “macrophysics,” broadly defining the term to include schemes for the cloudy fraction within a grid volume, turbulence, and moist convection (keeping in mind that as spatial scales become very fine, the distinction between *microphysics* and *macrophysics* loses meaning). Macrophysics may also include representations of subgrid‐scale distributions of cloud and precipitation water (e.g., Cheng & Xu, 2009; Larson & Griffin, 2013; Morrison & Gettelman, 2008; Zhang et al., 2002). This is important for coupling with the microphysics because grid‐averaged microphysical process rates (and cloud‐radiative effects) generally have a nonlinear dependence on cloud and precipitation properties like bulk water content (e.g., Larson et al., 2005; Pincus & Klein, 2000), but this is not a focus of the paper.

A conceptually straightforward way to address the *macrophysics* part of the cloud parameterization problem is to increase model resolution. In this way, the physical equations are solved more directly, with less influence from parameterization. Indeed, there has been a broad trend toward increased resolution in both research and operational models. With recent increases in computing power, storm‐scale models with Δ*x* of a few kilometers (often referred to as “convection‐permitting” models) are now routine at many operational weather forecast centers around the world (e.g., Benjamin et al., 2016; Clark et al., 2012; Lean et al., 2008; Milbrandt et al., 2016; Seity et al., 2010). Regional climate modeling at similar resolutions is becoming widespread (e.g., Hohenegger et al., 2008; Kendon et al., 2012; Prein et al., 2015; Rasmussen et al., 2011, 2017; Wakazuki et al., 2008). Further increases in model resolution are expected moving forward as computing power continues to increase. Thus, although the “macrophysics” part of the cloud parameterization problem is not yet dead and remains a major challenge, there is at least a path toward its demise.

Unfortunately, this is not the case for *microphysics*, for two reasons. First, the sheer number of particles is simply too large to model explicitly every hydrometeor within a cloud, even with massive advances in computing power. Even in a fairly small cloud with a volume of 1 km^3^, the total number of particles can easily exceed 10^17^. Thus, particle‐by‐particle DNS will remain confined to domains much smaller than most individual clouds, and the hydrometeor population will need to be parameterized in almost all models into the foreseeable future. Second, and perhaps even more troubling, even at the scale of individual cloud and precipitation particles, many microphysical processes are poorly understood. This is notably different from other subgrid‐scale components of atmospheric models, such as turbulence and radiation, for which complete governing equations or benchmark models are available, for example, the Navier‐Stokes equations for turbulence and line‐by‐line models for radiation. Moreover, there is no well‐defined physical scale at which microphysical processes are fully “resolved”; unlike the Kolmogorov scale for turbulence, scales all the way down to the molecular are potentially important for determining nucleation and growth of hydrometeors, especially for ice particles (see section 3.2). It follows that there are important uncertainties even in particle‐by‐particle DNS, despite these models representing all hydrometeors individually within a volume. In this respect, microphysics is arguably more similar to the parameterization of land surface or biogeochemical processes, which also suffer from inherent uncertainties associated with complex, poorly understood molecular‐scale chemical and biological processes. A paramount challenge for scheme developers is somehow to represent the extremely complicated and poorly understood web of interacting microscale chemical‐thermodynamic‐dynamic processes occurring in real clouds and precipitation that comprises microphysics. In practice, the most sophisticated microphysics scheme in any atmospheric model, even in particle‐by‐particle DNS, can only attempt to represent a small subset of these processes.

Even though all microphysics schemes are highly simplified representations of reality, they have traditionally varied widely in detail and complexity depending on the application. Here we distinguish between two basic types of applications: (1) *cloud modeling* with the purpose of studying cloud processes to improve understanding of cloud physics, nowadays almost always using models at DNS, LES, or convection‐permitting scales and typically with sophisticated microphysics schemes; and (2) *weather and climate modeling*, focusing not on details of the microphysical processes per se but rather the weather or climate metrics used to assess forecasts or simulations. Many aspects of cloud, weather, and climate modeling are sensitive to the representation of these microphysical processes (e.g., Clark et al., 2012; Gettelman et al., 2013; Posselt & Lohmann, 2009; Stein et al., 2015; Weisman et al., 2008; among many others; see also Khain et al., 2015 and references therein).

Microphysics schemes are built around a set of parameterized rate equations that attempt to represent the microscale processes acting on cloud and precipitation particles. These rate equations usually correspond to specific microphysical processes such as drop evaporation or ice particle melting. While there is some theoretical guidance, many of the rate equations are poorly constrained, especially for ice processes. This is a problem faced by microphysics schemes in all models, even particle‐by‐particle DNS. A key challenge is that individual microphysical process rates themselves are generally difficult to observe directly in natural clouds and precipitation. Although hydrometeor fluxes can be directly obtained in situ from disdrometer and remotely from Doppler radar and lidar, we emphasize the general difficulty of quantifying rates for individual microphysical processes directly from cloud and precipitation observations in natural clouds; multiple processes are often active under uncontrolled conditions, and measurements needed to obtain these rates are usually incomplete. Even in the controlled setting of a laboratory, what can be measured is often different from what is needed by schemes and sometimes the fundamental measurement itself is not yet possible. This means it has been difficult or even impossible to constrain many individual process rates in schemes directly from observations. Schemes have also become more complicated over time by including additional process complexity. This has likely been driven by increasing knowledge that many process details are important for simulation outcomes and also perhaps reflects a perceived necessity to incorporate more detail in order to model a highly complicated, nonlinear system such as microphysics (made possible by increasing computing power). This has exacerbated the problem of constraining schemes; in general, increasing the number of parameters that needs to be calibrated or “tuned” leads to increased uncertainty in model predicted variables. In part, this reflects the idea that many different combinations of parameter values in complex schemes may provide acceptable simulation results compared to available observations, which echoes previous concerns regarding land surface and hydrology models (e.g., Beven, 1993; Franks & Beven, 1997). Thus, one of our central arguments is that microphysics scheme complexity is “running ahead” of current cloud physics knowledge and the ability to constrain schemes observationally. Fundamentally, this calls into question not only the realism of these schemes at their core but whether or not in principle they are even verifiable in any kind of rigorous way. This presents a troubling picture moving forward. Quoting from Sir Karl Popper, preeminent philosopher of science in the 20th century (Popper, 1959): “In so far as a scientific statement speaks about reality, it must be falsifiable: and in so far as it is not falsifiable, it does not speak about reality” (this generalizes a well‐known statement from Einstein, 1921). Furthermore, while it is clear that microphysics schemes are largely uncertain, the degree to which they are uncertain remains mostly unquantified. Indeed, the design of most schemes has made it very difficult to quantify uncertainty systematically. This has been a critical limitation; rigorous characterization of uncertainty could provide a roadmap to guide future scheme development, as well as help motivate and focus efforts to improve knowledge of particular processes that represent the weakest link in models.

Given limited direct observational guidance, lack of a benchmark model, and the sensitivity of simulated weather and climate to microphysics, its representation in models has arguably become an impediment to reducing overall model uncertainty. For modeling that involves clouds or precipitation, the implication is that microphysics is, or will soon become, a dominant source of uncertainty even as other aspects are steadily improved, such as increasing model resolution. This also limits the utility of LES and other high‐resolution models for developing moist boundary layer and convection parameterizations for coarser resolution weather and climate models. Overall, we argue that to continue advancing models into the future will require confronting this problem head on. To do so, we must recognize specific aspects of the problem, which are detailed in section 3. We divide the problem into two main parts: (1) *how to represent the hydrometeor population* given the impossibility of modeling all hydrometeors individually in a cloud and (2) *limited cloud physics knowledge* at the scale of individual hydrometeors that contributes to process rate uncertainty. This article is intended to be forward looking; we therefore seek not only to clarify the main challenges but also to offer a roadmap to possible solutions in section 4. These ideas are centered on recent parameterization advances that address some of the practical challenges specific to microphysics, including the development of Lagrangian particle‐based schemes and improving basic cloud physics knowledge through observational advances. We also propose a more general hierarchical framework to try and deal with a core problem of parameterizing microphysics: How can we develop robust schemes with limited knowledge of the underlying physics and no benchmark model or complete set of governing equations? This task may seem very difficult, but we argue that progress can be made with recent advances in cloud models and statistical modeling tools, in conjunction with the large data sets of cloud and precipitation observations now available. A summary and broader outlook is discussed in section 5. The next section briefly discusses the history of microphysics scheme development with the goal of addressing a basic question: How did the community arrive at the current state of microphysics parameterization framed by the challenges discussed above?

## 2 A Brief History of Microphysics Scheme Developments

**Figure 3:**
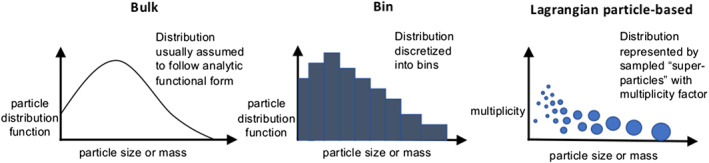
Representation of cloud and precipitation particle distributions in the three main types of microphysics schemes: Bulk (left), bin (center), and particle‐based Lagrangian (right). The horizontal axes show particle diameter or mass, and the vertical axes show the number density distribution for the bulk and bin diagrams and “multiplicity” for the Lagrangian particle‐based diagram, which is the actual number of particles that each super‐particle represents. The size of the blue super‐particles in this diagram represents the size or mass of a super‐particle. Note that almost all current bulk schemes represent particle distributions using analytic functions, although some earlier schemes did not make any assumptions about the cloud particle distribution and only considered bulk cloud water content.

Early developments of microphysics schemes in the 1950s and 1960s were broadly motivated by a desire to improve understanding of cloud processes and, at least initially, were not targeted for improving weather and climate models. At the time, operational weather forecast models and climate models had only very simple methods to calculate surface precipitation, latent heating and cooling from water phase changes, and coupling with radiation; they generally did not include any explicit representation of microphysical processes. For example, the first weather model at the US National Meteorological Center used a single moisture variable and simply removed vapor instantaneously as surface precipitation when precipitable water in the column exceeded some threshold related to the column‐mean saturation (Shuman & Hovermale, 1968). Early microphysics scheme developments followed two distinct tracks with substantially differing philosophies. One involved using simple means to portray cloud and precipitation processes and their interactions with the thermodynamics and dynamics, without attempting to include details of the microphysical processes—the “bulk” approach (left column in Figure 3). This work was pioneered by Edwin Kessler. The basic idea is well encapsulated by the following quotation published in a retrospective paper (Kessler, 1995):

“I worked with a strong sense for interactions among processes as discussed here, and in expectation that their study would be facilitated by simple means to portray microphysical processes. The first process to be considered was conversion of cloud to precipitation. How to portray it? I did little more than observe in the literature and with my own eyes that thin water clouds seem to be persistent, and that rain falls from dense clouds.”

To capture this behavior, Kessler et al. (1963) separated condensed water into two modes: *cloud water* representing small drops with negligible gravitational fall speed and assumed to follow the air motion and *rain water* representing larger drops that had appreciable fall speed and could reach the surface as precipitation. They formulated continuity equations for the bulk mass mixing ratios of cloud and rain water in addition to water vapor. Conversion of water mass between vapor and cloud occurred through evaporation and condensation, between vapor and rain through evaporation, and between cloud and rain through “autoconversion” and “accretion.” Autoconversion represented the formation of new embryo raindrops from collision‐coalescence growth of cloud droplets and depended only on the mass mixing ratio of cloud. Accretion represented the growth of existing raindrops by their collection of cloud water, formulated following the continuous collection equation and depending on both cloud and rain mass mixing ratios. The size distribution of cloud droplets within a grid volume was not explicitly considered, while the size distribution of raindrops was assumed to be inverse exponential following the well‐known observations of Marshall and Palmer (1948). This work was summarized later in an oft‐cited report (Kessler, 1969).

**Figure 4:**
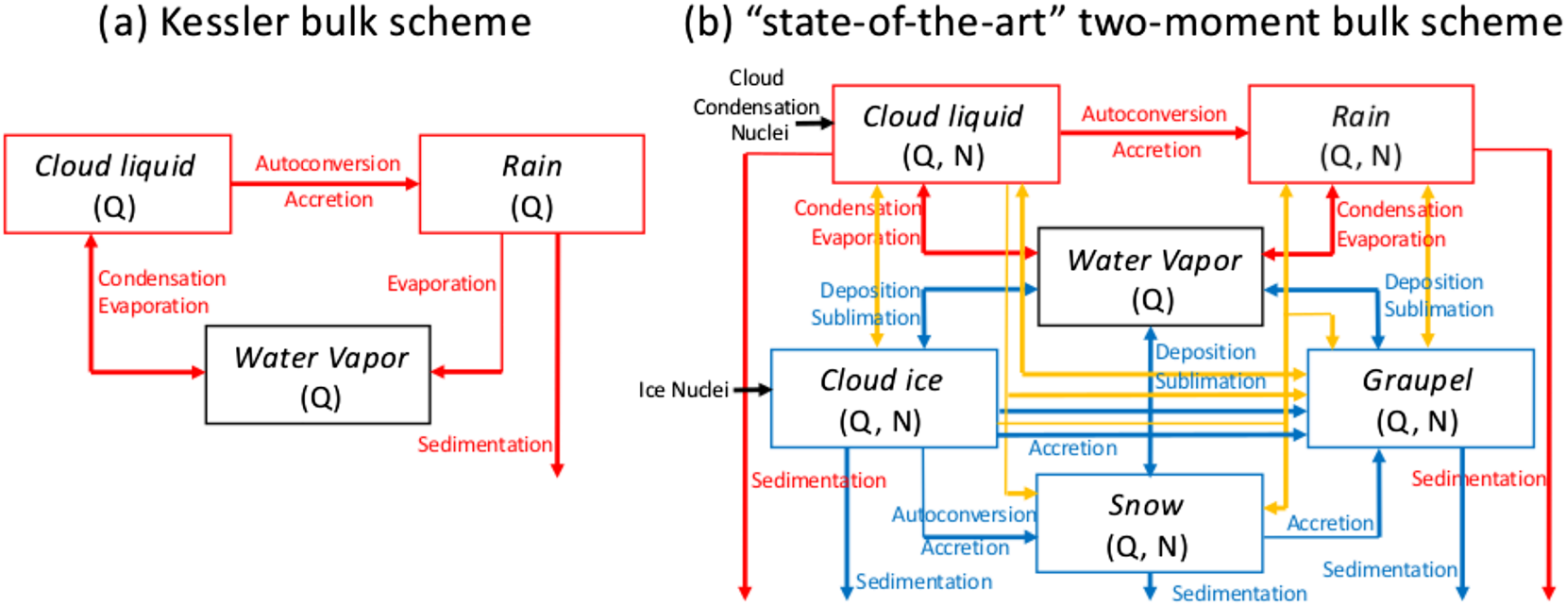
Schematic diagrams of (a) the original Kessler bulk liquid microphysics scheme, (b) a typical state‐of‐the‐art two‐moment bulk microphysics scheme. Boxes represent different hydrometeor categories (liquid and ice) and water vapor. Q and N are the mass and number mixing ratios of a category. Arrows represent microphysical processes that convert Q and/or N between categories, as well as sedimentation (fallout from gravity). Red, yellow, and blue lines represent liquid, mixed‐phase, and ice‐phase processes. Adapted from Randall et al. (2019) (©American Meteorological Society, used with permission).

Although the early development of microphysics schemes in the 1950s–1970s was motivated from the standpoint of process modeling, bulk schemes were soon after adopted into mesoscale models. This drove further development, especially from the standpoint of predicting surface precipitation amount and type. The significant increase in scheme complexity over time is illustrated in Figure 4, which shows diagrams of the original Kessler scheme (Figure 4a) and a typical current state‐of‐the‐art bulk scheme (Figure 4b). A major development in the 1970s and 1980s was the inclusion of ice microphysics (e.g., Cotton et al., 1982; Koenig & Murray, 1976; Lin et al., 1983; Rutledge & Hobbs, 1984). This had important effects on simulations owing to large impacts on sedimentation fluxes (for a given particle mass, low density snowflakes fall much slower than raindrops) and dynamics through the effects of latent heating from freezing and cooling from melting (e.g., Fovell & Ogura, 1988; Gao et al., 2006; Liu, Kogan, et al., 1997; Lord et al., 1984; McCumber et al., 1991; and many others). Including ice microphysics in a realistic way was a major challenge because of the wide variety of ice particle shapes and types in the atmosphere. To represent different ice particle characteristics, these bulk schemes typically followed an approach analogous to the separation of cloud and rain by Kessler, with most schemes including a small ice mode (cloud ice), low‐density precipitating ice (snow), and also often rimed ice (graupel or hail). These category‐based approaches addressed the practical challenges of representing ice—representing particles with different fall speeds, dominant growth processes, and so forth—and could produce reasonable results when compared to observations. However, this also introduced some conceptual problems. Separating ice into predefined categories corresponding to specific ice types necessitated conversion processes between categories—for example, the conversion of snow to graupel due to riming—and this has typically been treated in ad hoc ways. Smaller ice particles can grow to precipitating ice particles by a variety of processes (vapor deposition, aggregation, and riming), in contrast to the fairly clean separation of cloud droplets that grow mainly by vapor diffusion and rain drops that grow mainly by collision‐coalescence (in nature and in microphysics schemes). Conversion from one category to another also results in large, discrete changes in bulk particle properties such as density and fall speed, in contrast with the continuous evolution of real ice particles. Correspondingly, many studies have shown large sensitivity of simulations to how ice is partitioned among categories and to the bulk properties assumed for a given category (e.g., Adams‐Selin et al., 2013; Bryan & Morrison, 2012; McCumber et al., 1991; Morrison & Milbrandt, 2011; van den Heever & Cotton, 2004; van Weverberg et al., 2012; and many others).

**Figure 5:**
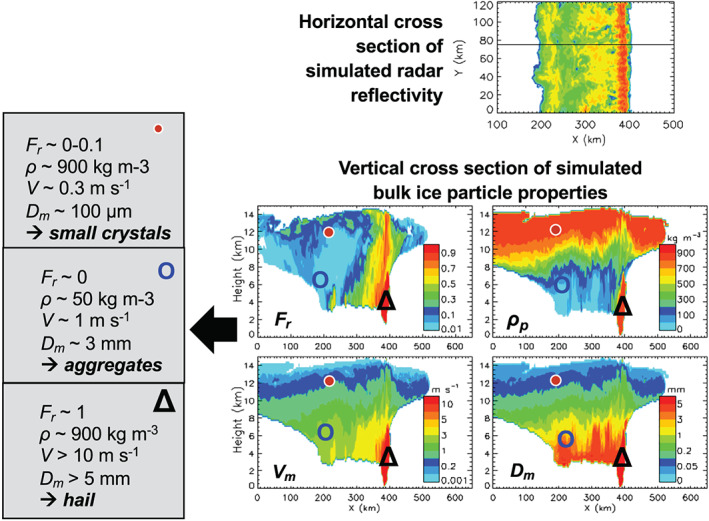
Results from a three‐dimensional simulation of an idealized squall line using the WRF model (with a 1‐km horizontal grid spacing) with the single‐ice category version of the predicted particle properties (P3) microphysics scheme (Morrison & Milbrandt, 2015). The top panel shows a horizontal cross section of simulated radar reflectivity at 1.1 km height. The four panels in the lower right show vertical cross sections of various predicted bulk ice particle properties (taken along the black line in the top panel): Rime mass fraction (*F*_*r*_), and mass‐weighted mean ice particle density (*ρ*_*p*_), fallspeed (*V*_*m*_), and diameter (*D*_*m*_). *Diagnosed* ice particle types corresponding to the *predicted* bulk particle properties are shown in the gray boxes to the left at the locations indicated by the symbols in the cross‐section plots (red circle, open blue circle, and open black triangle). All results are shown 6 hr into the simulations. Figure adapted from Morrison et al. (2015) (©American Meteorological Society, used with permission).

A few early bulk schemes eschewed the approach of having predefined categories corresponding to particular ice types in favor of predicting crystal axis growth rates and effective crystal densities derived from growth measurements (Cotton, 1972; Hindman & Johnson, 1970, 1972; Koenig, 1971). This approach has been further expanded in the last decade by developing bulk schemes that smoothly evolve particle properties such as particle aspect ratio, rime mass fraction, liquid fraction, and density without using predefined ice categories (Cholette et al., 2019; Harrington et al., 2013; Jensen et al., 2017; Lin & Colle, 2011; Milbrandt & Morrison, 2016; Morrison & Grabowski, 2008; Morrison & Milbrandt, 2015). Two such schemes (Jensen et al., 2017; Morrison & Milbrandt, 2015) are now available in the widely used Weather Research and Forecasting (WRF) model (Skamarock et al., 2008), one of which is now (as of fall 2018) used operationally in the Canadian 2.5‐km numerical weather prediction (NWP) system (Milbrandt et al., 2016). An example of the evolution of ice particle properties for a squall line simulation using this type of scheme is shown in Figure 5. Despite using only a single category of ice, a wide variety of ice particle properties in different locations within the storm are simulated as seen in the figure.

Another major development of bulk schemes starting in the 1970s was the prediction of two quantities for each hydrometeor category, typically number and mass mixing ratios (e.g., Chen & Liu, 2004; Cohard & Pinty, 2000; Ferrier, 1994; Koenig & Murray, 1976; Lim & Hong, 2010; Meyers et al., 1997; Milbrandt & Yau, 2005a; Morrison et al., 2005; Seifert & Beheng, 2001, 2006; Thompson & Eidhammer, 2014; Ziegler, 1985). Such schemes are called *two‐moment*, reflecting the fact that predicted microphysical quantities in bulk schemes are typically moments of the particle size distribution (SD), or proportional to SD moments, where “moment” refers to a weighted integral of the SD (this follows from the standard definition of a distribution moment; see section 3). The prediction of both number and mass mixing ratios, in contrast to bulk one‐moment schemes predicting only mass mixing ratios, allowed for more flexibility and realism in representing and evolving the particle SDs. More recently, this idea was extended to three‐moment bulk schemes that predict three quantities, typically number, mass, and radar reflectivity factor (e.g., Loftus et al., 2014; Milbrandt & Yau, 2005b; Naumann & Seifert, 2016; Paukert et al., 2018; Shipway & Hill, 2012). Kogan and Belochitski (2012) developed a bulk liquid scheme that predicts five bulk quantities for the drop SD and does not have separate categories for cloud and rain.

Although process modeling was a primary driver of scheme development in the 1960s to the early 1980s, a shift toward scheme development for operational weather and climate models occurred during the 1980s–1990s and has continued to the present. This shift was seen, for example, in the evolving development and use of two‐moment bulk schemes. The earliest two‐moment bulk schemes were designed to study glaciogenic cloud seeding and predicted the number concentration of all ice species but not liquid species (e.g., Koenig & Murray, 1976). Later, two‐moment schemes with linkages to modeled aerosols were developed and widely adopted in climate models to represent the inadvertent impacts of anthropogenic aerosols on clouds or “cloud aerosol interaction” (e.g., Ghan et al., 1997; Lohmann et al., 1999; Ming et al., 2007; Morrison & Gettelman, 2008). These schemes, especially in the 1990s into the early 2000s, included the number concentrations of cloud liquid but often not ice species (e.g., Ghan et al., 1997) and were correspondingly focused on the effects of hygroscopic rather than ice‐nucleating aerosol. On a historical note, this shift in focus from intentional to inadvertent modification of clouds and precipitation reflected changes in funding over the past several decades. Interested readers are referred to the National Academies report *Critical Issues in Weather Modification Research* (2003) for a concise description of the multiple factors that led to a period of cessation of federal funding for weather modification research. In brief, the initial promise of glaciogenic cloud seeding first identified in the late 1940s led to rapid commercialization and claims of positive results that were ultimately deemed unsupportable by the late 1970s. The 2003 National Academies report reached the same conclusion that a precursor such report reached nearly 30 years prior: More research is both needed and warranted. Unfortunately, the government funding gap can be viewed as a regrettable setback insofar as the long list of outstanding scientific questions relevant for weather modification identified in the 2003 report can be read nearly verbatim as those that also remain outstanding regarding aerosol‐cloud interactions relevant for climate.

Over the past 10 years, more sophisticated bulk schemes, in particular detailed two‐moment (or partial two‐moment) schemes, have also been implemented in operational high‐resolution (kilometer‐scale horizontal grid spacing) NWP systems (e.g., Benjamin et al., 2016; Milbrandt et al., 2016; Vié et al., 2016). At this scale, models begin to partially resolve convective updrafts. Thus, since microphysics schemes directly influence convective and cloud scale motions in these models through latent heating/cooling and the weight of condensate, it becomes conceptually appropriate to use relatively detailed schemes in this context and may be desirable despite increased computational cost. In addition to potential improvements in representing the feedback to the model dynamics, more degrees of freedom in these sophisticated schemes allows hydrometeor SDs to be modeled more flexibly and realistically. This, in principle, improves the computation of various forecast fields whose values depend on hydrometeor SDs, such as model reflectivity, mean particle diameter, and visibility. Improvements using multimoment compared to one‐moment schemes have been noted for observationally based case studies of various cloud regimes (e.g., Dawson et al., 2015; Milbrandt et al., 2010; Reisner et al., 1998; see also the discussion in Igel et al., 2015). However, despite the potential for added value, it has not been conclusively demonstrated that there is better forecast skill when using detailed rather than simpler microphysics schemes. This is likely due in part to the fact that high‐resolution NWP continues to be notoriously difficult to evaluate systematically using conventional performance metrics (e.g, Mittermaier et al., 2013). Furthermore, specialized forecast fields related directly to the microphysics scheme are typically not part of standard model evaluations, resulting in aspects of potential added value from detailed schemes to be overlooked.

Convection‐permitting model configurations have also been used recently for regional climate modeling (e.g.,Hohenegger et al., 2008; Kendon et al., 2012; Prein et al., 2015; Rasmussen et al., 2011, 2017; Wakazuki et al., 2008), and it is anticipated that global convection‐permitting weather and climate prediction models will soon come into wider use (Satoh et al., 2019; Stevens et al., 2019). The use of high‐resolution models for both weather and climate, both using the same model within a “unified” framework, has meant that the design of schemes for weather and climate models has been converging. This trend has accelerated recently with an increasing focus on “seamless prediction” across time and space scales for weather and climate (e.g., Palmer et al., 2008). This is despite the fact that fields of interest and metrics to assess schemes are often rather different for weather and climate (e.g., cloud radiative forcing for climate and surface precipitation for weather).

The second major track of scheme development, also starting in the 1950s to 1960s but largely independent of the work of Kessler and others who developed bulk schemes, sought to evolve cloud and raindrop populations explicitly ‐ the “bin” (also referred to as “spectral” or “sectional”) approach (middle column in Figure 3). This was done by numerically solving equations describing cloud and raindrop evolution that were as close to first principles as possible, keeping in mind that even at present, many microphysical processes remain poorly understood. In this approach, the drop SD (or mass distribution) was approximated by means of a discretized distribution function. The earliest efforts (e.g., Hardy, 1963; Mason & Ramanadham, 1954; Srivastava, 1967) focused on studying the evolution of a population of falling raindrops. Later, in the 1960s to the 1970s, studies used bin schemes to model drop SDs over a wide range of drop sizes, from small cloud droplets of a few microns to large rain drops (e.g., Berry, 1967; Berry & Reinhardt, 1974a, 1974b, 1974c; Kovetz & Olund, 1969; Twomey, 1964). The particular numerical methods employed varied among these studies, and reducing errors associated with numerically calculating SD evolution has been a major challenge since the inception of bin schemes. Several studies since the 1970s focused on improving numerical approaches for solving condensation and collision‐coalescence growth. For example, Egan and Mahoney (1972) developed an accurate, but expensive, method that conserved multiple moments of the drop SD during growth processes. Young (1974), Tzivion et al. (1987), and Stevens et al. (1996) proposed methods that solve separate equations for the drop mass and number mixing ratios to reduce artificial SD broadening from numerical diffusion during growth calculations. Liu, Moncrieff, et al. (1997) proposed a variational method that predicted only a single variable in each bin but conserved any number of SD moments as needed. Khain et al. (2008) used a remapping technique that conserved three moments of the SD (those corresponding to number, mass, and radar reflectivity factors).

Because bin schemes predict one or more variables in each bin, they are computationally expensive—typically at least one to two orders of magnitude more costly than bulk schemes. This has limited bin schemes to research modeling, while bulk schemes have remained the mainstay of operational weather and climate models. This substantial cost limited the use of bin schemes in earlier studies to idealized frameworks for modeling the evolution of drop SDs. With increasing computer power, process studies since the 1980s have used liquid bin schemes coupled to two‐ and three‐dimensional dynamical cloud models. These studies investigated, for example, detailed aspects of microphysics‐cloud dynamics coupling (e.g., Ackerman et al., 2004; Kogan, 1991; Stevens et al., 1996; Wyszogrodzki et al., 2013) and aerosol impacts on clouds (e.g., Feingold et al., 1996, 1999). Other work since the 1980s has incorporated ice microphysics into bin schemes (e.g., Hall, 1980), often following a category‐based approach similar to bulk schemes (e.g., Geresdi, 1998; Khain et al., 2004; Lebo & Seinfeld, 2011; Reisin et al., 1996). Bin schemes with separate ice‐phase categories suffer from similar conceptual and practical problems from using predefined ice categories as bulk schemes, though a few bin schemes have adopted the ice particle property‐type approach by predicting particle shape and density (Chen & Lamb, 1999; Hashino & Tripoli, 2007). State‐of‐the‐art mixed‐phase bin schemes representing both liquid and ice hydrometeors are now commonly used in three‐dimensional research models to simulate a variety of cloud regimes (see Khain et al., 2015 and references therein).

Bin schemes remain limited to research modeling owing to their computational cost but have been used to develop and test bulk schemes for weather and climate models (e.g., Berry & Reinhardt, 1974d; Chen & Liu, 2004; Fan et al., 2012; Feingold et al., 1998; Khairoutdinov & Kogan, 2000; Kogan, 2013; Kogan & Belochitski, 2012; Lebo et al., 2012; Morrison & Grabowski, 2007; Shipway & Hill, 2012; Seifert, 2008; among many). Several studies have formulated process rates for bulk schemes directly from bin scheme results using regression or other fitting techniques (e.g., Berry & Reinhardt, 1974d; Chen & Liu, 2004; Khairoutdinov & Kogan, 2000; Kogan, 2013; Kogan & Belochitski, 2012; Seifert, 2008). This hierarchical approach to scheme development is rooted in the idea that bin schemes provide a better representation of cloud physics than bulk schemes, with the implicit assumption that they should provide a better match to observations if other sources of model error (initial conditions or dynamics) can be minimized. Another bin‐informed approach for bulk schemes calculates the process rates by discretizing the particle SD and numerically integrating (e.g., regional atmospheric modeling system microphysics; e.g., van den Heever et al., 2006 and Saleeby & Cotton, 2008; see also Morrison & Milbrandt, 2015), which has been called the “bin‐emulating” bulk approach. Because of the computational cost, calculations are made offline and stored in lookup tables. This approach can improve accuracy of process rate calculations but is not fundamentally different from traditional bulk schemes because only a few bulk‐predicted quantities are used to evolve the particle SDs. Moreover, it is only appropriate for process rate calculations that do not have closed‐form analytic solutions.

Bin schemes certainly provide more sophistication in representing microphysical process rates, and they have many more degrees of freedom to evolve cloud and precipitation properties; however, evidence that they actually give consistently better results when compared to available observations is lacking. Given that predictability is inherently limited at cloud and convective scales and there is large case‐to‐case variability in simulation quality, a large number of individual real cases and/or ensembles may be needed to evaluate microphysics schemes rigorously through comparison with observations (Flack et al., 2019; Stanford et al., 2019). In situ observations, commonly viewed as the “gold standard” for evaluation of bin microphysics scheme SDs, are also lacking in terms of the number of cases, sufficient coverage spatiotemporally for any individual case, and adequate characterization of sample volumes (e.g., for drizzle‐sized drops). The fact that we lack rigorous assessments of whether or not bin schemes can consistently outperform bulk schemes can be viewed as a prime example of complexity outrunning the knowledge base. As we argue throughout this work, observations must remain the final arbiter.

There are important and unanswered questions about many cloud processes and their interactions that influence weather and climate. Some processes are known or suspected to be important in clouds, and they are currently neglected or treated very crudely. This has motivated an increase in complexity and sophistication in process models, but observations are often inadequate to provide the details necessary to characterize these processes quantitatively. Schemes in operational models, on the other hand, are strongly constrained by their computational cost. Greater flexibility in representing cloud microphysics using detailed bulk schemes therefore has to be balanced by the increased computational cost. As computing power has increased dramatically over time, schemes in operational weather and climate models have generally become more sophisticated, with increasingly detailed process formulations and additional predicted microphysical variables (e.g., going from one‐moment to two‐moment bulk schemes). Moreover, as noted above, the use of more sophisticated schemes becomes more appropriate conceptually as model resolution is increased. With the expectation of further advances in computing power, the trend of ever more detailed and complicated schemes is expected to continue—a “march toward complexity” so to speak. This is expected for both research and operational models, even though the basic motivation for developing more sophisticated schemes differs between the two.

**Figure 6:**
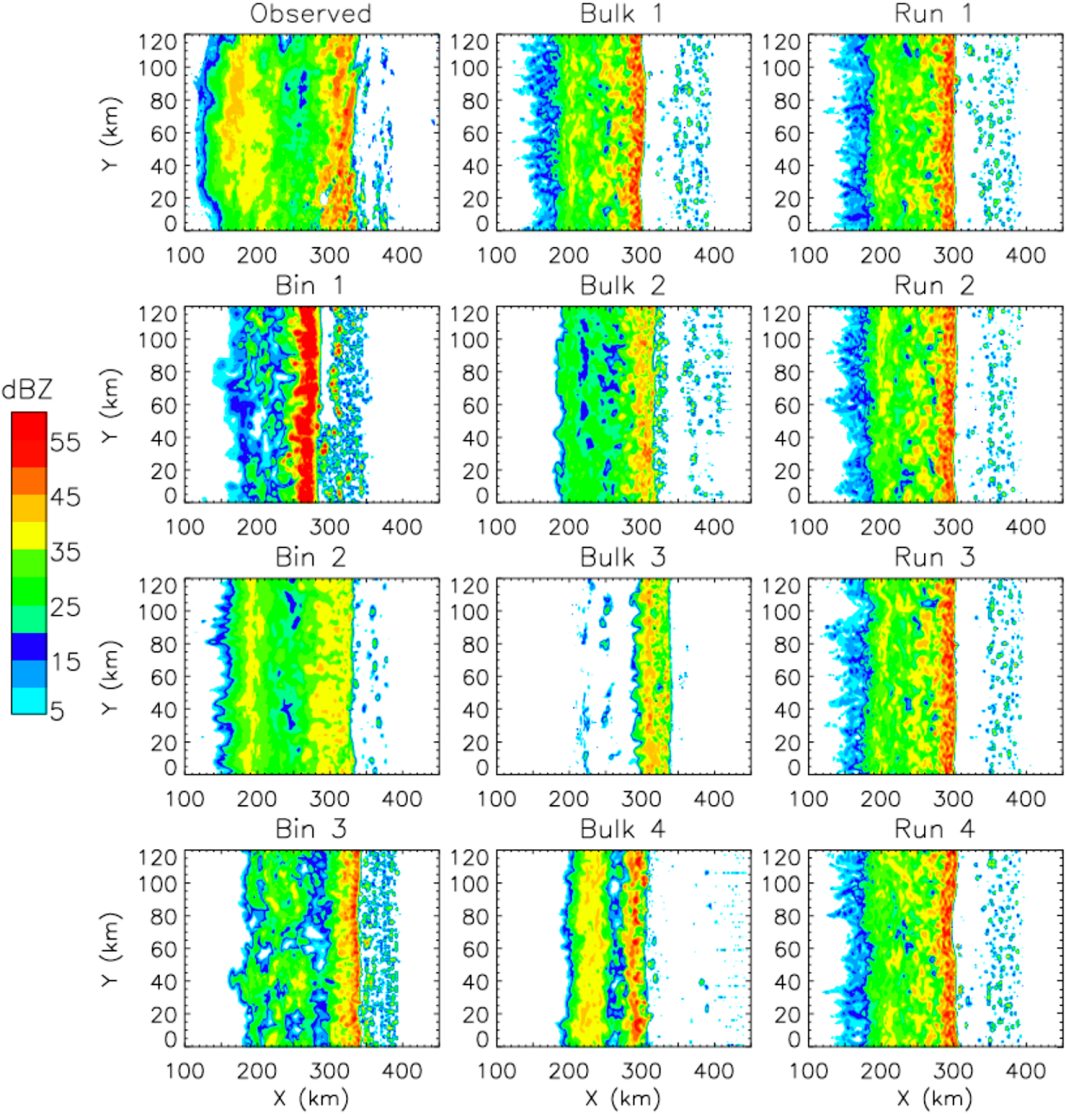
Horizontal cross sections of radar reflectivity at a height of 2 km above ground level from simulations using the WRF model of a squall line case observed in the Central United States on 20 May, 2011 during the Mid‐latitude Continental Convective Cloud Experiment (Jensen et al., 2016). The observed reflectivity is shown in the upper‐left‐most panel. Simulations using three different bin schemes are shown in the lower three left panels and four different two‐moment (or partially two‐moment) bulk schemes in the middle panels. The right panels present simulations using the “Bulk 1” scheme with small differences in the initial potential temperature field (applying different seeds for small random perturbations). Otherwise the setup is identical for all simulations and is based on a quasi‐idealized configuration with initial thermodynamic conditions from observed soundings and convection initiated by forcing low‐level horizontal convergence over the first hour. Results are shown at 6 hr (see Xue et al., 2017 for additional details of the model setup).

Overall, this underscores one of our main arguments: Even though schemes are growing increasingly complicated, there has not been a commensurate increase in fundamental knowledge of cloud physics and microphysical processes. As we discuss in section 3.2, many basic aspects of cloud physics remain highly uncertain, particularly for those related to ice‐phase microphysics. This has led to a situation in which schemes have become more and more complex over time but uncertainty has arguably not correspondingly decreased. This is supported by recent model intercomparison studies showing a lack of convergence as schemes have become more complicated. For instance, vanZanten et al. (2011) compared large eddy simulations of a precipitating shallow convection case using different bin and bulk schemes of varying complexity. They found large differences in precipitation flux and liquid water path among the bulk simulations, but—perhaps surprisingly—the spread among the bin scheme simulations was similar. Although they used different dynamical models for the simulations, vanZanten et al. (2011) attributed simulation differences primarily to the microphysics. Another example is from Xue et al. (2017), who simulated a midlatitude squall line using three different state‐of‐the‐art bin schemes in WRF. Here, we have expanded the Xue et al. (2017) study by including additional simulations using the same model setup but with four different two‐moment (or partial two‐moment) bulk microphysics schemes. We emphasize that all aspects of the setup are identical other than the microphysics scheme in all simulations, except for an additional ensemble using one of the bulk schemes but with different seeds to generate small (up to ± 0.1 K) random grid‐scale perturbations to the initial low‐level potential temperature field. This ensemble allows us to assess the robustness of impacts from using different microphysics schemes. See Xue et al. (2017) for other details of the model setup. Results are illustrated in Figure 6. There are large differences within the bulk (middle panels) *and* bin (left lower three panels) groupings in simulated storm structure. For example, some bulk and bin simulations produce little stratiform precipitation and others extensive stratiform precipitation, and there are large differences in the width and intensity of heavy convective precipitation. The location of the leading storm edge differs by about 40–60 km within both the bulk and bin groupings. These differences are robust and are *much* larger than differences within the ensemble using the same microphysics scheme but different random number seeds for perturbations to the initial potential temperature (seen by the four simulations in the right panels). Xue et al. (2017) attributed large differences among the bin simulations mainly to the various representations of ice particle properties and processes, ultimately tracing back to uncertainty in knowledge of ice microphysics. Differences in the representation of ice microphysics also likely contribute substantially to the differences among the bulk simulations in Figure 6.

A much different approach for parameterizing microphysical processes in dynamical models compared to traditional bulk and bin schemes has emerged since the mid‐2000s: the Lagrangian particle‐based approach (right column in Figure 3). Lagrangian particle‐based schemes within two‐dimensional and three‐dimensional models were initially used to study ice clouds including contrails and gravity wave‐generated cirrus (Jensen & Pfister, 2004; Paoli et al., 2004; Shirgaonkar & Lele, 2006). Independent of these studies, Andrejczuk et al. (2008, 2010), Shima et al. (2009), and Riechelmann et al. (2012) developed schemes for condensation and collision‐coalescence growth of drops in warm liquid clouds, while Sölch and Kärcher (2010) developed a scheme for cirrus clouds. Particle‐based schemes have a similar level of complexity in representing the hydrometeor population as bin schemes, and they also predict the evolution of particle SD (or mass distributions) explicitly. The essential difference between the two methods is in how particles are represented. In bin schemes, an Eulerian approach is used, and the particle distribution functions are predicted using continuous‐medium, density‐like microphysical variables (most commonly mixing ratios). In particle‐based schemes, the population of real particles is approximated by a sampling of point particles that move in the model‐predicted flow based on Lagrangian trajectories; these sampled particles are referred to as “super‐droplets” or “super‐particles.” Each super‐particle represents some multitude of actual particles, which is predicted using a “multiplicity” that is tracked with each super‐particle. In addition to position in physical space and multiplicity, other attributes that are tracked with each super‐particle represent the internal state of the particle, including wet and dry radii. Additional predicted attributes have included properties related to dissolved solute such as hygroscopicity factor to treat aerosol processing and aqueous chemistry (Jaruga & Pawlowska, 2018) and rime mass, number of monomers (primary ice crystals), and particle aspect ratio and/or density for ice particle Lagrangian schemes (Brdar & Seifert, 2018; Shima et al., 2019). The computational cost has thus far limited particle‐based schemes to fairly small‐domain cloud modeling studies, but with increasing computer power, it is anticipated that they will be used more widely in the future. Further discussion of prospects and potential applications for particle‐based schemes is given in section 4.1.

## 3 Challenges in Parameterizing Cloud Microphysics

Before going into more detail on the specific challenges of representing microphysics in models, we provide some additional background on exactly what microphysics schemes do and how they work. First, we consider the most general form of the kinetic microphysics equation that describes the evolution of a hydrometeor population through various microphysical processes and transport via air motion and gravitational fallout. This is expressed mathematically as

(1) $$\frac{\partial f}{\partial t}+u\cdot \nabla f-\frac{1}{\rho }\frac{\partial \left(\mathrm{\rho Vf}\right)}{\partial z}={\left(\frac{\partial f}{\partial t}\right)}_{\text{diff}}+P_{1}+P_{2}+\ldots P_{N},$$

where *f* ≡ *f*(**x**, *t*, **q**) is a distribution function that describes the hydrometeor population and depends on location in physical space **x**, time *t*, and a vector **q** representing one or dimensions associated with particle attributes or measures; in schemes, most commonly particle radius (thus, representing the size distribution or SD) or mass but potentially including additional dimensions for attributes such as ice particle aspect ratio or dissolved solute mass. In Equation 1, ${\left(\frac{\partial f}{\partial t}\right)}_{\text{diff}}$ is diffusion in physical space, **u** is the wind vector, *ρ* is air density, *V* is the particle fallspeed, and *P*_1_, *P*_2_, …, *P*_*N*_ are the *N* individual microphysical process rates affecting *f* (condensation, freezing, etc.). Processes involving water phase changes consequently affect temperature via latent heating or cooling.

The basic task of a microphysics scheme together with its parent model is to solve Equation 1 numerically. In the standard Eulerian bin and bulk approaches, Equation 1 is solved by predicting a set of *microphysical state variables* related to *f*. In *bin schemes*, *f* is discretized over **x**, *t*, and **q**, where the space of **q** is usually represented by a single dimension of particle radius or mass, or rarely as a two‐dimensional (or more) space, for example, drop mass and dissolved solute mass (e.g., Lebo & Seinfeld, 2011) or, for ice, the particle aspect ratio (Chen & Lamb, 1999; Misumi et al., 2010). The microphysical state variables are the mass and/or number mixing ratios over the intervals of the size or mass grid.

In *bulk schemes*, the state variables are bulk hydrometeor properties that depend only on **x** and *t*, such as the mass mixing ratio, for one or more hydrometeor categories. These state variables can usually be expressed as weighted integrals, or moments, of *f* over the vector **q**, that is, $M_{k}=\int_{q_{\min}}^{q_{\max}}q^{k}f\left(q\right)\mathrm{dq}$ for the *k*th order moment of *f* for a single dimension in **q** (*q*_min_ and *q*_max_ define the bounds of the distribution function in *q*). Bulk schemes must therefore describe the evolution of the SD using a limited number of predicted variables and have relatively few degrees of freedom. Because the rate of change of a predicted moment of a given order from a microphysical process generally depends on moments of other orders, bulk microphysics represents a closure problem conceptually similar to the problem of subgrid‐scale turbulence closure (Kogan & Belochitski, 2012). Closure is typically, but not always, provided in bulk schemes by assuming an analytic functional form for *f*, most commonly gamma or lognormal.

In contrast to bulk and bin schemes, *Lagrangian particle‐based schemes* replace the *partial* differential equation in Equation 1 with a set of *ordinary* differential equations that evolve a collection of super‐particles. These schemes solve the Lagrangian derivative $\frac{d}{\mathrm{dt}}\;=\;\frac{\partial }{\partial t}+\left(u+V\hat{k}\right)\cdot \nabla$ following individual super‐particle trajectories on the left hand side (see section 2 in Shima et al., 2009 for a discussion of the governing equations). Each super‐particle represents a multitude of actual hydrometeors based on the “multiplicity” tracked with each super‐particle. Mathematically, the other attributes tracked with each super‐particle, such as size, mass, and aspect ratio (see section 2), correspond to the dimensions in the space of **q**.

**Figure 7:**
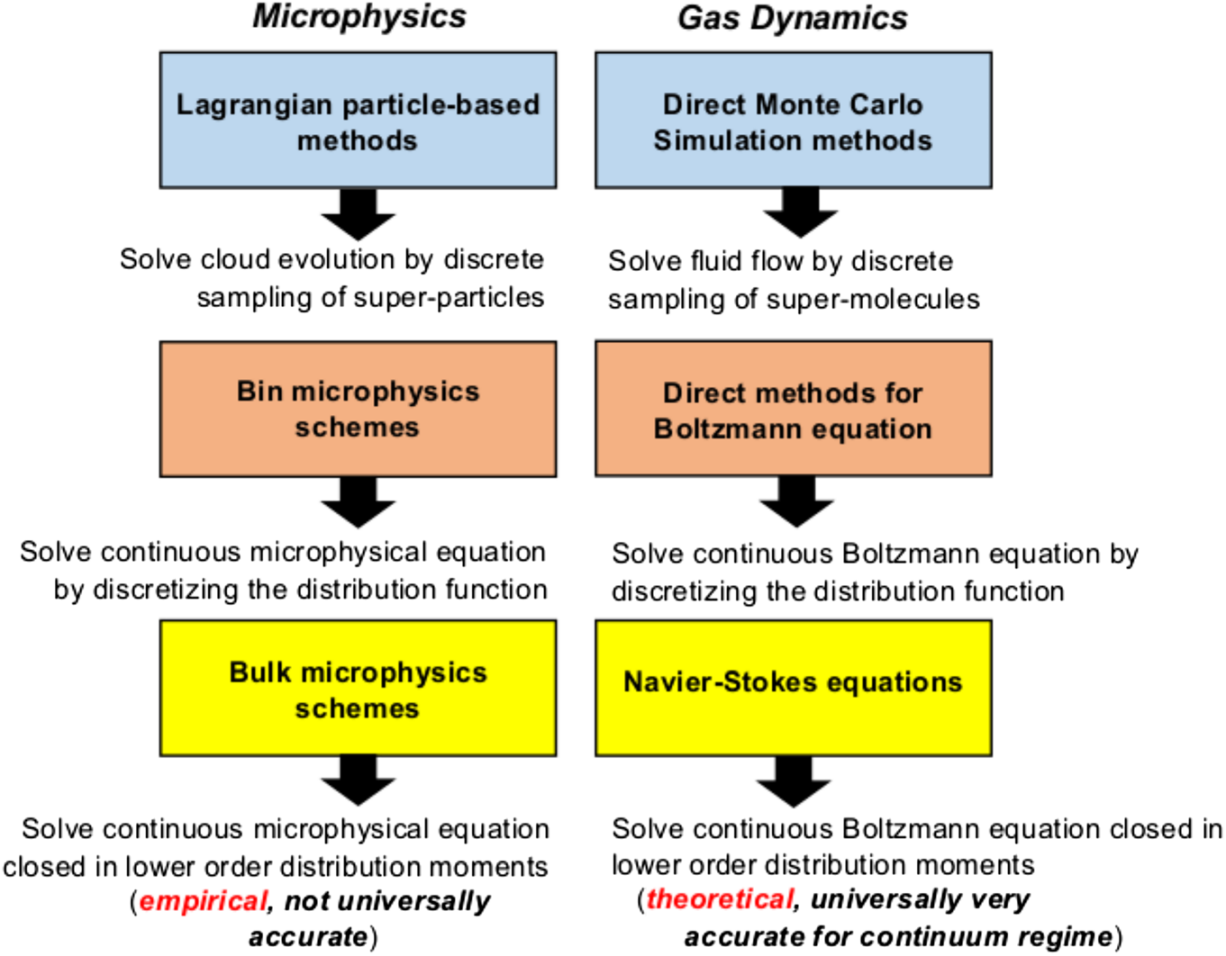
Diagram illustrating similarities and differences between methods for modeling microphysical evolution and gas dynamics. There is a close correspondence of methods for microphysics and gas dynamics in the blue and red boxes. However, even though the bulk microphysics approach and Navier‐Stokes equations are both simplified forms of the continuous kinetic microphysical and Boltzmann equations closed in the distribution moments, this closure is empirical and not universally accurate for bulk microphysics schemes whereas it is theoretical and universally very accurate (in the continuum flow regime) for the Navier‐Stokes equations.

The kinetic microphysical equation expressed by Equation 1 is very similar to the Boltzmann transport equation that describes gas dynamics, which has been noted previously (e.g., Berry, 1969). As such, there are common features of the methods used to solve these equations, as well as important differences, which are summarized in Figure 7. Lagrangian particle‐based schemes are a close analogy to the direct Monte Carlo simulation approach, primarily used to simulate rarefied gas flow, which uses simulation “molecules” that each represent a multitude of real molecules to model the flow probabilistically (Bird, 1963). Similarly, bin microphysics schemes are analogous to methods that directly solve the Boltzmann equation, again primarily used for modeling rarefied gas flows, by discretizing the distribution function in velocity and physical space (e.g., Aristov et al., 2019). However, moving downward in Figure 7, the analogy with gas dynamics ends with further simplification of the equations. The Navier‐Stokes equations, which very accurately describe fluid flow in the continuum regime (valid for Earth's atmosphere at heights up to roughly 500 km), can be derived from the Boltzmann equation through reductive perturbation expansion (e.g., Chapman & Cowling, 1970) or the renormalization group method (Kunihiro & Tsumura, 2006). These equations are closed in the lower moments of the distribution function (density, momentum, and energy) by assuming local equilibrium is satisfied and hence the distribution function is Gaussian. Unfortunately, there is no bulk microphysics analog of the Navier‐Stokes equations because, unlike the Boltzmann equation, no analytic distribution function has been derived theoretically that can well describe SDs universally. There has been work on theoretically deriving analytic functional forms for SDs based on the principle of maximum entropy (Liu et al., 1995; Wu & McFarquhar, 2018; Yano, Heymsfield, et al., 2016; Zhang & Zheng, 1994) or by treating the SD as an open system at steady state with a throughput of condensed mass in a “cascade” through class sizes (Garrett, 2019). However, it remains to be seen how well these generally describe observed or numerically simulated SDs. Thus, bulk microphysics schemes must rely on simplified assumptions about the SD form to derive the closed form of Equation 1 that they solve, contributing to uncertainty in these schemes.

In the remainder of this section, we discuss particular challenges related to the parameterization of microphysics. In doing so, we distinguish between challenges arising from poor understanding of many of the individual microphysical processes acting on hydrometeors and those related to the classical *parameterization problem*, the inability of all models (including those with bulk, bin, or Lagrangian particle‐based schemes), except particle‐by‐particle DNS, to simulate all hydrometeors individually within a cloud. This distinction follows from the discussion in the introduction and is also clear mathematically in the context of Equation 1: challenges related to process uncertainty center on limited knowledge of the process rates *P*_1_, *P*_2_, …, *P*_*N*_ in Equation 1, whereas those associated with the classical parameterization problem arise from how the distribution function *f* in Equation 1 is represented numerically.

### 3.1 Numerical Challenges of Bulk and Bin Schemes

The development of methods to solve Equation 1 has been a major effort since the inception of microphysics schemes. All methods, other than particle‐by‐particle DNS, are faced with the challenge of parameterizing the hydrometeor population within a grid volume using a limited number of predicted quantities and thus vastly fewer degrees of freedom than if one were to model all hydrometeors individually. Essentially, the critical question is how to best solve Equation 1, accurately and in a way that is computationally tractable, for a given application?

There is limited theoretical guidance on the general form of SDs. Thus, traditional Eulerian methods to solve Equation 1 use either the bin approach, discretizing the particle distribution function in size (or mass) and physical space, or the bulk approach, which solves a simplified form of Equation 1 closed with a limited number of predicted variables and typically assuming an analytic SD functional form (note that a few bulk schemes use empirically derived relationships between SD moments rather than analytic SD forms; see, e.g., Szyrmer et al., 2005). For a detailed review of bulk and bin methods, see Khain et al. (2015). Part of the reason for limited theoretical guidance on SDs is because even under idealized conditions, neglecting all processes other than collision‐coalescence, with this simplified form of Equation 1 referred to as the Smoluchowski coagulation equation (Smoluchowski, 1916) or kinetic collection equation, analytic solutions are possible only for very simple collision kernels (e.g., Drake, 1972; Long, 1974; Scott, 1968) (collision kernels are mathematical functions that describe the rate of particle collisions, defined by the ratio of collision rate to the concentration of particle pairs). These solutions do not reflect SD behavior under more realistic conditions. In the remainder of this subsection, we focus on numerical challenges using traditional bin and bulk approaches. Many of these numerical challenges fall under the purview of “physics‐dynamics coupling,” which broadly encompasses the conceptual and numerical problems arising from coupling model dynamics with physics parameterizations (see Gross et al., 2018 and references therein). Most of these problems are resolved, or at least limited, by Lagrangian particle‐based schemes, which are discussed further in section 4.1.

In general, the set of model dynamic/thermodynamic and parameterized microphysical equations exhibits stiffness; that is, sometimes processes with very short time scales are dominant (such as condensation growth of water drops), leading to rapid evolution of hydrometeor populations, while other times, slowly varying processes are dominant (e.g., generation of supersaturation by slow ascent). This problem is usually addressed by using short time steps for the time integration within schemes. This is generally not problematic in cloud or mesoscale models that use short time steps for the model dynamics anyway but becomes a major challenge in large‐scale models, especially global climate models, that have time steps of several minutes to even tens of minutes. This has sometimes been addressed by substepping the microphysics and calling schemes multiple times within the full model time step (e.g., Gettelman & Morrison, 2015; Thayer‐Calder et al., 2015). There have also been efforts to employ implicit numerical methods to evolve predicted microphysical variables for some processes (e.g., Forbes et al., 2011; Lou et al., 2012).

There are many problems related to scheme numerics and consistency with transport of microphysical variables in physical space, from both advection by air motion and sedimentation, that are beyond the scope of this paper. Nonetheless, we mention this aspect of “physics‐dynamics coupling” to point out a practical challenge; scheme developers need to be reasonably well‐versed with details of model numerics and advection schemes to minimize problems with scheme implementation. We highlight a few examples. For multimoment bulk schemes, SDs are determined by two or more predicted microphysical variables (e.g., mass and number mixing ratios). Inconsistencies between these variables can arise from advection or diffusion calculations, producing unrealistic SD properties such as mean particle size. This is one example of a broader problem in models related to inconsistencies in advecting interrelated tracer quantities (e.g., Lauritzen & Thuburn, 2012; McGraw, 2007). To our knowledge, in all multimoment microphysics schemes, this problem is dealt with by artificially adjusting the predicted variables (usually number mixing ratio) to keep SD properties within physically reasonable ranges. The nature of this problem depends on model details, such as the particular numerical method used by the advection scheme, and hence is rather complicated. In general, using a monotonic (nonoscillatory) advection scheme helps to limit these inconsistencies (H. Wang et al., 2009). Careful consideration of which microphysical variables to predict and advect can also limit errors in important SD properties, such as the spectral shape in three‐moment bulk schemes, derived from these predicted variables (Morrison et al., 2016; Paukert et al., 2018). With many more predicted variables describing the SD in bin schemes, problems related to inconsistencies among the predicted microphysical variables are even more complicated. For example, advection of individual bin microphysical variables will generally not produce consistent evolution of the bulk mass mixing ratio; that is, summing the bins to calculate the bulk mass mixing ratio first within a time step and then advecting this quantity separately will generally produce different results than summing the individually advected bin variables (Ovtchinnikov & Easter, 2009).

There are several other problems related to numerical diffusion of advected quantities in bulk and bin schemes. Along cloud edges, this leads to enhanced dilution from mixing with dry air, with attendant consequences for both the microphysics and cloud dynamics (the latter, e.g., from latent cooling associated with enhanced cloud evaporation; see Grabowski, 2007). Microphysical transformations through evaporation during entrainment and turbulent mixing with dry air can lead to reductions of either droplet number or size, or both, depending on time scales of mixing and droplet evaporation (inhomogeneous vs. homogeneous mixing) (e.g., Baker et al., 1980; Lehmann et al., 2009). Such entrainment and mixing events generally occur at subgrid scales even in high‐resolution LES and are strongly influenced by numerical diffusion in addition to parameterized subgrid‐scale mixing (e.g., Jarecka et al., 2013). One mitigating approach for LES studies of shallow cloud systems is to advect the domain with the mean horizontal wind to reduce unnecessary repeated advection calculations with respect to the grid (e.g., Fridlind et al., 2012), but this only partially addresses cloud lateral edges and does not address unresolved processes near cloud top (e.g., Mellado, 2010). The role of numerical diffusion makes it challenging to develop consistent representations of microphysical transformations during mixing, though recent progress has been made in this area that is rooted in scaling up results from DNS (e.g., Andrejczuk et al., 2009; Jarecka et al., 2013).

Another important aspect of mixing concerns its impact on the evolution of modeled SDs, particularly for bin microphysics schemes that explicitly evolve the SD shape and width. It is well known that observed SDs are generally much broader than what would occur from droplet diffusional growth alone in an ascending air parcel without mixing (e.g., Jensen et al., 1985), and the specific mechanisms governing this broadening remain a key topic in cloud physics research. It is unclear how well bin schemes are able to capture these mechanisms or distinguish them from numerical broadening. Modeling evidence (Cooper, 1989; Grabowski & Abade, 2017; Lasher‐Trapp et al., 2005) has suggested the role of mixing of different droplet populations that have undergone different growth histories on SD broadening, which has been referred to as “eddy hopping.” Isobaric mixing (in essence, associated with horizontal mixing) of microphysical variables in bin schemes from numerical diffusion and parameterized subgrid‐scale mixing may represent some aspects of eddy hopping, but this remains an open question and is being actively studied. Several other physical broadening mechanisms have also been proposed, and these mechanisms have been a subject of debate in cloud physics for the past several decades. Work since the 1950s has focused on the role of giant CCN leading to production of large drops and rain initiation (e.g., Feingold et al., 1999; Jensen & Nugent, 2017; Ludlam, 1951; Woodcock et al., 1971). Drop SD's can also broaden from Ostwaldt ripening, which is the preferential condensational growth of large drops compared to small ones owing to differences in saturation vapor pressure over drop surfaces from curvature and solute effects (e.g., Korolev, 1995; Wood et al., 2002). Accelerated drop growth and SD broadening can also occur from drop clustering (e.g., Shaw, 2000; Vaillancourt et al., 2002) and turbulent impacts on collision‐coalescence of similar size drops (Chandrakar, Cantrell, Kostinski, et al., 2018; Chandrakar, Cantrell, & Shaw, 2018; Chen, Yau, & Bartello, 2018; Chen, Yau, Bartello, & Xue, 2018; Chen et al., 2016). Other mechanisms involve drop dilution from entrainment followed by accelerated growth owing to reduced competition from water vapor during subsequent ascent (e.g., Telford & Chai, 1980), and asymmetry in drop SD evolution during adiabatic ascent and descent, broadening cloud SDs upon isobaric mixing (Korolev et al., 2013; Pinsky et al., 2014). Some of these mechanisms are included in some bin schemes, such as activation of giant CCN and turbulence‐enhanced collision‐coalescence, but many others are not. In particular, mechanisms centered around subgrid‐scale fluctuations of supersaturation and droplet clustering and their impacts on droplet growth have not been explicitly incorporated into bin schemes, to our knowledge.

Though isobaric mixing of bin microphysical variables from numerical diffusion and parameterized subgrid‐scale mixing *may* reflect a physical eddy hopping mechanism, this is clearly not the case for nonisobaric mixing associated with vertical transport. Because vertical transport from mixing in bin microphysics schemes implemented into Eulerian dynamical models is decoupled from and inconsistent with the growth/shrinkage of drops from adiabatic ascent/descent, this inherently leads to numerical broadening of SDs in bin schemes. In contrast, SDs become narrower for purely adiabatic condensational growth in ascending air. This artificial broadening of SDs is a direct consequence of numerical diffusion in radius/mass space from condensational growth calculations as well as numerical diffusion from vertical advection in physical space (Clark, 1974; Morrison et al., 2018). Even if condensational growth calculations are well‐resolved (e.g., by increasing the bin resolution), vertical advection can still result in numerical broadening. Conversely, when the SDs are well resolved in physical space (e.g., by increasing vertical resolution), the ability to represent SD evolution can be limited by bin resolution. It is therefore important to consider both bin resolution and spatial resolution together to minimize numerical broadening of SDs. Practically, this also depends on the type of bin scheme used; one‐moment bin schemes can readily use an arbitrary grid structure, making it easy to increase bin resolution. In contrast, modifying the bin structure in two‐moment bin schemes is generally very cumbersome when they include collision‐coalescence. Relatedly, the ability of one‐moment bin schemes to use an arbitrary bin structure means that numerical convergence for collision‐coalescence can be tested using realistic collision kernels in a straightforward way by increasing bin resolution, in contrast to two‐moment bin schemes (Lee et al., 2019). Numerical broadening of SDs may limit the ability of bin schemes to study *physical* SD broadening mechanisms, although its practical role in fully dynamical three‐dimensional cloud simulations has not yet been established (as opposed to idealized one‐dimensional studies). This problem is specific to bin schemes; it is fundamentally related to the fact that, in essence, they must solve a four‐dimensional advection problem for the microphysical variables: transport in three dimensions of physical space and growth/shrinkage of particles in radius or mass space (Morrison et al., 2018).

We discuss two additional problems pertinent to bin microphysics schemes (see also Grabowski et al., 2019). The first concerns a fundamental problem with the nature of the Smoluchowski (collision‐coalescence) equation, which is the equation that bin microphysics schemes solve numerically. If particles are always well‐mixed by turbulence, then collision‐coalescence can be regarded as a Markovian stochastic process. Moreover, if fluctuations in the number density of different‐sized drops are locally uncorrelated, then collision‐coalescence is well described by the Smoluchowski equation, which is a mean field equation (Gillepsie, 1972). However, if these assumptions are violated and small‐scale statistical fluctuations of the SD are important, then the Smoluchowski equation (and by extension, bin schemes that solve this equation) cannot represent the true evolution of SDs (see Dziekan & Pawlowska, 2017 and references therein). Whether or not these assumptions are valid depends on conditions such as turbulence intensity and the collision‐coalescence time scale, which determine the well‐mixed volume (Grabowski et al., 2019). It follows that bin schemes solving the Smoluchowski equation cannot represent the impact of “lucky” drops on precipitation formation—those drops that happen to collect more mass than other drops of the same initial size—which could be critical for rapid precipitation onset in warm liquid clouds (e.g., Kostinski & Shaw, 2005; Wilkinson, 2016). Indeed, the inability of bin schemes to represent stochastic fluctuations around the mean has limited the ability of researchers to investigate the role of lucky drops from a modeling perspective.

The second problem is the “curse of dimensionality.” Most bin schemes are one dimensional, in that they predict evolution of the particle distribution based on a single measure, typically drop size or mass. However, to describe cloud properties in a more complete way often requires prediction of multiple *attributes* of the hydrometeor population, with each attribute needing an additional dimension. This is particularly true for ice particles, which can take on a variety of shapes and characteristics such as density. Even for liquid microphysics, modeling aerosol processing by clouds in a rigorous way requires at least two dimensions: one for the mass of dissolved solute in drops and one for drop size/mass. Each attribute and corresponding dimension adds considerably to the computational cost of bin schemes; for *N* bins and *d* attributes, the cost scales approximately as *N* ^2*d*^. This becomes computationally intractable for *d* > 2 for bin schemes that typically have *N* of at least 30.

In short, numerically solving the set of parameterized microphysical equations is a far‐from‐trivial problem beset by a number of challenges. The Eulerian‐based approaches used by traditional bulk and bin schemes face several problems difficult to overcome, including problems related to inconsistent evolution of microphysical variables and the coupling of schemes with advection in physical space. In section 4.1, we discuss how Lagrangian particle‐based schemes can limit or resolve many of these numerical challenges.

### 3.2 Gaps in Basic Cloud Physics Knowledge

Cloud physics research has a long and storied history reaching back to the Age of Enlightenment. It became a more quantitative, rigorous discipline starting in the mid to late 19th century with the seminal work of scientists such as John Aitken and William Thomson (later known as Lord Kelvin) and rapidly accelerated after World War II with advances in technology and increased funding. Understanding gained from this research has been at the core of developing microphysics schemes since their inception in the 1950s–1960s (see section 2). However, despite major advances over the past 100+ years, knowledge gaps remain in several key areas of cloud physics that contribute to large uncertainty in microphysics schemes.

**Figure 8:**
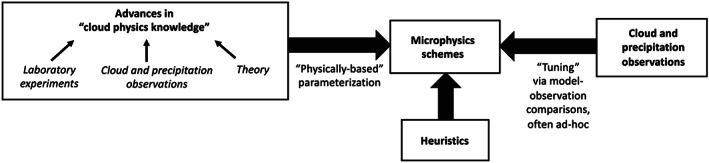
Flowchart of the traditional approach for developing microphysics schemes. Advances in cloud physics knowledge are rooted in a combination of laboratory experimentation, observations of natural clouds and precipitation, and theory. This cloud physics knowledge directly informs physically based parameterizations for microphysics scheme development. Because fundamental knowledge for many individual process rates is limited, particularly those for ice particles, heuristics play an important part in formulating many process rates. Scheme parameters are adjusted or “tuned” through comparisons of model output with cloud and precipitation observations, often in an ad hoc way.

At a basic level, understanding in cloud physics has been achieved through a combination of laboratory experimentation, observations of natural clouds and precipitation, and theory (left‐most box in Figure 8). This knowledge base has in turn served as the foundation for developing physically based process rate parameterizations in schemes. However, from the standpoint of fundamental cloud physics knowledge, all microphysical processes are uncertain to at least some degree. This is closely related to the point made in the introduction about there being no benchmark model or complete governing equation set for microphysics. In a strict sense, for microphysics, only integral constraints—essentially, water and energy conservation—are known with complete certainty. There is theoretical guidance for some individual microphysical processes but little in the way of theory for many other processes. For instance, the initial stage of cloud droplet formation on cloud condensation nuclei (CCN) is well understood based on equilibrium thermodynamics from the principle of Köhler theory. There are larger uncertainties in drop condensation after drops become large enough to have a significant fall velocity, which alters their growth by ventilation. The effects of ventilation have been characterized by laboratory studies and are represented by simple alterations to the basic diffusional growth equation (Beard & Pruppacher, 1971; Pruppacher & Rasmussen, 1979), and the parameters associated with these altered formulations are somewhat uncertain. At the other end of poor understanding are most processes related to ice‐phase microphysics, including nucleation, vapor diffusional growth, aggregation, and riming. Much of this difficulty arises because of the complicated shape and wide variety of ice particle types occurring in the atmosphere. As a result, inherently, there is more uncertainty modeling clouds containing ice than liquid‐only clouds.

In this subsection, we primarily focus on limited knowledge of microscale processes acting at the scale of individual hydrometeors. The impact of knowledge gaps in small‐scale microphysical‐dynamical interactions such as cloud entrainment and turbulent mixing was briefly discussed in section 3.1, highlighting a key modeling challenge. For completeness, we also mention another uncertain yet important aspect of cloud physics: electrification. This is an important topic for obvious reasons as a critical hazard in thunderstorms, but there is also evidence for process‐level microphysical impacts, for example, on collection efficiencies of colliding ice particles (e.g., Connolly et al., 2005; Latham & Saunders, 1970; Saunders & Wahab, 1975; Stith et al., 2014). Moreover, electrification is relevant for weather models that assimilate lightning observations. However, in the interest of brevity we will not discuss cloud electrification further.

In the following, we discuss specific gaps in cloud physics knowledge and how they contribute to scheme uncertainty. This is not meant to be a comprehensive account of all sources of microphysical process rate uncertainty in schemes but rather to highlight a few examples. These particular processes were chosen both because there is considerable uncertainty in the underlying physics and because model simulations have been shown to be sensitive to how that process is represented. Nonetheless, there are several other processes we do not discuss below but which are uncertain and can notably influence model simulations. These include warm rain initiation from cloud droplets through collision‐coalescence (including impacts of cloud turbulence), melting, collision and aggregation of ice particles, and riming growth of ice. For a more comprehensive discussion of process uncertainty, see Pruppacher and Klett (1997) and Khain and Pinsky (2018).

#### 3.2.1 Collision‐Coalescence and Breakup of Raindrops

Collision‐coalescence and breakup are key processes driving the behavior of a population of falling rain drops (e.g., Feingold et al., 1988; Hu & Srivastava, 1995; List et al., 1987; McFarquhar, 2004; Prat et al., 2012; Straub et al., 2010; Srivastava, 1967; Valdez & Young, 1985; and many others). For bin and Lagrangian particle‐based microphysics schemes, collision and breakup kernels and coalescence efficiencies are needed to represent these processes numerically. In bulk schemes, these processes are formulated by fitting rates to bin model data (e.g., Seifert, 2008) or from heuristics (e.g., Verlinde & Cotton, 1993). Model simulations have been shown to be sensitive to the representation of collision‐coalescence and breakup for some cases, via its influence on mean raindrop size (e.g., Stevens & Seifert, 2008) and hence bulk evaporation rates and cold pool characteristics (Morrison et al., 2012; Morrison & Milbrandt, 2011; Planche et al., 2019).

**Figure 9:**
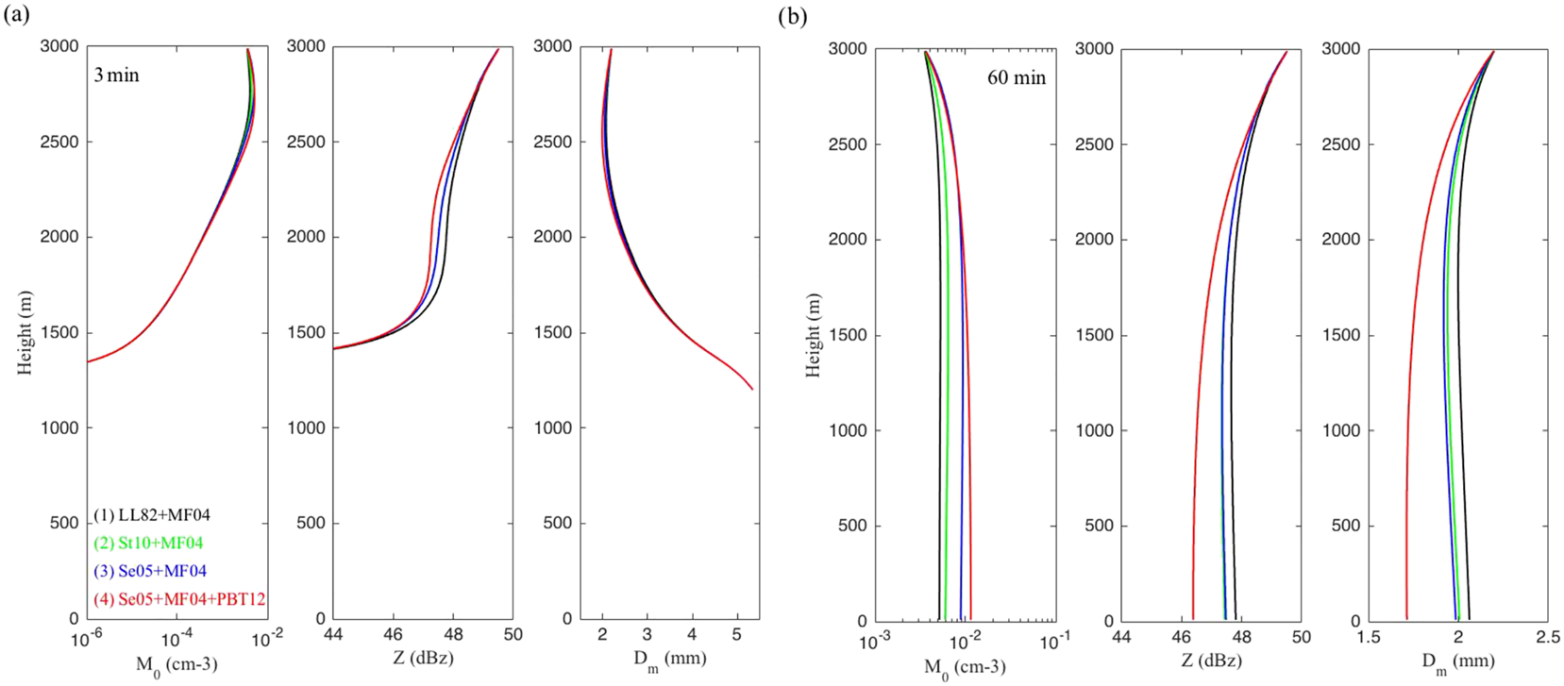
Comparison of vertical profiles of the drop number concentration (M_0_ in cm^−3^), radar reflectivity (Z in dBz), and mass‐weighted mean drop diameter (D_m_ in mm) for one‐dimensional rainshaft simulations using the bin scheme of Prat et al. (2012) with various combinations of coalescence and breakup kernel formulations (with collision‐coalescence, collisional breakup, and sedimentation as the only processes included). The formulations include (1) LL82, MF04 (black), (2) St10, MF04 (green), (3) Se05, MF04 (blue), and (4) Se05, MF04, PBT12 (red). Here LL82 (Low & List, 1982b), MF04 (McFarquhar, 2004), St10 (Straub et al., 2010), Se05 (Seifert et al., 2005), and PBT12 (Prat et al., 2012) are the formulations tested. Results are presented for a total simulated time of (a) 3 min (i.e., transient situation) and (b) 1 hr (i.e., steady state situation). The idealized simulations use an exponential drop SD with a nominal rain rate of 50 mm hr^−1^ imposed at the top of the model column.

Overall, deriving a general parameterization for drop coalescence and breakup has proven to be very difficult, and drop breakup arguably remains the most uncertain and theoretically challenging liquid microphysical process to quantify. Figure 9 illustrates the influence of different collision‐coalescence‐breakup parameterizations on vertical profiles of drop SD properties. Results are from simulations using a one‐dimensional rain shaft model with the bin microphysics scheme of Prat et al. (2012) coupled with various collision‐coalescence‐breakup parameterizations; no other microphysical processes are included. The nominal rain rate is constant throughout the column once steady state is achieved (not shown). The integral properties of the drop SD (number concentration, radar reflectivity, and mean drop size) are similar in the early transient stage at 3 min (Figure 9a) but much larger at 60 min (Figure 9b) after steady state is achieved. For example, the steady‐state mean drop size near the surface ranges from about 1.7 to 2.1 mm, and the drop number concentration varies by more than a factor of 2 (Figure 9b).

Reproducing collisional (drop‐drop) breakup in a laboratory environment presents a technical challenge. Earlier work by McTaggart‐Cowan and List (1975) and Low and List (1982a, 1982b) performed collision experiments between two drops and identified three types of breakup (disc, filament, and sheet). From a limited number (10) of colliding drop pairs, Low and List (1982b) proposed a breakup parameterization (ratio for each type of breakup and number of resulting fragments). From these laboratory experiments, they also refined expressions for coalescence efficiencies that are widely used in bin schemes (e.g., Brown, 1986, 1993; Feingold et al., 1988; Hu & Srivastava, 1995; Jacobson, 2011; List et al., 1987; List & McFarquhar, 1990; McFarquhar, 2004; Prat & Barros, 2007, 2009; Prat et al., 2012; Tzivion et al., 1989; Valdez & Young, 1985). McFarquhar (2004) used a modified Monte Carlo method with bootstrap to randomly choose the result of the collision of arbitrary pairs of drops and proposed general expressions for the parameters of the fragment distribution functions for each type of breakup. This parameterization has a more consistent physical basis than Low and List (1982b).

More recently, a large data set of binary raindrop collisions under free‐falling conditions was collected using high‐speed imaging technology (Testik et al., 2006). These experiments presented a similar fragment distribution to the original Low and List (1982a, 1982b) experiments but showed significant differences in the number of fragments produced in the smallest diameter range (*D* < 0.2 mm) when small drops (*D* ≤ 1 mm) and large drops (*D* ≥ 3 mm) collided (Barros et al., 2008). For coalescence, Seifert et al. (2005) proposed an expression that combined the Low and List (1982a) formulation for larger drops (*D* > 0.6 cm) and the Beard and Ochs (1995) expression for smaller drops (*D* < 0.3 mm), with a composite kernel for the intermediate range of diameters. In an attempt to further generalize the result of colliding raindrops, Testik (2009) proposed a theoretical delineation of the physical conditions for the occurrence of drop‐drop interaction outcomes (bounce, coalescence, and breakup) in the form of a regime diagram in the *W*_*e*_ – *R*_*d*_ plane (i.e., Weber number *W*_*e*_ vs. diameter ratio of the two interacting raindrops *R*_*d*_, where *W*_*e*_ is a dimensionless number relevant to the dynamics at the interface of two fluids that expresses the relative importance of fluid inertia to surface tension) that was further refined using the aforementioned laboratory experiments (Testik et al., 2011). Using the regime delineations in the *W*_*e*_ − *R*_*d*_ plane, a refinement of the coalescence efficiency was proposed by Prat et al. (2012).

To overcome the limitations associated with a small number of laboratory experiments, Beheng et al. (2006) used direct numerical simulation (DNS) to predict the resulting fragment size distribution of collisions among 32 drop pairs with diameters ranging from 0.35 to 4.6 mm (Schlottke et al., 2010). The new parameterization developed from these numerical experiments (Straub et al., 2010) was found to be in close agreement with other formulations derived from laboratory work (Low & List, 1982a, 1982b; McFarquhar, 2004). From the same numerical experiments, these studies derived simpler expressions for the coalescence efficiency as an exponential function of *W*_*e*_. However, bounce was not predicted by the DNS experiments (Schlottke et al., 2010), most probably because only a handful of the drop pairs simulated were located near the boundary of the bounce, coalescence, and breakup regimes. Overall, further work is needed to better quantify the outcome of drop‐drop collisions across these regimes for developing physically based parameterizations of collision‐coalescence and breakup.

#### 3.2.2 Heterogeneous Ice Nucleation

**Figure 10:**
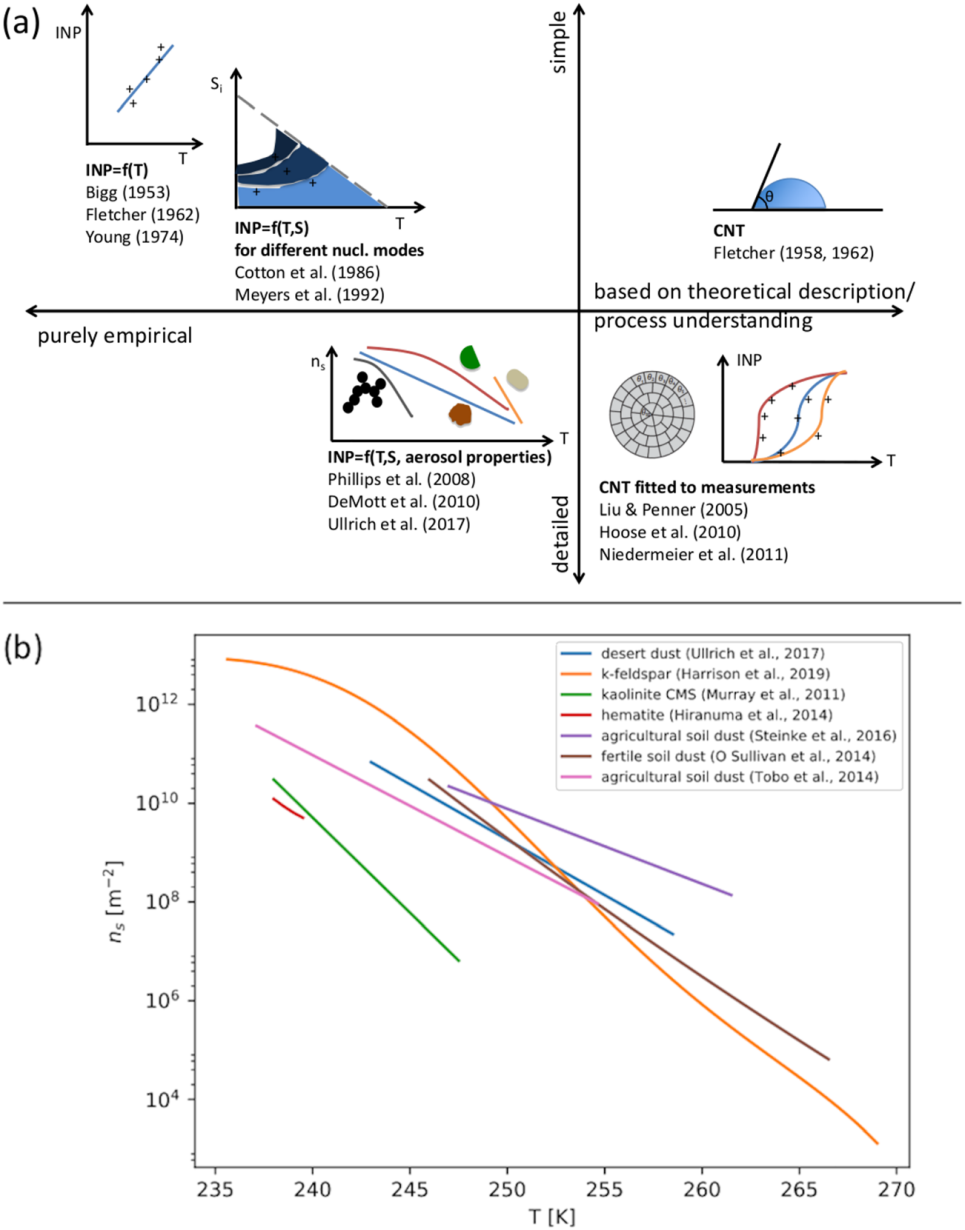
(a) Schematic of various heterogeneous ice nucleation parameterizations with different complexities. References are given as examples. Figure inlay adapted from Niedermeier et al. (2011) (under the creative commons attribution 4.0 license). “CNT” refers to classical nucleation theory (e.g., Pruppacher & Klett, 1997). (b) Recent empirical INP parameterizations as a function of temperature normalized by aerosol surface area (ice nucleation active site density *n*_*s*_) for different types of minerals, desert dust, and soil dust.

Cloud model simulations are sensitive in many cases to how ice nucleation is parameterized (e.g., Fridlind et al., 2012; Kulkarni et al., 2012; Paukert et al., 2017; Zhang et al., 2014). In several different climate models, the simulated global‐mean liquid water path, cloud forcing, cloud feedback, and for some even the climate sensitivity were found to depend strongly on the choice of the ice nucleation scheme (e.g., Barahona et al., 2010; DeMott et al., 2010; Garimella et al., 2018; Gettelman et al., 2012; Liu et al., 2012; Storelvmo et al., 2011). Atmospheric ice can nucleate homogeneously at temperatures approximately below −40°C and at higher temperatures through various heterogeneous modes. It was already recognized by the 1930s that aerosol particles heterogeneously initiating ice from the vapor phase or the crystallization of supercooled droplets (ice nucleating particles, INP) before the onset of homogeneous freezing must have special properties and that their number concentration is small but increases strongly with decreasing temperature (e.g., Bergeron, 1935). This idea was supported by the first quantitative measurements of INP concentrations in the laboratory and in the field (e.g., Schaefer, 1949). Although it was recognized that INP concentrations vary regionally and temporally, and that different aerosol types have different efficiencies in nucleating ice, earlier parameterizations used widely in models depended only on temperature or on supersaturation with respect to ice and did not distinguish between homogeneous and heterogeneous nucleation (Figure 10a, upper left). More recent parameterizations for heterogeneous ice nucleation included more detailed functional dependencies on aerosol properties (lower part of Figure 10a). Some parameterizations have also incorporated elements from classical nucleation theory (CNT, right part of Figure 10a), but this theory contains many unknown parameters related to the chemical and physical properties of INP (e.g., see Pruppacher & Klett, 1997). This description is only usable in models when these parameters are constrained based on laboratory measurements. However, experiments with different types of aerosols as INP yielded an enormous spread in ice nucleation onset conditions (Hoose & Möhler, 2012), even within a single aerosol type (e.g., mineral dust). This is due to variability in the aerosol size or surface area, surface characteristics such as roughness or pores, coatings, and detailed aspects of chemical composition such as the specific mineral type. Normalizing by aerosol surface area leads to some degree of convergence in the measured ice nucleation efficiency (Figure 10b), particularly using recent advances in INP measurement technology (DeMott et al., 2018). However, parameterizations based on these observations still require input parameters such as dust SD and dust mineralogical composition that are often not available and are difficult to generalize. Thus, although ice nucleation parameterizations have become more sophisticated and physically based, they remain subject to considerable uncertainty.

#### 3.2.3 Diffusional Growth of Ice

Understanding and quantifying the growth of ice from vapor diffusion is important for modeling the evolution of ice‐containing cloud layers. Unlike cloud droplets, ice crystals can attain relatively large sizes through vapor growth alone and can therefore directly and indirectly (through subsequent aggregation and riming) affect precipitation formation. Cloud model simulations show particular sensitivity to vapor diffusional growth because of its influence on the evolution of crystal habit (shape) in mixed‐phase clouds (e.g., Sulia et al., 2014; Woods et al., 2007). Models have also shown sensitivity of simulated cirrus properties to surface kinetic processes that influence vapor diffusional growth (Gierens et al., 2003; Zhang & Harrington, 2015). Moreover, climate simulations are known to be sensitive to crystal fall speed (e.g., Heymsfield & Donner, 1990; Sanderson et al., 2008), which is strongly influenced by vapor growth and crystal habit assumptions.

The key challenge in estimating the vapor growth of ice particles lies in the intimate connection between gas‐phase vapor and thermal energy diffusion and the surface attachment kinetic processes that ultimately determine the mass and shape evolution of crystals. Attachment kinetics include, in aggregate, all of the surface processes that contribute to mass and crystal axis growth (see Nelson, 2001 for a review). While the importance of attachment kinetics for crystal growth has long been acknowledged, including this in ice particle growth models has remained a significant challenge. Indeed, one of the primary limitations of the capacitance model for ice particle growth, which is ubiquitously used in modern microphysics schemes, is that the vapor density is assumed constant over the crystal. This implies that no surface processes occur, and because of this, the crystal shape cannot change in time (Ham, 1959; Nelson, 1994). This latter limitation is often overcome by supplementing the model with auxiliary equations to account for shape, such as empirical particle mass‐size relationships. Attachment kinetics are usually not included in applications of the capacitance model, though Koenig (1971) reduced the mass growth rates by a constant factor to account for attachment kinetics based on the measurements of Fukuta (1969). Axis‐dependent approaches (Hindman & Johnson, 1972; Todd, 1964) have used laboratory‐measured growth rates for the crystal axes, thus avoiding the capacitance model entirely. Although these models implicitly include the effects of attachment kinetics, they were developed for single crystalline ice at liquid saturation and therefore were not general enough for broad cloud modeling applications. Coupled with the challenge of including surface attachment kinetics into growth models is understanding how crystal shape evolves. Though some progress has been made on modeling single crystals, major uncertainties exist for the growth of ice with more complex shapes and at low temperatures (below −20°C). It has long been known that ice crystal habits can become complex, with “peculiar” or “irregular” forms appearing especially at low temperatures in surface (e.g., Kikuchi, 1969) and airborne (Lawson et al., 2019; Nousiainen et al., 2011; Stoelinga et al., 2007) in situ observations, and in laboratory experiments (Bailey & Hallett, 2002; Magono, 1970; Nelson & Swanson, 2019). The mass growth rates of these sorts of faceted crystals have not been measured; in fact, even the primary surface growth mechanism of atmospheric ice crystals is not presently known (Nelson, 2005). These problems ultimately lead to large uncertainty in the vapor deposition growth rates in all schemes that include ice‐phase microphysics.

#### 3.2.4 Secondary Ice Initiation

Secondary ice production (SIP), which is the generation of new ice particles through mechanisms other than primary ice nucleation on aerosol INP (or homogeneous ice nucleation), is a fundamental microphysical process. Through the modulation of ice particle number concentration, SIP can impact precipitation formation, glaciation of mixed phase clouds, longevity of ice clouds, cloud electrification, and cloud radiative properties (e.g., Connolly et al., 2006; Field et al., 2017; Jensen et al., 2018; Mansell & Ziegler, 2013). Some studies have shown large impacts of SIP on precipitation and latent heating simulated by cloud and NWP models (e.g., Clark et al., 2005; Connolly et al., 2006; Jensen et al., 2018; Qu et al., 2018), though others have shown much less sensitivity (Dearden et al., 2016). Understanding the mechanisms of SIP is needed for developing physically based parameterizations in weather prediction and climate models, but these mechanisms remain uncertain.

Even though SIP was observed in cloud chambers in early laboratory experiments (e.g., Bigg, 1957; Brewer & Palmer, 1949; Findeisen, 1940; Findeisen & Findeisen, 1943; Malkina & Zak, 1952; Puzanov & Accuratov, 1952), the geophysical significance of SIP was recognized only after the beginning of regular airborne studies of cloud microstructure in different geographical regions (e.g., Beard, 1992; Hallett et al., 1978; Hobbs & Rangno, 1985, 1989; Hobbs, 1969; Koenig, 1963, 1965; Mossop, 1970, 1985; Mossop et al., 1972; Ono, 1972; and many others). A systematically observed difference of up to five orders of magnitude between concentrations of INP and measured ice concentrations indicated a need to explain the physical processes underlying this discrepancy and thus a focus on SIP.

The first proposed mechanism to explain SIP was droplet fragmentation during freezing (e.g., Kachurin & Bekryaev, 1960; Langham & Mason, 1958; Mason & Maybank, 1960). During freezing of a cloud droplet, isolated pockets of liquid water may become trapped inside an ice shell. The expansion of water during subsequent freezing results in an increase of pressure inside the ice shell. If the pressure exceeds a critical value, then the ice shell may break into fragments. A review of the laboratory studies on droplet freezing shows a large diversity of reported results. Depending on the experimental setup, the number of fragments formed for the same size drop during its freezing may vary from zero (e.g., Johnson & Hallett, 1968; Pena et al., 1969) to a few hundred (Mason & Maybank, 1960).

Splintering during ice particle riming is another mechanism that can potentially explain SIP. Macklin (1960) observed splinter production in a small wind tunnel during the collection of droplets on an ice rod at speed of 2.5 m/s and air temperature −11°C. Latham and Mason (1961) observed freezing of droplets on a hailstone simulator, accompanied by the ejection of ice splinters. Later, Hallett and Mossop (1974) and Mossop and Hallett (1974) observed splinter formation during riming in a cloud chamber with liquid water content of ~1 g/m^3^and droplet concentration 500 cm^−3^. They found that splinter production is active in the air temperature range from −3°C to −8°C, and its rate has a pronounced maximum at an air temperature of −5°C and drop impact velocity of 2.5 m/s. In these conditions, one splinter was produced per 250 droplets of diameter > 24 mm. The phenomenon of splinter production during riming is usually referred to as the Hallett‐Mossop (HM) mechanism. Our review of the literature indicates that, with the exception of some early studies (Aufdermaur & Jonson, 1972; Hobbs & Burrows, 1966), most laboratory experiments on the HM process confirmed splinter production during riming but found different temperature ranges over which it is active. Moreover, despite several attempts to explain the cause of splintering (Choularton et al., 1978, 1980; Emersic & Connolly, 2017; Macklin, 1960), its physical mechanism remains poorly understood.

Collision of ice particles may result in their mechanical fragmentation and production of secondary ice (Langmuir, 1948). This hypothesis was based on observations of ice particle fragments collected during airborne studies (e.g., Hobbs & Farber, 1972; Takahashi, 1993) or ground based (Juisto & Weikmann, 1973). To our knowledge, there are only two laboratory works dedicated to collisional ice fragmentation (Takahashi et al., 1995; Vardiman, 1978). Collisional ice fragmentation has also been studied theoretically (e.g., Hobbs & Farber, 1972; Phillips et al., 2018; Vardiman, 1978; Yano & Phillips, 2010). Ice fragments observed in situ should be considered cautiously due to potential particle breakup artifacts induced by the instrument sampling. Overall, the role of the ice‐ice collisional fragmentation in SIP remains uncertain.

When an ice crystal collides with a supercooled drop, it will experience thermal shock due to latent heating of the freezing drop. This will cause a differential expansion of the ice crystal and may result in its fragmentation (Koenig, 1965). During their laboratory studies, Dye and Hobbs (1968) observed that when ice crystals became attached to a freezing drop, they often broke into 5 to 10 pieces as the drop freezes. Hobbs and Farber (1972) observed in the laboratory shattering of a dendritic crystal into several pieces after contact with a 2‐mm diameter supercooled drop. This observation is of considerable interest, as it suggests that the breaking up of ice crystals that collide and nucleate supercooled drops may play an important role in increasing the concentration of ice particles. However, the efficiency of ice particle fragmentation due to thermal shock caused by rimed freezing drops remains poorly understood, and the role of this effect on SIP remains inconclusive.

Ice particle fragmentation and formation of secondary ice may occur during sublimation in subsaturated areas near cloud edges or underneath the cloud base. Oraltay and Hallett (1989), Dong et al. (1994), and Bacon et al. (1998) performed laboratory studies of sublimating ice particles at different air temperature and humidity conditions. All three studies concluded that breakup rates depend on temperature and humidity but are largely determined by the initial shape of the ice particle. Based on observations of the metamorphosis of sublimating ice particle shapes in natural clouds, Korolev and Isaac (2004) concluded that particle fragmentation during sublimation does not play an important role in SIP.

Finally, Gagin (1972) proposed a mechanism for SIP due to activation of INP in high transient supersaturation areas around freezing drops. Rosinski et al. (1972) and Gagin and Nozyce (1984) studied nucleation of INPs around suspended freezing drops with 1‐ to 2‐mm diameter. However, laboratory study of this mechanism is limited, and it remains insufficiently quantified.

Most observations of an enhanced concentration of ice particles have been attributed to the HM process. The list of these studies extends over 30 publications, so we name only a few of them here (e.g., Ono, 1971, 1972; Harris‐Hobbs & Cooper, 1987; Bower et al., 1996; and others). In these studies, the conclusions about the HM process were obtained based on the observed association with graupel and columnar ice crystals. Fewer studies attributed observations of high ice concentration to drop shattering (e.g., Braham, 1964; Koenig, 1963, 1965; Korolev et al., 2020; Lawson et al., 2017; Rangno, 2008). Ice‐ice collisional fragmentation was identified as a source of SIP in natural clouds by Hobbs and Farber (1972), Takahashi (1993), and Schwarzenboeck et al. (2009). Based on a detailed review of published studies, it is not clear how the six current hypotheses outlined above are related to SIP in natural clouds. Without understanding the roles of these various mechanisms, physically based representations of SIP in microphysics schemes remain highly uncertain.

### 3.3 Challenges in Observing Clouds and Precipitation

Cloud and precipitation observations are an essential component for gaining understanding and addressing gaps in cloud physics knowledge. They are also needed for the development and subsequent evaluation of microphysics schemes. These observations can be divided into three main categories: laboratory measurements, in situ observations of natural clouds and precipitation, and remote sensing. Each observational method has unique strengths and attendant limitations. Thus, the three main observational methods are distinct and relevant from the standpoint of scheme development and are complementary in the information they provide.

#### 3.3.1 Laboratory Studies

Laboratory experiments have been an important method for studying cloud and precipitation physics going back to the early 20th century and for constraining models since the mid‐20th century. For example, laboratory observations have been used to develop physically based parameterizations of ice particle growth from vapor diffusion (Koenig, 1971; Todd, 1964) and riming (Hindman & Johnson, 1972), as well as melting and shedding of accumulated liquid water (Rasmussen et al., 1984; Rasmussen & Heymsfield, 1987; Rasmussen & Pruppacher, 1982). Experimentation provides relatively precise control over the environmental conditions affecting any microphysical process, which is its main strength: an individual process can, to varying degrees, be isolated and controlled allowing relatively precise process rates to be extracted. This is especially true for single‐particle studies, in which varying degrees of complexity can be added to successive experiments in a controlled fashion. Such studies are vital for developing and testing theories of single‐particle formation, growth, and ablation. However, the controlled nature of single‐particle experiments is also limiting and care must be taken when applying these experimental data to an atmospheric setting. For instance, the growth of individual particles in diffusion chambers or wind tunnels is often made in ultraclean and confined conditions, a situation not encountered in the atmosphere where hydrometeors are influenced by turbulent motion, trace gases that can affect growth rates (Hallett, 1968; Kärcher et al., 2009; Kippenberger et al., 2019; Schaefer, 1949), radiative effects near cloud edges, and so on.

In contrast to single‐particle experiments, laboratory experiments can also be done with particle populations in chambers (e.g., Manchester Ice Cloud Chamber, Connolly et al., 2012; PI chamber, Chang et al., 2016; Aerosol Interactions and Dynamics in the Atmosphere chamber, AIDA, Wagner et al., 2006), thus providing direct information on population interactions. As in single‐particle experiments, care must be taken when interpreting laboratory‐derived process rates for particle populations; for example, the residence time of particles is generally much shorter than in atmospheric clouds because of the limited size of chambers. Moreover, the boundaries of experimental chambers can influence the results (so‐called “wall effects”), and although these are usually carefully scrutinized and controlled for, they are a source of uncertainty that needs to be considered. Another limitation of experiments on particle populations is that it is not possible to extract information on the microscopic (individual particle) level, so testing fundamental equations is challenging. Therefore, a combination of single‐particle and particle population laboratory studies is necessary for testing general theories in cloud microphysics.

It is also often not straightforward to use existing laboratory data to test, develop, and constrain microphysics schemes because of a mismatch in what laboratory data provide and the simplified process equations in most schemes. For instance, the measurements of Bailey and Hallett (2004) provide a wealth of data on ice crystal shapes along with axis and volume growth rates, but it is not clear how these empirical axis‐dependent growth rates can be used in numerical models that use simplified mass growth equations based on a single size parameter. Similarly, the scant measurements of melting ice particles indicate how crystal shapes change during the melting process (Kintea et al., 2015), yet it is not clear how this information can be tied to the simplified melting schemes used in current models. Furthermore, it is not always possible to measure the quantities that are required for scheme development. For example, aggregation kernels and sticking efficiencies are difficult to measure, and those measurements that do exist have relatively high uncertainty (Connolly et al., 2012). Unfortunately, measuring these quantities precisely for particle populations is not possible with current technology. Nonetheless, despite these challenges, important advances have been made recently in cloud physics laboratory work and the use of data from experiments to constrain models; see section 4.2 for further discussion.

Even though technological advances will continue to be made and may help solve some of the measurement problems mentioned above, laboratory work on a broad range of cloud physics problems has evidently declined over the past 30–40 years, especially in the United States. Indeed, a perusal of the extended abstracts from the International Conference on Cloud Physics in 1976 indicates that approximately 60 of the 150 submissions (40%) were on experimental studies. In contrast, the most recent American Meteorological Society 15th Conference on Cloud Physics (2018) did not even contain a separate section on experimentation, and a count of abstracts revealed only 28 out of 354 total presentations (8%) were related to experimentation based on a manual review of all abstracts presented at the conference, excluding papers that were withdrawn. Furthermore, a number of university cloud physics labs have closed upon the retirement of the responsible faculty members. While there may be some obvious reasons for why this decline has apparently occurred, we argue that it is worth carefully examining whether this downward trend is in fact real, and if so, how it can be reversed. Quoting again from Sir Karl Popper (1978): “Verifications are cheap: they are easy to come by if one is looking for them. The only verifications of significance are serious attempts at falsification that have not achieved their objective ….” Because they provide the most direct way to quantify individual microphysical process rates and mechanisms, laboratory studies provide the most direct means to falsify theories in cloud physics; a reduced scope of laboratory work is therefore detrimental to the cloud physics community as a whole.

#### 3.3.2 In Situ Observations of Natural Clouds and Precipitation

In situ measurements of cloud and precipitation can provide detailed information on a particle‐by‐particle basis, and aircraft in particular can deliver some of the most complete observational data sets from the perspective of colocated state and microphysical measurements within cloud. Moreover, aircraft in situ measurements remain the primary tool for validating radar retrieval algorithms and satellite products. However, in situ (and remote sensing) observations generally remain *incomplete* for directly constraining individual microphysical process rates in schemes. Thus, many studies have used in situ observations directly to constrain parameters that describe microphysical *properties* within schemes, as opposed to microphysical *processes*. For example, exponential raindrop SD parameters fit to the in situ observations of Marshall and Palmer (1948) were used in the schemes of Kessler (1969), Liu and Orville (1969), and many others. Aircraft observations from Houze et al. (1979) were used to characterize inverse exponential snow particle SD parameters as a function of temperature in the WSM6 scheme (WRF six‐class single moment; Hong et al., 2004; Hong & Lim, 2006). Parameters fit to the ground‐based measurements of ice particle fall speed from Locatelli and Hobbs (1974) have been widely used in a number of schemes. However, it is often difficult to use such observations in this way and to test schemes rigorously through comparisons with model output. Foremost, in situ observations are usually very limited in time and space, from both airborne and ground‐based platforms. This is inherently problematic when directly comparing in situ observations with simulations, especially with the growth of model initial condition errors that can lead to rapid divergence with observed cloud and precipitation features. Even statistical comparisons can be challenging with small in situ observational data sets, especially under conditions that are heterogeneous and rapidly varying. Instrument limitations must also be considered over the large dynamic range of cloud and precipitation SDs. For instance, disdrometers provide direct observations of SDs, but they are inaccurate at small (less than a few hundred microns) and very large drop sizes (several millimeters) (e.g., Thurai et al., 2011; Tokay et al., 2001).

An overview of the main airborne in situ instrumentation can be found in Wendisch and Brenguier (2008). Development of airborne in situ microphysical instrumentation is a great challenge, as the probe optics and electronics must operate in extremely harsh conditions, targeting measurements of micrometer‐size particles while traveling at 100–200 m/s. One of the most significant limitations of airborne in situ instrumentation is the low sampling volumes. Thus, for particle probes, the sampling rate at aircraft speed varies from a few cubic centimeters per second (i.e. cloud droplet probes) to several hundreds of liters per second (i.e., precipitation probes). In essence, the particle probes measure a sequence of single particles passing through their sample areas along the flight track. This imposes limitations on the minimum spatial scale of cloud measurements (typically ~10^2^–10^3^ m) so that small‐scale variability cannot be observed by conventional probes. Recent instrument advances based on different physical principles, briefly discussed in section 4.2, have started to address this problem, but the sample volume remains much smaller than that from remote sensing or typical model grid volumes. There is currently no method to obtain airborne observations of in‐cloud supersaturation with respect to liquid, which requires much more accurate methods for measuring temperature (to within 0.01°C) than possible using conventional airborne temperature sensors (typically 0.5°C). The lack of such supersaturation observations is an important gap in the area of cloud physics. Similarly, there is currently no airborne instrumentation to measure particle electric charge.

Among the most basic of all in situ microphysical measurements is simply the droplet or ice particle SD, yet the size‐dependent uncertainty in such measurements remains difficult to robustly quantify, especially at the smallest particle sizes (e.g., Korolev, 2007; McFarquhar et al., 2017). This presents a basic obstacle to investigating key processes such as ice nucleation in cirrus and mixed‐phase clouds. Another fundamental measurement that remains almost entirely lacking is that of single‐particle mass. Whereas that may be a trivial quantity for water droplets owing to their well‐defined density, ice crystal shape and density vary widely. Relatively recent deployment of ground‐based multicamera instruments is providing much‐needed advances in systematically characterizing particle shape (e.g., Garrett et al., 2012; Schönhuber et al., 2008), at least for ice larger than a couple hundred microns in length that is sedimenting to the surface. However, colocated measurements of single‐particle mass remain entirely missing, which is surprising and unfortunate given the central role of crystal mass in both modeling and remote‐sensing applications. Finally, we note there are challenges related to flying airborne instruments in intense weather such as convective storms. Airworthiness regulations limit operations of research aircrafts for cloud sampling to the pilot's radar return. This has severely limited in situ measurements within strong electrified storms with vigorous updrafts that are often associated with the presence of hailstones. To fill this gap, armored aircraft equipped with protected instrumentation, which can sustain operations in an electrified environment in the presence of hailstones, are required.

#### 3.3.3 Remote‐Sensing Observations

Over the past decade, polarimetric radars have been increasingly used to characterize bulk hydrometeor properties and SDs remotely. This is in part aided by the establishment of operational networks of polarimetric radars, such as the US network of polarimetric S‐band WSR‐88D radars, completed in 2013, and similar networks appearing in Europe, Asia, and South America. Nonspherical particles (both rain and ice) will tend to become oriented as they fall, allowing for radar polarimetry to provide information related to their aspect ratio, size, density, and concentration. Quantities such as differential reflectivity, specific differential phase, copolar correlation coefficient, and linear depolarization ratio each provide somewhat independent information related to these quantities (e.g., Bringi & Chandrasekar, 2001; Kumjian, 2018; Zrnic & Ryzhkov, 1999). Differences in the degree of resonance scattering (i.e., non‐Rayleigh scattering) for different radar frequencies allows for multifrequency scanning to yield further observational information (e.g., Bringi et al., 1986; Gaussiat et al., 2003; Kneifel et al., 2011, 2015; Kumjian et al., 2018; Tridon et al., 2017). Finally, radial velocity and Doppler spectrum width recorded by most modern radars provide dynamical information on observed storms, allowing for simultaneous microphysical and dynamical information gain (e.g., Brandes, 1977). Studies have leveraged these unique observational properties to provide increasingly accurate polarimetric estimates of rainfall (e.g., Brandes et al., 2001; Bringi et al., 2001; Cifelli et al., 2011; Giangrande & Ryzhkov, 2008; Illingworth & Caylor, 1989; Matrosov et al., 2002; Ryzhkov et al., 2005, 2014; Ryzhkov & Zrnic, 1996) and rain drop size properties (Ryzhkov & Zrnic, 2019; Vivekanandan et al., 2004; Zhang et al., 2006). Likewise, polarimetric radars have been used for qualitative studies of ice‐ and mixed‐phase processes (Andric et al., 2013; Kumjian & Lombardo, 2017; Moisseev et al., 2015; Oue et al., 2016; Schrom et al., 2015), often supported by offline theoretical or numerical scattering simulations. Quantitative retrievals of bulk ice properties have also been developed (e.g., Bukovčić et al., 2018; Posselt et al., 2015; Ryzhkov et al., 1998) but rely on assumptions of particle habit and properties that control polarimetric scattering (Schrom & Kumjian, 2018). There is an outstanding need to incorporate rigorous uncertainty quantification related to ice density, habit, and SD into these retrievals. Polarimetric rain property retrievals also make strict assumptions about the form of the SD and generally do not quantify this uncertainty but can be meaningfully compared to disdrometer and rain gauge data and do not suffer from the single‐particle scattering uncertainties of ice. Ice properties are, coincidentally, rather difficult to quantify even with in situ observations and are thus likely to remain important sources of uncertainty for radar‐based ice retrievals for the foreseeable future. While the preponderance of work using polarimetric radars to extract microphysical information speaks to the richness of these data sources, the lack of rigorously quantified uncertainty limits the utility and generality of these methods. Some approaches simplify this exercise into the labeling of the “dominant” hydrometeor species in an observed volume—these “hydrometeor classification” or “hydrometeor identification” algorithms provide some operational value in comparisons to simulations (e.g., Dolan & Rutledge, 2009; Liu & Chandrasekar, 2000; Park et al., 2009; Straka et al., 2000; Vivekanandan et al., 1999) but impose predefined categories that may have overlapping radar signatures, do not span the full range of true microphysical variability that occurs in nature, and may be inconsistent with how hydrometeor categories are defined in models. As such, their utility for evaluating and improving microphysics schemes is substantially limited. We emphasize that gaps in microphysical understanding might be better served by considering what information content radar does provide—a task requiring rigorous estimation of scattering properties and thorough evaluation of related uncertainties (Kumjian et al., 2019; Schrom & Kumjian, 2018, 2019).

Similar to polarimetric radar observations, vertically pointing radar observations have been used to estimate SD characteristics, aided by measurements of Doppler spectra that resolve particle size via differences in fall speed (Firda et al., 1999; Posselt & Mace, 2014; Posselt et al., 2017; Tridon & Battaglia, 2015; Tridon et al., 2017). Both polarimetric and vertically pointing radars have, again largely qualitatively, identified microphysical processes occurring in observational data, usually by linking spatial changes in observationally deduced particle properties to specific microphysical processes (e.g., Barrett et al., 2019; Kumjian & Lombardo, 2017; Kumjian & Prat, 2014; Kumjian & Ryzhkov, 2010, 2012; Leinonen et al., 2016; Moisseev et al., 2015; Schrom et al., 2015). However, quantifying individual process rates is very difficult. The few quantitative radar‐based process retrievals that do exist typically make strong a priori assumptions about the hydrometeor SD and/or the dominant process occuring (Tridon et al., 2017; Williams, 2016) and thus have unquantified uncertainties related to these assumptions.

Use of satellite remote‐sensing data for evaluating microphysics schemes is attractive because satellite data are available over remote areas where ground‐based observations are limited and has a long and growing observational record. However, all satellite measurements are at best indirectly related to the cloud properties of interest and as such suffer from the same forward model and retrieval limitations that were discussed for ground‐based remote sensing above. As a result, relatively few studies have attempted to use satellite observations to tune microphysical processes, with some exceptions such as Li et al. (2010), where ice aggregation was modified and aggregate particle breakup was added to better match TRMM radar profiles. Even with the most comprehensive suite of satellite observations, many cloud properties are underconstrained; in other words, multiple (sometimes very) different cloud properties may produce the same set of observations. For example, it is particularly challenging to determine the amount of liquid versus ice in the interior of clouds and to infer liquid and ice properties in mixed‐phase conditions. Furthermore, there are few measurements that are sensitive to ice particle shape, especially in the cloud interior. Beyond the difficulty of accurately mapping from measurements to microphysical variables, there are additional challenges to the effective use of satellite data to evaluate schemes. Satellite remote sensing is constrained by the spatial and temporal resolution and measurement sampling. This is important in three key respects. First, clouds in even the most high‐resolution measurements commonly exhibit subpixel variability. This means that satellite retrieval will assign a single value over a pixel, while in reality, the quantity may vary significantly. This also means that retrievals may be more robust for spatially homogeneous clouds (e.g., stratocumulus or thick cirrus) but increasingly biased for broken cloud conditions. Second, especially in the case of passive measurements, satellite retrievals of microphysical values are often constrained to a particular (often ill‐defined) depth within cloud top (Platnick, 2000; van Diedenhoven, Fridlind, et al., 2012) or pertain to column‐integrated quantities only. This is particularly a restriction in regions with persistent multilayered clouds. Third, with the exception of geostationary measurements, all satellite remote‐sensing measurements are snapshots too widely separated in time to capture the time evolution of cloud processes.

A general challenge when comparing model simulations with remote‐sensing data (ground based, airborne, and satellite) is that there is often a mismatch between what is measured and the quantities output from models. For example, nearly all two‐moment bulk microphysics schemes are centered around predicting bulk mass and particle number mixing ratios, but the latter is very difficult to obtain from remote sensing. On the other hand, quantities that are directly observed, such as the sixth moment of the particle SD from radar reflectivity factor (for particles that are small compared to the radar wavelength), or retrieved, such as cloud‐top effective radius (generally defined as three fourth times the ratio of bulk particle volume divided by bulk projected area) or column optical thickness (related to the vertically integrated bulk projected area), usually are not explicitly predicted by schemes. There are two approaches used to mitigate this problem. In the first, model output is converted to directly observable quantities via *instrument simulators*, which may or may not account for instrument noise and measurement errors. The second method is to transform remote‐sensing observations to microphysical quantities using retrievals, either for direct comparison to related quantities predicted by models or after applying observation simulators to the model output. Examples of such instrument or observation simulators are the Cloud Climate Change Initiative (Cloud_cci) satellite simulator (Eliasson et al., 2019) and CFMIP Observation Simulator Package (COSP; Bodas‐Salcedo et al., 2011; Ban‐Weiss et al., 2014). However, both instrument simulators and retrievals add uncertainty to model observation comparisons beyond the inherent uncertainties associated with the model itself, and this uncertainty is rarely well defined or quantified.

### 3.4 The Difficulty of Using Observations to Constrain Microphysical Process Rates

A fundamental obstacle to using natural cloud and precipitation observations to constrain schemes is the difficulty of harnessing these observations for quantifying individual microphysical process rates. As we emphasized earlier, process rate formulations in schemes are often highly uncertain, particularly for ice processes. This includes uncertainty in parameters contained within process rate formulations, which we will refer to as *parametric uncertainty*, as well as uncertainty in the functional forms of the process rates, which is a type of *structural uncertainty*. Given a fundamental mismatch between the need to reduce process rate uncertainty in schemes (both parametric and structural) and what natural cloud and precipitation measurements actually provide (cloud and precipitation *properties* that are the net result of various processes integrated over time and/or space), a key question arises: How can these observations be used to constrain schemes in an effective and rigorous way?

The traditional approach to scheme development uses basic cloud physics knowledge obtained from observations and theory combined with heuristics to formulate individual microphysical process rates and then adjusts or “tunes” the associated rate parameters based on comparison of model output with other observations (Figure 8). A general strategy for this type of observational constraint involves the following steps: (1) implement a new scheme or apply some modification to an existing scheme; (2) run simulations and compare output with observationally based metrics; and (3) modify one or more parameters and repeat, with the idea of reducing the mismatch between model and observations. In global climate models, the choice of parameters to tune is generally ad hoc or guided by heuristics, though some more systematic efforts have been undertaken (Hourdin et al., 2017; Schmidt et al., 2017). More systematic tuning efforts have included statistical extrapolation from limited sampling of jointly perturbed model parameters (Qian et al., 2018) or ensemble‐based optimization (Ollinaho et al., 2013). Microphysics scheme parameters are a subset of the wider set of parameters within models that are tuned, often including those in other physics parameterization schemes such as for moist convection and the planetary boundary layer. In climate models, such tuning is driven by a basic need to achieve global energy balance and reasonably realistic regional patterns of cloud radiative forcing, which are critical for running coupled ocean‐atmosphere climate models. Scheme tuning is also an important component of NWP models but with an emphasis on different metrics such as surface precipitation (e.g., McTaggart‐Cowan et al., 2019). It should be noted that such tuning does not specifically target improvements to microphysics schemes per se but rather to the end results from the model (or modeling system) for the fields of interest.

In contrast, with an emphasis on process‐level details rather than forecasting, explicit tuning is generally not employed in cloud models. The implicit assumption is that the schemes in these models employ the best available knowledge of processes and parameters relevant for the particular problem being addressed. Indeed, the objective of many such studies is to investigate whether the best available knowledge can reasonably reproduce observed conditions (see the discussion of SIP in section 3.2.4 as an example where such efforts have failed, indicating an important knowledge gap). Nonetheless, parameter modifications are often made in developing and testing schemes for cloud models, even on a case‐by‐case basis, in order to improve comparison with observations. In effect, this is similar to tuning in climate and NWP models, even if it is generally not referred to as such. Given that cloud modeling studies are usually focused on a single case, or a small handful of cases, more systematic tuning is generally not employed, though it could be.

Several challenges are encountered when using cloud and precipitation observations to constrain schemes systematically. Perhaps the most important, state‐of‐the‐art microphysics schemes in cloud, weather, and climate models are very complicated with numerous parameters and interacting processes (as illustrated in the schematic diagram to the right in Figure 4). Schemes often have 15–20 or more parameters that are explicitly considered as “parameters,” plus many additional hard‐wired numbers that are inherently uncertain but reflect a particular choice made by the scheme developer. This huge parameter space dimensionality is a major challenge when using statistical methods to estimate the set of microphysical parameters (often called “parameter estimation,” which can be thought of as similar to tuning but more rigorous and systematic). The trend toward increasing scheme complexity in weather and climate models over time—the “march toward complexity”—has made this problem even more challenging. Nonetheless, recent studies have shown some success in constraining multiple microphysical parameters simultaneously using Bayesian inference to characterize parameter probability distributions (e.g., Posselt, 2016; Posselt & Vukicevic, 2010; van Lier‐Walqui et al., 2012). While these methodologies have promise, they are limited: Only a handful of parameters are considered, synthetic rather than real observations are used, and they are limited to a small number of case studies. Furthermore, there has been little systematic effort to address structural uncertainty and constrain the *structure* of process rate formulations using cloud and precipitation observations. Some approaches have tried to account for structural uncertainty by directly perturbing physical parameterization tendencies to generate forecast ensembles (the stochastic perturbed parameterization tendency approach or SPPT; e.g., Berner et al., 2015; Jankov et al., 2017; Palmer et al., 2009) or by applying multiplicative perturbations to microphysical process tendencies (van Lier‐Walqui et al., 2014). However, these approaches have little physical basis. Multischeme ensembles have also served as an implicit method of investigating structural uncertainty, but this is not systematic, and it is not clear if such ensembles indeed meaningfully quantify any aspect of the true structural uncertainty of schemes.

There are also more practical challenges when comparing model output with cloud and precipitation observations with the goal of constraining microphysical process rates in schemes. Even if model biases can be robustly identified through comparison with observations, it can be very hard to pinpoint these biases specifically to the microphysics scheme, let alone a particular process within the scheme. There are numerous possible sources of model error, including the dynamics and numerics, initial and boundary conditions, and other physics parameterizations, as well as the coupling among all of these model components. A particularly challenging aspect of this problem concerns the rapid growth of initial condition errors for evaluating simulations of specific weather events, including case studies developed from field project observations. Data assimilation can be used to limit such errors and keep the model state close to observed, but this can lead to difficulty when interpreting model biases. Nevertheless, information about biases originating from specific model components, including the microphysics, may be obtainable within an assimilation framework (e.g., Rodwell & Palmer, 2007; Wong et al., 2019). One possible reason why such efforts have not been more widespread is simply because there has generally not been close interaction between microphysics scheme developers and the data assimilation community in the past.

## 4 Possible Paths Forward

There has been real progress in parameterizing microphysics over the past few decades, particularly for process‐level modeling. However, several key challenges remain. As we discussed in section 3, these include challenges in numerical implementation, gaps in fundamental cloud physics knowledge, and difficulty in using natural cloud and precipitation observations to constrain schemes. Many of these problems are evidently growing more difficult over time, related in part to the growing complexity of schemes. Nonetheless, there is a recent convergence of factors—the wealth of cloud and precipitation observations now available, the availability of computing power to run models with sophisticated schemes, and increasing importance of microphysics as models move toward higher resolution—that suggests an opportunity to accelerate progress. Confronting the “microphysics problem” is therefore timely and indeed it may even be necessary if we are to continue making advances and reducing overall uncertainty in models.

The need to accelerate progress calls for new and different strategies for parameterizing microphysics, and we advocate a few specific ideas and approaches in this section. This is not meant to be a comprehensive account of all possible ideas to accelerate scheme development but rather a suggestion of a few specific paths forward that address the challenges outlined in section 3.

### 4.1 Lagrangian Particle‐Based Schemes

It will be prohibitively difficult to model all hydrometeors within a cloud individually on a particle‐by‐particle basis into the foreseeable future, even for fairly small cloud volumes. Thus, problems surrounding the representation of SDs and methods to calculate their evolution in models will remain relevant for a long time. Several challenges inherent in traditional Eulerian approaches, particularly for bin schemes, were discussed in section 3.1. Development over the past decade of the Lagrangian particle‐based approach provides an avenue to address many of these challenges, but there is also a fundamental conceptual advantage to Lagrangian particle‐based schemes: As the number of super‐particles approaches the number of actual particles, and the model resolution approaches DNS, these schemes converge exactly to particle‐by‐particle DNS calculations that provide the most complete approach currently possible for modeling a turbulent cloud volume. Thus, in principle, Lagrangian particle‐based schemes provide a rigorous path toward numerical convergence for cloud modeling. This is not true for traditional Eulerian‐based bin schemes, in which increasing the number of bins and the number of ice‐phase categories does not converge to particle‐by‐particle DNS. By their design, bin schemes will always require advection of continuous‐medium variables in both physical space and particle size/mass space. This is fundamentally different from discrete particles evolving and moving within a fluid, as occuring in real clouds.

In a more practical sense, Lagrangian particle‐based schemes offer several advantages compared to traditional approaches (see Grabowski et al., 2019 for more details). These address many of the specific challenges related to bin schemes discussed in section 3.1. First, by calculating microphysical evolution along Lagrangian trajectories of super‐particles within Eulerian dynamical models, numerical broadening from advection in physical space as well as in size/mass space from growth is avoided. Thus, calculations of particle growth/shrinkage from adiabatic expansion/compression are consistent with the model's vertical motion, including parameterized subgrid‐scale vertical motion that affects the Lagrangian trajectories. This means that SD evolution is governed by physical processes, as opposed to model numerics. Furthermore, Lagrangian particle‐based schemes avoid fundamental structural issues with the Smoluchowski equation solved numerically by bin schemes (see section 3.1). Although the super‐particle method assumes that particles are always well mixed, implying that collision‐coalescence is a Markovian stochastic process, it relaxes the no‐correlation assumption made in deriving the Smoluchowski equation. Thus, this method can provide the correct solution even when the well‐mixed volume is small (Dziekan & Pawlowska, 2017). The Lagrangian particle‐based approach calculates the underlying stochastic process and is therefore well posed to address a fundamental question concerning warm rain formation in ice‐free clouds, that is, whether it is more stochastic (e.g., Kostinski & Shaw, 2005; Wilkinson, 2016) or deterministic (Berry & Reinhardt, 1974a, 1974b, 1974c). Bin microphysics schemes solve the deterministic Smoluchowski equation and by design can only simulate the deterministic path to rain formation.

The Lagrangian particle‐based approach also allows straightforward incorporation of subgrid‐scale schemes to represent the multiscale nature of turbulent clouds. Following growth histories of a judiciously selected ensemble of individual particles provides a natural mechanism to include variations due to unresolved processes. These variations in growth histories may come from supersaturation fluctuations driven by unresolved vertical velocity variations (and lead to significant spectral broadening; Grabowski & Abade, 2017) or from entrainment and mixing (Abade et al., 2018; Hoffmann et al., 2019). In contrast, it is unclear how such effects might be incorporated into bin microphysics schemes in a physically based way.

**Figure 11:**
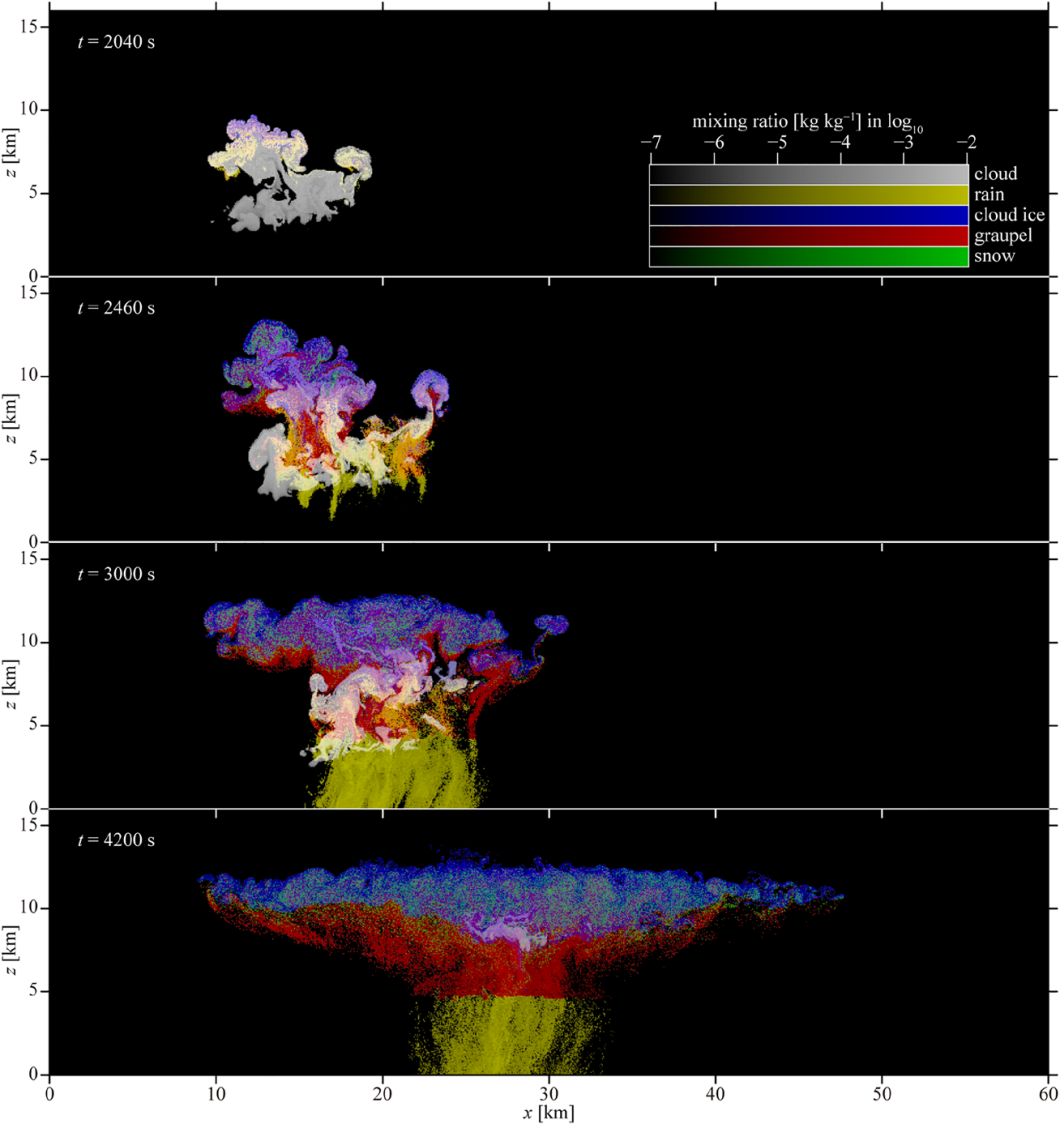
Vertical distributions of hydrometeor mass mixing ratios from two‐dimensional simulations of a single deep convective cloud using the liquid and ice Lagrangian particle‐based scheme of Shima et al. (2019). Results are shown at times of 2,040, 2,460, 3,000, and 4,200 s. The different hydrometeor categories illustrated are diagnosed by the attributes of the super‐particles: size, rimed mass fraction, and number of monomers. Adapted from Shima et al. (2019) (under the creative commons attribution 4.0 license).

The Lagrangian particle‐based approach helps to address the “curse of dimensionality” problem related to the computational challenge of predicting multiple properties in bin schemes, such as ice particle density and minor and major crystal axis lengths. Predicting how particle populations evolve as a function of various properties is straightforward in particle‐based schemes by adding more attributes to describe these properties beyond the standard ones of multiplicity, position in physical space, and wet and dry radius (see section 2). The particle‐based approach becomes more computationally efficient than the bin approach, with the latter representing each attribute as a separate dimension, when the number of attributes exceeds a number estimated to be between 2 and 4 (Shima et al., 2009). Representing ice particle properties in this way also provides a link to recently developed bulk microphysics schemes that are centered around prediction of ice particle properties (Harrington et al., 2013; Jensen et al., 2017; Morrison & Grabowski, 2008; Morrison & Milbrandt, 2015). Brdar and Seifert (2018) and Seifert et al. (2019) developed a particle‐based scheme (McSnow) with attributes of evolving ice particles that are similar to the predicted particle properties (P3) bulk scheme (Morrison & Milbrandt, 2015), and Shima et al. (2019) developed a scheme that evolves ice particles in a manner similar to the bulk scheme of Jensen et al. (2017). An example of results applying the scheme of Shima et al. (2019) in a deep convective cloud simulation is shown in Figure 11. The development of particle‐based schemes that include both liquid and ice is in its early stages, but we anticipate much more work being done in this area in the coming years.

Although Lagrangian particle‐based schemes have several conceptual and practical benefits, there are some outstanding challenges. Perhaps the most important is the lack of physical understanding of many microphysical processes, particularly those involving ice, which plagues all microphysics schemes. Particle‐based schemes are also computationally expensive as a fairly large number of super‐particles are needed in each grid volume in order to obtain accurate statistics. This problem can be exacerbated when super‐particles are advected from one grid to another, resulting in large statistical fluctuations (Grabowski et al., 2018). A rule of thumb is that roughly 50–200 super‐particles per grid box for two‐ or three‐dimensional cloud simulations are needed, depending in part on the number of predicted attributes (Andrejczuk et al., 2010; Arabas & Shima, 2013; Dziekan et al., 2019; Grabowski et al., 2018; Hoffmann et al., 2019; Jaruga & Pawlowska, 2018; Riechelmann et al., 2012; Shima et al., 2019; Sölch & Kärcher, 2010; Unterstrasser et al., 2017; Unterstrasser & Sölch, 2014). This leads to somewhat greater cost than typical bin scheme configurations, though the cost becomes comparable to or even less than bin schemes when they include more details such as the amount of solute dissolved in cloud drops, owing to the “curse of dimensionality” problem discussed above. The cost of particle‐based schemes can also be reduced by using simple methods for calculating the activation of cloud droplets from cloud condensation nuclei so that super‐particles only need to be carried within clouds and not in surrounding clear air (Grabowski et al., 2018). Even considering only the cloudy volume, for a similar number of super‐particles and bin variables per grid volume, the cost of a Lagrangian particle‐based scheme was found to only be 25% greater than bin microphysics (Grabowski, 2020).

In general, particle‐based methods are expected to produce larger variability among different realizations than should occur in nature, simply because microphysical behavior is represented by fewer particles than exist in real clouds. This leads to typically larger variability and spatial/temporal fluctuations in cloud quantities compared to simulations using bulk or bin microphysics (e.g., Grabowski, 2020). Relatedly, particle‐based methods are sensitive to how super‐particles are initialized, as it may be important to sample rare but important particles that may contribute significantly to precipitation formation (see Grabowski et al., 2019).

For particle‐based schemes, the treatment of the collision‐coalescence process is numerically challenging. Shima et al. (2009), Andrejczuk et al. (2010), Sölch and Kärcher (2010), and Riechelmann et al. (2012) proposed different numerical algorithms. Among those four schemes, the super‐droplet method (SDM) of Shima et al. (2009) provides a computationally efficient algorithm, if the super‐droplets are initialized appropriately. Unterstrasser et al. (2017) later developed the all‐or‐nothing algorithm (AON), based on the ideas of Shima et al. (2009) and Sölch and Kärcher (2010), and showed superior performance compared to other algorithms. However, Dziekan and Pawlowska (2017) found that the original SDM was more efficient than AON. Several other algorithms for collision‐coalescence have been proposed for related problems in other fields: the weighted flow algorithm (DeVille et al., 2011) for aerosol dynamics; the O'Rourke (1981) method and the no‐time counter method (Schmidt & Rutland, 2000) for spray combustion; and the methods from Ormel and Spaans (2008) and Johansen et al. (2012) for astrophysics. Li et al. (2017) confirmed that the performance of AON is better than Johansen et al. (2012), but direct comparison with other algorithms remains to be assessed. Furthermore, efficient particle‐based algorithms for spontaneous or collisional breakup processes, including rime splintering, have not yet been established.

Although Lagrangian particle‐based schemes represent an important advance, the computational cost limits their use to research modeling. For this reason, bulk schemes will continue to be used in operational weather and climate models for the foreseeable future. Moreover, evolving super‐particles along Lagrangian trajectories only makes sense in models that can explicitly represent cloud‐ and convective‐scale motions and thus is limited to smaller‐scale models (e.g., LES and convection‐permitting models). Thus, hierarchical strategies that leverage advances in smaller‐scale process models, especially Lagrangian particle‐based schemes given some of their advantages compared to bin schemes, should be sought to improve bulk schemes. Most obviously, cloud models employing particle‐based schemes could be used to formulate process rates for bulk schemes by direct fitting, such as autoconversion and accretion, as well as to improve representation of the SD. Such an approach was recently employed to develop a bulk scheme from a Lagrangian particle‐based scheme for liquid clouds (Noh et al., 2018). This is conceptually similar to earlier studies that used bin scheme results to formulate process rates in bulk schemes by fitting (e.g., Berry & Reinhardt, 1974d; Chen & Liu, 2004; Khairoutdinov & Kogan, 2000; Kogan, 2013; Kogan & Belochitski, 2012; Seifert, 2008). Less direct, but still hierarchical, output from particle‐based schemes could be used to inform parameter values statistically via Bayesian methods. Such an approach could also simultaneously leverage cloud and precipitation observations rigorously, as is described further in section 4.3.

### 4.2 Advances in Cloud and Precipitation Observations

Much of the uncertainty in current microphysics schemes stems from a lack of understanding of critical aspects of cloud physics and specific microphysical processes. Therefore, improving these knowledge gaps is a necessary step toward improving schemes and reducing model uncertainty. Below we highlight key areas of investment to advance process‐level microphysics knowledge from the standpoint of measurements.

#### 4.2.1 Laboratory Experimentation

Given that laboratory experiments provide the only practical means to quantify many individual microphysical process rates under controlled conditions, an obvious step is to invest more time and resources into laboratory work on microphysics. Though laboratory work appears to have declined since the 1970s and 1980s relative to other observational and modeling work in cloud physics (see section 3.3.1), recent laboratory studies have shown considerable success in advancing our basic knowledge and improving the way microphysical processes are represented in models. While we do not attempt to provide a comprehensive discussion of all such studies, we highlight a few examples below.

Work using cloud simulation chambers, like the AIDA chamber at Karlsruhe Institute of Technology, and with smaller‐scale continuous flow diffusion chambers or single‐droplet experiments, has enhanced our process understanding of primary and secondary ice formation (e.g., David et al., 2019; Lauber et al., 2018; Wagner et al., 2016; Yang et al., 2018) and has provided more reliable quantification of the ice nucleation efficiency of various aerosols than was previously possible (e.g., DeMott et al., 2018). Recent advances in process understanding of heterogeneous ice nucleation have also benefited from methods imported from surface physics, such as nonlinear optical spectroscopy and electron microscopy, and from molecular dynamics simulations (e.g., Chong et al., 2019; Slater et al., 2016). With these methods, it has been possible to identify the nature of ice nucleation active sites on feldspar and organic particles (Kiselev et al., 2017; Sosso et al., 2018) and to observe the first stages of ice growth on solid surfaces (Abdelmonem et al., 2015; Lovering et al., 2017). The transition from simple proxy materials to more complex atmospherically relevant surfaces is challenging but such work promises more insight in the coming years.

Research at Michigan Technological University has improved understanding of the influence of turbulence on droplet growth using the Pi chamber (Chandrakar et al., 2016, 2017; Chandrakar, Cantrell, Kostinski, & Shaw, 2018; Chandrakar, Cantrell, & Shaw, 2018; Chang et al., 2016; Desai et al., 2018). This work has led to considerable insight into the fundamental roles of turbulence and small‐scale variability on drop SDs and spectral broadening, including impacts on cloud‐aerosol interactions (Chandrakar et al., 2016, 2017; Chandrakar, Cantrell, Kostinski, & Shaw, 2018; Chandrakar, Cantrell, & Shaw, 2018). These researchers also recently corroborated the conditions suggested by Korolev and Mazin (2003) for the maintenance of mixed‐phase clouds (Desai et al., 2019). The group has developed a method to scale an LES model to the dimensions of the Pi chamber, thus providing a way to directly test microphysical models with data from the chamber (Thomas et al., 2019).

At the Meteorological Research Institute's cloud simulation chamber in Japan, various natural and artificial particles have been tested for ability to act as CCN and INP and used for climatological monitoring of background CCN/INP properties (Kuo et al., 2019). There are many new investments and developments of large expansion cloud chambers from the China Meteorological Administration branches at province and city levels in the last 5 years. For example, the Beijing Weather Modification Office as part of the Beijing Meteorological Services built a cylinder‐shaped expansion chamber in stainless steel with 14‐m height and 2.6‐m diameter in 2018.

**Figure 12:**
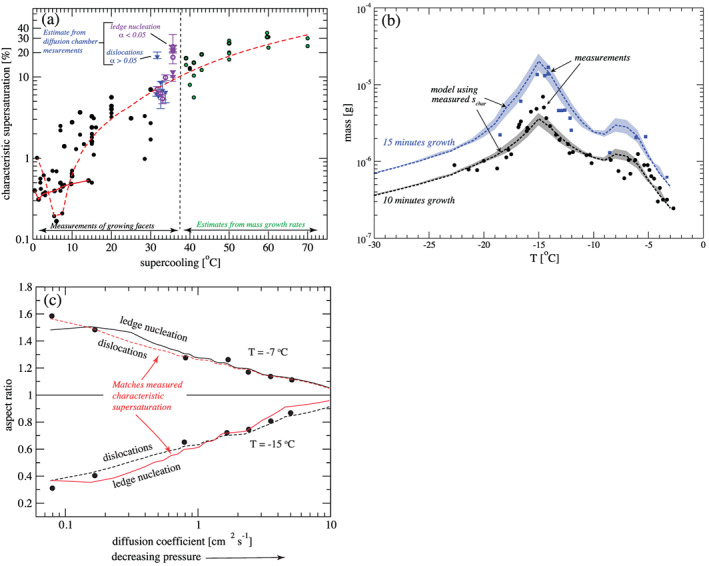
(a) Characteristic ice supersaturation *S*_*char*_ (*y*‐axis) as a function of temperature (*x*‐axis), obtained from laboratory measurements of faceted ice particle growth (black points) and approximated from mass growth measurements (green points). Values of *S*_*char*_ for two different ice growth mechanisms (dislocations, blue, and ledge nucleation, purple) are derived from diffusion chamber measurements (Pokrifka, 2018). Fits to the *S*_*char*_ data for the basal and prism facets are shown by the red dashed and solid lines, respectively (see Harrington et al., 2019 for details). (b) Comparison of modeled and measured single ice particle masses (*y*‐axis) after 10 min (black) and 15 min (blue) of diffusional growth at various temperatures (*x*‐axis). The model uses the fit values of *S*_*char*_ in (a) and assumes ledge nucleation growth. (c) Measured (dots) and modeled (lines) ice particle aspect ratio variation with pressure. Although it is possible to model the data with either growth mechanism, only dislocation growth at −7°C and ledge nucleation growth at −15°C also match independently measured *S*_*char*_ (see Harrington et al., 2019 for details). Figure adapted from Harrington et al. (2019) (©American Meteorological Society, used with permission).

Researchers at The Pennsylvania State University (PSU) have developed theories that link data taken from the vapor diffusion growth of faceted ice to the parameterizations used in cloud models (particularly habit evolving models such as Jensen et al., 2017). Laboratory measurements in general, and studies at PSU in particular, provide values for the ice crystal surface parameters needed for these growth models (Harrington et al., 2019). For example, Figure 12 indicates how laboratory measurements have been used to characterize the role of surface attachment kinetics on ice vapor depositional growth and the development of the primary particle habits (see section 3.2.3), which provide the foundation for physically based growth parameterizations (Harrington et al., 2013; Zhang & Harrington, 2014, 2015). The effects of surface kinetics on the growth of ice depend fundamentally on characteristic (or critical) ice supersaturations *S*_char_ for the basal and prism facets that strongly depend on temperature (Figure 12a). Curve fits to the measured values of *S*_char_ are used as input for the faceted growth model. Comparison of the growth model solutions to independent laboratory data indicates that the model can accurately reproduce the measured particle masses (Figure 12b), as well as aspect ratios and fall speeds (Harrington et al., 2019), after 10 and 15 min of growth over a wide range of temperatures.

While these laboratory measurements are useful for corroborating the theories used to develop parameterizations, they also provide important information on the limitations of current methods. For instance, Figure 12b indicates that the model can reproduce the mass evolution of larger ice crystals but only if the crystals grow by the nucleation of ledges on their surface. However, the model can fit pressure‐dependent data of small ice crystals (Figure 12c) if growth is controlled by either ledge nucleation or by permanent dislocations in the crystal lattice. Only growth by dislocations at −7°C and step nucleation at −15°C reproduce the measured aspect ratios with measured values of *S*_char_, indicating that at −7°C, growth may have been controlled by dislocations. This latter result is at odds with the results in Figure 12b, which require ledge nucleation growth. It is therefore likely that some processes are missing from the current model and theory. Similarly, recent laboratory measurements at PSU provide evidence that the growth rates may be influenced by the lateral spreading of facets, for which no current model exists (Pokrifka, 2018). Moreover, Pokrifka (2018) showed that the mass growth rates may depend on the ice nucleation mechanism.

To build on these efforts may require a sustained reinvestment in laboratory experimentation if indeed it has generally declined over the last 30 years, as we suspect (see section 3.3.1). Because most of the authors of this paper are not experimentalists, we do not feel particularly qualified to provide specific paths forward on this aspect. However, we emphasize the need for a discussion within the broader cloud physics community on whether a broad decline in laboratory research has indeed occurred and, if so, identifying the forces driving this decline and proposing possible solutions to reverse the trend. We do provide some possible paths forward at the intersection of parameterization development and laboratory work. Better and longer‐term collaborations among numerical model developers and laboratory scientists would likely accelerate the improvement of microphysics schemes. While this point is often stated, these sorts of collaborations are rarely achieved in practice. Incentivizing long‐term collaborations of this sort is one way that this could be achieved, though it would require assent from funding agencies. Longer funding cycles for such collaborations would also be useful, since a typical 3year grant cycle is generally not long enough for a fruitful collaboration to develop among laboratory scientists and modelers who speak fundamentally different languages. It may also be useful to adopt practices that have been successful in some locations. For example, laboratory cloud research in many European countries has received steady support over the past decades at nonuniversity research institutions, which often benefit from a relatively stable base funding (e.g., CNRS in France, Helmholtz Centres, and Leibniz Institutes in Germany). These institutions have been able to maintain experimental facilities, such as large simulation chambers, over a long period of time. Keys to this success are long‐term commitment by the funders, international collaboration, and joint activities such as the transnational access to 17 atmospheric simulation chambers within the EU‐funded consortium EUROCHAMP, the European research infrastructure ACTRIS (both including calibration centers for measurement instruments), and the Cosmics Leaving OUtdoor Droplets (CLOUD) experiment with many international partners at CERN. Similarly, the Meteorological Research Institute (MRI) as part of the Japan Meteorological Agency has a cold environment simulator facility including cold rooms and a cloud simulation chamber and a large wind tunnel facility for laboratory experiments. These successes suggest that it may be worth considering investing in laboratory research at national centers, where it may be more likely to maintain stable base funding. Other paths forward are possible, of course, and should be part of a broader discussion in our community regarding the current health and possible future of laboratory research in cloud physics.

#### 4.2.2 Observations of Natural Clouds and Precipitation

Historically, the development of cloud microphysical instrumentation has been done by small private companies or individual researchers affiliated with universities or government institutions. Despite the support of these initiatives by funding programs at the national level, instrumentation development has often been driven by profitability rather than by research needs. This dynamic cuts away at the development of more complex instrumentation that has a higher cost at the research and development stage. Thus, it is difficult to support the development of new airborne or ground‐based probes, which requires years of effort and associated funding. As a consequence, legacy probes that are many decades old continue to be deployed routinely despite well‐known limitations of a fundamental nature: for example, saturation of signal prevents measuring the highest droplet number concentrations or mass contents and lack of absolute calibration tools results in comparing one uncalibrated probe to another in order to estimate uncertainty (see McFarquhar et al., 2017 for a detailed discussion of limitations related to SD measurements). In some ways this echoes the challenges of funding and maintaining cloud physics laboratory work. Longer‐term programs with sustained funding for the development of complex airborne in situ instrumentation, similar to the development of satellite‐based instrumentation, may be needed to address this problem. Again, this may be suited for nonuniversity national centers that may be able to better maintain stable base funding.

Despite these challenges, new probes using various technologies are being built; outside of Europe, this is often from funds that are cobbled together from multiple limited sources, such as brief support from agencies concerned with civilian aircraft safety in recent years, one‐time research development funds from national laboratories, and even personal scientist investment. Recent advances in airborne probes include the development of holographic probes that quantify droplet clustering and ice crystal optical properties (e.g., Abdelmonem et al., 2016; Fugal & Shaw, 2009), a phased‐Doppler interferometer for more reliable measurement of droplet SDs (Chuang et al., 2008), and an isokinetic probe adequate to sample large ice mixing ratios (Davison et al., 2009; Strapp et al., 2016). Notable new ground‐based probes include multicamera instruments (e.g., the Particle Flux Analytics Multi‐Angle Snowflake Camera or MASC) capable of advancing systematic characterization of frozen hydrometeor shape (e.g., Garrett et al., 2012; Schönhuber et al., 2008), which is a fundamental aspect of ice microphysics schemes that underlies many process rate uncertainties (e.g., sedimentation, growth rate, aggregation propensity, etc.). Recent advances in data processing have also led to significant improvements in the accuracy of in situ measurements (e.g., Baumgardner et al., 2017; Korolev et al., 2017; McFarquhar et al., 2017). However, measuring small‐scale variability of a hydrometeor population remains a persistent problem. Particle probes in current use generally employ a single‐particle method for measuring particle SDs. The development of single‐particle probes seems to have reached saturation, and further refinement is neither expected to significantly improve sampling statistics nor reduce the spatial averaging scale required to get robust results. The introduction of holographic particle probes and their further development may significantly improve assessment of particle SDs on a small scale. However, their sampled volume is still orders of magnitude smaller than that required for rigorous validation of remote sensing products and comparisons with cloud simulations. Resolution of this problem, as well as challenges in measuring other quantities like electrical charge of hydrometeors and in‐cloud supersaturation, may require the development instrumentation based on completely different physical principles. We also point to the use of unmanned aerial vehicles (UAVs) for cloud microphysical observations (e.g., Woods et al., 2018) related to the need to as a developing area with considerable potential, but there are several challenges in miniaturize sensors, limited payload, and power restrictions. Thus, at present, UAVs cannot compete with the quality and completeness of microphysical data collected from conventional airborne research platforms.

Besides instrument and platform advances, we suggest field campaign and measurement sampling strategies to obtain observational data sets that are maximally effective for scheme evaluation and constraint, especially in view of the significant temporal and spatial variability that occurs in natural clouds and precipitation. The substantial cost of airborne flight campaigns already places high standards on choice of region for data collection, flight patterns, and number of sorties in order to address project goals. Random sampling that was typical for early studies of cloud microphysics in the 1950s–1970s is not currently used unless specifically required by project goals (e.g., aviation safety campaigns). Modern field campaigns instead seek to optimize data collection by various means. For instance, a quasi‐Lagrangian sampling strategy has been used for the objectives of improving schemes for ice vapor growth (e.g., Field, 1999) and establishing ice initiation mechanisms in wave clouds (e.g., Field et al., 2012). Another strategy is to focus data collection and model observation comparisons on well‐defined regions where microphysical properties can be robustly sampled because they are varying only slowly spatiotemporally, as in widespread convective ice outflow over stratiform rain (e.g., Fridlind et al., 2017). In the case of shallower stratiform cloud systems with significant heterogeneity, sample robustness can be improved by selecting quasi steady‐state cloud fields that can be repeatedly profiled over more than one flight (e.g., McFarquhar et al., 2007). When flights sampling similar cloud systems can be aggregated, basic process occurrence can be systematically investigated in the greatest detail (e.g., Rangno & Hobbs, 2001), including individual ice crystal morphologies and hydrometeor SD features that bear direct evidence of ice formation, vapor growth, riming, and aggregation processes.

As mentioned in section 3.3.3, ground‐ and aircraft‐based cloud and precipitation radars have been increasingly used to characterize hydrometeor properties and to provide insights into microphysics. These platforms provide substantial spatial coverage (unlike in situ observations) of microphysically relevant observations, are capable of resolving the full depth of cloud and precipitation features (unlike most satellite observations), and feature relatively fast temporal resolution (unlike polar‐orbiting satellites). Although direct constraint of models using these data is challenging, progress can be made by considering specific outstanding needs of such efforts. For studies of rain, polarimetric radar variables provide information related to the sixth moment of the SD (via reflectivity), the 4th to 5th moment (specific differential phase), and higher‐order moments (differential reflectivity). Vertically profiling radars, meanwhile, are sometimes capable of separating cloud and precipitation modes and provide information on hydrometeor fall speed. Both may be needed to disentangle the combined effects of evaporation, collisional coalescence, and breakup. For ice hydrometeors, polarimetric quantities give information related to aspect ratio, particle density, and concentration, but some particle properties may still remain unconstrained owing to the complexities of solid‐phase hydrometeor habits and particle orientations. Here, the addition of fall speed information from vertically pointing radars may reduce some of these uncertainties. While the predominantly qualitative use thus far of polarimetric radar observations for process‐level microphysical understanding suggests challenges in how these data can be used to constrain schemes, recent work has suggested that such observations can be used to extract *quantitative* process‐level microphysical information; this is discussed in more detail in section 4.3. Further progress in observational constraint of schemes using polarimetric radar and other remote‐sensing data may require finding approaches to maximally leverage their information content while also considering and quantifying the effects of both observational and model uncertainties.

The occurrence of multiple interacting microphysical processes and the additional complexity of ice particle habit and density strongly argue for the use of both polarimetric and vertically pointing radars in conjunction with detailed surface or in situ observations to allow for comprehensive constraint of as many uncertain hydrometeor properties as possible. In some cases, such as for rain in the absence of cloud, lidar backscatter may provide important and unique constraint of the second moment of the rain SD, and Doppler lidar can provide the flux of the second moment (e.g., O’Connor et al., 2005; Westbrook et al., 2010). In other cases, such as shallow liquid clouds, polarimetric radars will provide little benefit beyond quality control of clutter and biological scatterers, though Doppler spectral information can be useful (Luke & Kollias, 2013; Rémillard et al., 2017).

Scan strategies should also be tailored to the weather conditions present and the processes that researchers are interested in constraining. For example, performing quasi‐vertical profiles may be the best way to extract microphysical information from polarimetric radars for spatially homogeneous features such as broad regions of stratiform precipitation (Ryzhkov et al., 2016) in conjunction with vertically pointing radars. Convection, on the other hand, may benefit from detailed scans of rapidly evolving updrafts, ideally from platforms designed for high spatial and temporal resolution (e.g., Fridlind et al., 2019). Here, profiling radars may provide long‐term statistics of updrafts (e.g., D. Wang et al., 2019) but are not capable of the spatial coverage needed to capture fully the dynamic, thermodynamic, and microphysical properties of convective features. In this context, phased array radars (e.g., Fulton et al., 2017; Zrnic et al., 2007) and imaging radars (e.g., Isom et al., 2013) promise to advance understanding microphysics dramatically for convective regimes by providing an order of magnitude better temporal resolution than traditional radars. For this sort of rapidly evolving weather, it is unlikely that quantitative process retrievals will be straightforward, for example, there still exists a fundamental lack of understanding of small‐scale updraft and downdraft dynamical features, such as entrainment (e.g., de Rooy et al., 2013). Even with improved instrumentation and deployment strategies, rigorous evaluation and constraint of microphysics schemes should take these dynamical aspects and uncertainties into account. Though these advanced observing systems have yet to be used in microphysical studies, and despite the challenges associated with such data sets for microphysical retrievals, these systems hold considerable promise.

Satellites will remain a critical component for evaluating and constraining microphysics schemes owing to the global coverage they provide, allowing characterization of cloud and precipitation features that are otherwise very seldom and sparsely sampled (e.g., over oceans). The difficulty in using these observations directly to inform microphysics schemes was remarked on in section 3.3; this begs the question of how future satellite observations can be designed so as to better serve the needs of scheme development. We emphasize that in order to constrain microphysics schemes robustly, next‐generation missions will need to advance retrievals of *microphysical variables*; bulk quantities alone are of much lesser value. Fortunately, some new technologies can support that. For example, future multiangle polarimetry of sufficient viewing angle resolution (i.e., on the order of 2 degrees; Miller et al., 2018) can provide pixel‐level retrievals of droplet sizes at cloud top for small, inhomogeneous or mixed‐phase clouds for which heritage bispectral approaches generally fail (Alexandrov et al., 2012; Miller et al., 2018). Furthermore, such multiangle polarimetry observations allow inference of the SD width or general SD shape (Alexandrov et al., 2012). Where satellite measurements generally struggle to determine robustly the thermodynamic phase of clouds with tops between the homogeneous freezing and melting temperatures, polarimetric detection of a cloudbow is a virtually unambiguous indication of liquid drops at the tops of clouds (Riedi et al., 2010, van Diedenhoven, Cairns, et al., 2012). For ice‐topped clouds, multiangle polarimetry allows inference of crystal shape (Baum et al., 2011; van Diedenhoven, Fridlind, et al., 2012; van Diedenhoven, 2018; van Diedenhoven et al., 2020), which may be especially valuable for evaluation of microphysical models predicting ice shape characteristics (e.g., Harrington et al., 2013; Hashino & Tripoli, 2007; Jensen et al., 2017). Furthermore, the inferred ice shape constrains the ice optical model used for retrievals of ice cloud optical thickness and effective radius from shortwave infrared measurements (van Diedenhoven et al., 2014; van Diedenhoven et al., 2020), reducing uncertainties. Combining such pixel‐level polarimetric retrievals of cloud SDs with cloud‐top extinction measurements from a lidar with sufficient vertical resolution will allow a physically based retrieval of droplet number concentrations with accuracies well beyond current capabilities (Grosvenor et al., 2018). Currently, only *airborne* polarimeters exist with angular resolution on a pixel level sufficient to resolve variations in the cloudbow for SD retrievals, but the Hyper Angular Research Polarimeter (HARP‐2) planned on the US National Aeronautics and Space Administration's Plankton, Aerosol, Cloud, ocean Ecosystem (PACE; Werdell et al., 2019) satellite mission could provide the first such spaceborne measurements in the near future. We note, however, that such measurements only provide information on microphysical quantities at or near cloud top, whereas vertically resolved information throughout the depth of the whole cloud layer is particularly useful for scheme evaluation. One promising approach is to synergistically combine observations using active radar, which resolves a greater depth of cloud columns and senses the sixth moment of the SD, with passive measurements at microwave, infrared, or shortwave wavelengths, which are sensitive to lower moments of the SD but generally cannot resolve information vertically (e.g., Leinonen et al., 2016; C. Wang et al., 2019; Saito et al., 2019; Xu et al., 2019).

There is also the promise of new satellite technologies that have the potential to improve the spatial and temporal resolutions of observations and as such to link more directly to rapidly evolving cloud processes. Recent advances in geostationary satellite instruments with high resolution and extended spectral range, such as the Advanced Himawari Imager (Bessho et al., 2016) and the Advanced Baseline Imager (Schmit et al., 2005), have unexploited potential to provide insight through their high temporal resolution over the diurnal cycle. Recent experiments that assimilate such data in convection permitting models show promise for the constraint of cloud vertical and horizontal extent, along with synoptic and mesoscale dynamics and the thermodynamic environment (Minamide & Zhang, 2018). Furthermore, geostationary passive microwave sensors could provide relatively rapid (approximately 10 min) views of precipitation features over a wide swath of the globe. In addition, miniaturization has enabled the launch of constellations of small satellites, which potentially enable rapid sampling of cloud features. When coupled with innovations in adaptive sampling and signal processing, small satellite constellations have the potential to provide measurements of cloud processes as they evolve in time. For example, convoys of spaceborne small‐sat radars have the potential to provide unique observations of the time evolution of microphysical variables in the interior of clouds, as well as the vertical motion of hydrometeors (Haddad et al., 2018; Stephens et al., 2019).

We recommend that design studies of future satellite missions consider the integrated information content of various combinations of passive and active observations to better constrain uncertainties in microphysics schemes. Especially well suited for such studies and retrieval algorithms are optimal estimation and related Bayesian techniques, as these methods are capable of combining the information content of different types of measurements and provide robust uncertainty estimates. Other popular advanced approaches to infer microphysical quantities from satellite data such as neural networks (e.g., Di Noia et al., 2019; Holl et al., 2014) may be less suitable for constraining microphysics schemes as uncertainties of their outcomes are generally not quantified.

More generally, there is an outstanding need to quantify robustly the uncertainty of *any* observation used to inform microphysics schemes. In the case of Bayesian methods as discussed in section 4.3, observational uncertainty plays a large role in determining how much information can be gained from observations of interest. Overestimation of uncertainty can lead to reduced information content of observational data, reducing their effectiveness. Conversely, an underestimation of uncertainty may cause unrealistic noisy fluctuations in observed quantities to be interpreted as meaningful information. Although theoretical estimates of observational uncertainty are well known for most observing platforms, few studies have attempted to confirm these estimates through analyzing the observed quantities themselves. In all cases, field campaigns and intensive observational experimental design must occur in close collaboration with microphysical modelers to yield observational data sets targeted to gaps in knowledge and process‐level uncertainties. One promising avenue to facilitate such collaboration is to *combine* both retrieval and forward simulation approaches in close model observation comparisons, with an emphasis on basic cloud structural context (e.g., Fridlind et al., 2019). Such an approach seeks to build on the past success of the Steiner et al. (1995) algorithm, for example, wherein the structure of tropical rain systems is analyzed via the horizontal pattern of observed or simulated reflectivity at some distance *below* the melting level (where all hydrometeors are liquid phase in stratiform regions). This would avoid the much greater biases of most microphysics schemes in forward‐simulating ice‐phase reflectivity for a variety of reasons that pertain to the complexity of ice particles.

### 4.3 Leveraging Observations to Advance Microphysics Schemes Systematically

Regardless of the particular scheme or modeling application, the rigorous incorporation of information from in situ and remote‐sensing observations of natural clouds and precipitation to improve schemes is challenging, particularly because it is generally very difficult to quantify individual microphysical process rates from these observations. Up to now, natural cloud and precipitation observations have been mainly used to constrain process rates in schemes via comparison with model output and tuning, often in ad hoc ways (see section 3.4).

We argue that new methods are needed to make better use of these observations, especially given the wealth of cloud and precipitation observations now available. In general, across all model types and for all cloud and precipitation observations, the goal is to improve uncertain schemes *optimally* by comparison with (somewhat) uncertain observations; this presents an inverse problem. Put another way, we wish to update the probability of a microphysics scheme and its parameters based on new observational information. This echoes Bayes' theorem from the field of statistics:

(2) $$P\left(x|y,g\right)\;=\;\frac{P\left(y|x,g\right)P\left(x|g\right)}{P\left(y|g\right)},$$

where *g* ≡ *g*(**x**) is a model that takes in a vector **x** of parameter values and **y** is a vector of observational data. In essence, Bayes' relationship formalizes the concept of updating knowledge using probability distributions. Starting with a *prior* distribution that represents the current state of knowledge of parameter values **x**, one obtains data (or observations, **y**) and updates the prior distribution from these data. The result is the *posterior distribution* on the left‐hand‐side of Equation 2. Note that the right‐hand‐side of Equation 2 can be further decomposed to account separately for uncertainties associated with the observations themselves and the forward operator that converts model output to an observable quantity (Posselt et al., 2015). The power of Bayes relationship comes from the fact that the posterior distribution becomes the new current state of knowledge (in essence, the new prior distribution) and can be further updated with new information (observations) as it is collected. This is similar to traditional data assimilation but with an important difference: whereas data assimilation combines a model‐based estimate of the state of a system with observations to produce an optimal estimate of a set of state variables, here we are considering how observations can be used to constrain uncertainty in the model itself.

If we take the perspective that microphysics schemes have sources of uncertainty that can be expressed as a probability distribution, then it is natural to constrain microphysics schemes observationally using Bayesian methods. This provides a framework within which new information naturally builds upon previous results (e.g., as new observations become available). Another advantage to using Bayesian methods is that they return a quantitative estimate of uncertainty, which is a critical aspect but relatively unexplored for microphysics schemes.

A Bayesian perspective on the development of microphysics schemes presents a very different philosophy compared with previous efforts that have taken a purely “physical” approach to parameterizing microphysics. Schemes have incorporated available cloud physics knowledge, but because it is limited, they have also traditionally relied in large part on heuristics and/or ad hoc tuning to produce results that are consistent with observations (Figure 8). Though not yet adopted widely for developing physical parameterizations in atmospheric models, Bayesian methods have been used to estimate parameters and quantify uncertainty in land surface and hydrology models (e.g., Beven et al., 2007; Franks & Beven, 1997; Kavetski et al., 2006; Knorr & Kattge, 2005; Raoult et al., 2016; Shi et al., 2015; Smith & Marshall, 2008; Thiemann et al., 2001). Land surface modeling faces a similar challenge to microphysics—namely, the extreme complexity of interacting, poorly understood chemical, physical, and biological processes—and from this standpoint, it is perhaps not surprising that a recent land surface model intercomparison study showed superior performance of purely statistical schemes compared to physically based models in benchmarking tests (Best et al., 2015). Despite this commonality with land surface modeling, it is evident that microphysics scheme developers have not yet embraced statistical methods as widely as land surface and hydrology modelers. Nonetheless, a handful of studies over the past decade have used Bayesian methods to investigate microphysics scheme uncertainty (e.g., Posselt, 2016; Posselt et al., 2019; Posselt & Vukicevic, 2010; van Lier‐Walqui et al., 2012). However, this has been done in a post hoc framework by estimating posterior distributions of uncertain model parameters in *existing* microphysics schemes using Bayesian inference (specifically Markov chain Monte Carlo methods or MCMC). These studies demonstrated how rigorous statistical methods can be applied to estimate parameter values and quantify uncertainty, potentially opening the door to more rigorous observational constraint of schemes. However, there are several challenges as we discussed in section 3.4, perhaps most notably that it is difficult or practically impossible to quantify structural uncertainty based on the design of current schemes.

**Figure 13:**
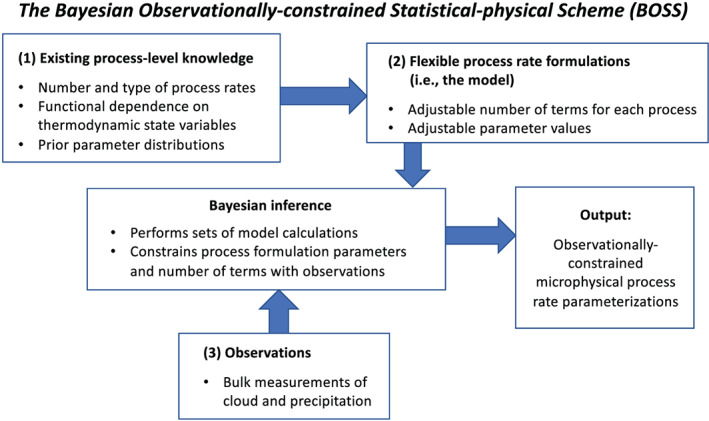
Schematic diagram of BOSS. Prior microphysical knowledge in (1) informs the process rate formulations in (2). It may also include information on the prior parameter distributions (e.g., ranges of possible parameter values). The process rate formulations in (2) are general and flexible with adjustable number of terms and parameter values. The process rate formulations comprising the model are constrained using Bayesian inference with observations of bulk cloud and precipitation properties (e.g., radar polarimetric variables) in (3). The output is a set of observationally constrained process rate parameterizations. Adapted from Morrison et al. (2020) (©American Meteorological Society, used with permission).

**Figure 14:**
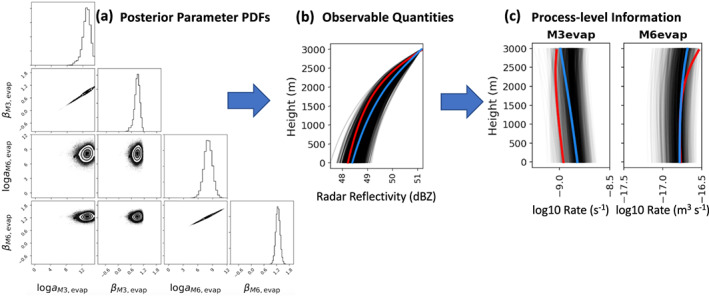
Results from BOSS parameter estimation experiments constrained by synthetic “observations” of polarimetric radar quantities (reflectivity at horizontal polarization, differential reflectivity, and specific differential phase) generated from a bin microphysics scheme at 16 vertical levels between 0 and 3 km height and surface rain fluxes. (a) Posterior parameter probability density functions (PDFs) of four parameters describing rain evaporation rate in BOSS with one‐dimensional marginal PDFs along the diagonal and two‐dimensional joint marginal PDFs below the diagonal with shading showing regions of higher probability. (b) Vertical profiles of radar reflectivity from one example case. (c) Vertical profiles of rain evaporation rate of the third “M3” and sixth “M6” SD moments (proportional to bulk mass and radar reflectivity factor, respectively) from the same example case. In (b), the blue line shows the reflectivity from BOSS using the maximum a posteriori parameter values with the moment‐based instrument simulator forward operator from Kumjian et al. (2019); the constraining reflectivity “observations” directly calculated from the bin scheme are shown in red. In (c), the blue lines show rain evaporation rate profiles calculated using the BOSS maximum a posteriori parameter values; the red lines show the evaporation rates from the bin scheme used to generate the synthetic “observations,” which were not used for constraining BOSS. In (b) and (c), black lines indicate individual BOSS simulations calculated by sampling the posterior parameter PDFs in (a). A total of 40 cases with varying environmental conditions were used to constrain BOSS. Note that all rain microphysical processes (evaporation, collision‐coalescence, breakup, and sedimentation) were constrained simultaneously, but only parameter distributions and rates for evaporation are shown for brevity. Other details of the modeling methodology follow van Lier‐Walqui et al. (2020).

An alternative approach is to develop microphysics schemes following the principle of Bayes' theorem at the outset. This idea has not yet been adopted into models but was recently explored as a proof of concept by Morrison et al. (2020) and van Lier‐Walqui et al. (2020). In these studies, the basic approach (called the Bayesian Observationally Constrained Statistical‐physical Scheme or BOSS) is to center the bulk scheme on a set of flexibly designed microphysical process rates. No explicit SD functional form is assumed, similar to some earlier studies (Kogan & Belochitski, 2012; Szyrmer et al., 2005). Instead, relationships among SD moments are characterized generally following the SD normalization method of Morrison et al. (2019). Individual microphysical process rates are formulated as a function of the set of predicted bulk moments via generalized power expressions—essentially, a sum of power law terms—with an adjustable number of terms and parameter values. Complexity can be increased systematically by adding more terms in the generalized power expressions or by adding more predicted bulk moments, allowing for a rigorous exploration of structural as well as parametric uncertainty. Observational constraint is achieved via Bayesian inference and calculation of posterior distributions of the scheme parameters. A schematic of the approach is shown in Figure 13.

van Lier‐Walqui et al. (2020) demonstrated this approach using MCMC, with constraint by synthetic “observations” (output generated by a different model) of rain SD moment profiles for a one‐dimensional steady‐state rainshaft. Here, we have extended van Lier‐Walqui et al. (2020) by using a bin microphysics scheme to generate synthetic polarimetric radar “observations” as the constraining data (Figure 14). Consistent with van Lier‐Walqui et al. (2020), parameters and individual process rates can be reasonably well constrained with few a priori assumptions. The degree of observational constraint is evident by the narrowness of the one‐dimensional and two‐dimensional marginal posterior parameter distributions (Figure 14a), as the prior parameter distributions were specified to be uniform over the range of values shown. The posterior distributions of rain evaporation rates from BOSS, which were not directly constrained, match well with the evaporation rates calculated from the bin scheme used to generate the synthetic observations (Figure 14c).

There are several potential benefits of a Bayesian approach to microphysics scheme development. If the scheme within the Bayesian framework is physically based, such as BOSS, it could be used to map cloud and precipitation observations to physical quantities that cannot be directly measured, process rates in particular. This is similar to the idea of using remote‐sensing observations to “fingerprint” microphysical processes (e.g., Kumjian & Prat, 2014) but using Bayesian methods. There are potential advantages compared to traditional process rate retrievals, particularly the rigorous quantification of uncertainty. There are also many other potential applications that arise from the ability to account for both structural and parametric uncertainty in microphysics, particularly in the context of ensemble forecasting and data assimilation. For example, the posterior parameter PDFs could be sampled to provide a physically based method of generating ensembles for weather and climate modeling with varying parameter values. Similarly, these PDFs could be stochastically sampled and varied in time and space to provide a physically based stochastic parameterization, as opposed to the ad hoc parameter perturbations that are employed in current stochastic physics parameterization approaches (e.g., Jankov et al., 2017). Moreover, the systematic development of process rates centered around analytic functions informed by observations would make deriving adjoints for variational data assimilation trivial.

Before going further, we comment on the philosophical underpinnings of Bayesian inference as a scientific tool for microphysics scheme development. In the traditional application of Bayesian inference, scientific progress is achieved by gathering data, using these data to calculate posterior probabilities, and repeating the process as new data becomes available. This has often been tied to inductive learning—understanding the general from the particulars. Applied as such, the idea is to use Bayesian inference to arrive at scientific “truths” through successive Bayesian updating as new data are obtained. This is by nature reductionist; fewer and fewer options are available to describe the system as increasing amounts of data are gathered. As a counterpoint, classical statistics based on frequentist inference, for example, the well‐known *p* test and other methods of hypothesis testing, do not attempt to converge on “truth” but only to reject hypotheses that are inconsistent with data. The viewpoint of Bayesian inference as an instrument for inductive learning has come under criticism (e.g., Gelman & Shaliz, 2013); these critiques are relevant in the context of using Bayesian inference as a framework for microphysics scheme development. Taking a model blindly as the framework for arriving at “truth,” with parameters that are estimated from data, can result in overconfidence in the model. This is probably less of a concern when modeling physical systems, as opposed to social or economic ones, but is important to keep in mind, especially for exceedingly complex physical systems like microphysics. Essentially, the problem is one of *structural* uncertainty; that is, uncertainty in the underlying structure of the model, as opposed to uncertainty in the parameters (see section 3.4). Using Bayesian inference as a tool for inductive learning is based on the assumption, usually not explicitly stated, that uncertainty resides entirely within the set of model parameters being estimated. If there is structural uncertainty, then Bayesian inference may not capture the true uncertainty in the model. One way to address structural uncertainty is to use multiple competing models and to perform Bayesian model selection or model averaging (e.g., Hoge et al., 2018; Sambridge et al., 2013). However, this does not fully address the problem; if the true equation set is unknown, then it is impossible to know if the set of discrete models being tested actually spans “truth” within a multischeme or multimodel framework.

While there is no avoiding structural uncertainty for systems in which the governing equation set is unknown, such as for microphysics, there are techniques that can help make the problem more tractable. Model checking is a valuable tool for verifying the soundness of model formulations (e.g., Box, 1980; Gelman & Shaliz, 2013; Jaynes, 2003; Morris, 1986). This usually involves verifying the model against data; for example, checking the quality of model performance against independent data sets not used during the inference step. In the context of microphysics, this could involve testing the model for cases or cloud regimes different from those used for learning or against data sets from different types of observations (i.e., different instruments or platforms). Through the process of inference (learning) and independent testing, Bayesian methods can find sources of model error that may otherwise be difficult to uncover. Likewise, using Bayesian inference with different combinations of observational data from various sources, together with rigorous estimation of information content from these data, could be a guide to help focus observational efforts. These are not typical uses applying Bayesian statistics to modeling but seem especially relevant for the problem of observationally constraining microphysics schemes.

**Figure 15:**
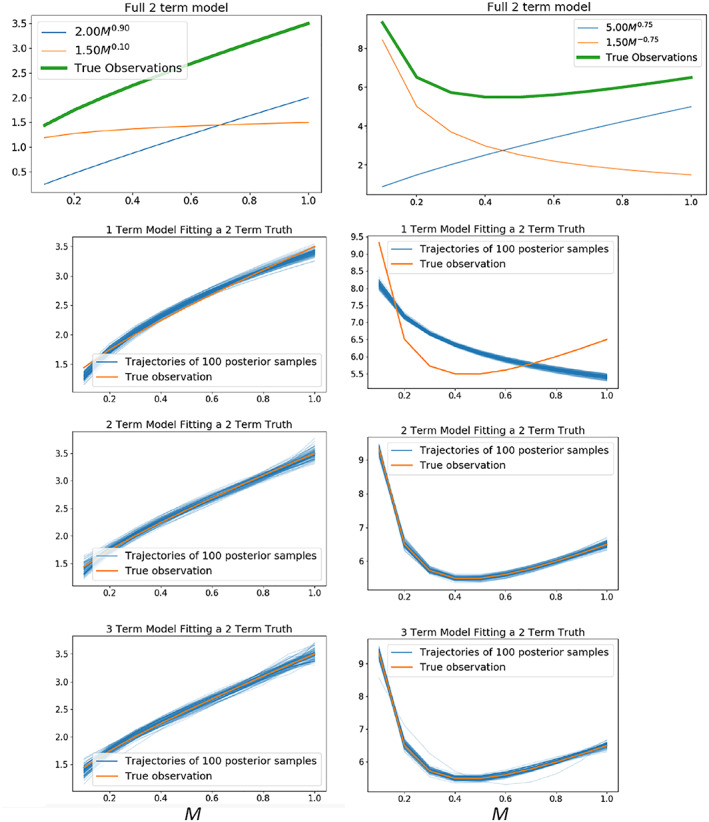
Illustration of model *expansion* and *parsimony* in very simple idealized “known truth” experiments. A model consisting of the sum of an adjustable number *N* of power law terms (i.e, the generalized power expression: $\sum_{n=1}^{N}a_{n}M^{b_{n}}$, where *M* is the independent variable and *a*_*n*_ and *b*_*n*_ are adjustable parameters) is fit to the “true observations” using MCMC for two different cases, where data for the “true observations” are generated by a two‐term power law sum. In the left column the “truth” is monotonic and given by 2*M* ^0.9^ + 1.5 *M* ^0.1^ (top left, green line); in the right column it is non‐monotonic and given by 5*M* ^0.75^ + 1.5 *M* ^‐0.75^ (top right, green line). The axes have arbitrary units. For the monotonic case on the left, the single‐term model (*N* = 1) can reasonably reproduce the “truth,” while for the non‐monotonic case on the right, two terms (*N* = 2) are needed in the model for a reasonable fit. In both cases the three‐term model with *N* = 3 (bottom panels) does not improve the fit compared with the two‐term model, which is expected as the “truth” consists of two terms. Thus, here parsimony is achieved by the model using *N* = 1 for monotonic case and *N* = 2 for the non‐monotonic case. Adapted from van Lier‐Walqui et al. (2020) (©American Meteorological Society, used with permission).

The second approach is to tackle structural uncertainty head on, not via Bayesian model selection with ad hoc multimodel ensembles but through careful construction of models that can rigorously probe structural uncertainty. This approach falls into the category of model *expansion*, whereby model richness and complexity are added and tested systematically. In this way, changes in model structure are an attempt to span structural uncertainty. Moreover, by building up and testing scheme complexity systematically, as opposed to the typical ad hoc addition of complexity in schemes, *parsimony* can itself be informed by data. In other words, the model only has to be as complex as needed to achieve consistency with the data and indeed only should be based on the principle of parsimony (in the absence of theoretical knowledge guiding how complex the model should be). If the system has certain known conditions such as smoothness and positive definiteness, then model expansion can be done with mathematical rigor even if the “true” equation set is unknown. The simplest example is a hypothetical system that is known a priori, say from physical principles, to consist of a set of smooth functional relationships between a set of independent and dependent variables over a finite range of the dependent variables. From the Weierstrass approximation theorem, all functional relationships that are smooth can be approximated over a closed interval as closely as desired by polynomial functions. Thus, in this example, structural uncertainty can be investigated rigorously within a Bayesian framework by testing models comprising polynomial functions that have systematically increasing polynomial orders, fully spanning all possible structural uncertainty by encompassing the entire set of possible models. This is the basic idea behind the process rate equations in BOSS, which are formulated via generalized power series that encompass the set of all polynomial functions (Morrison et al., 2020). Note that complexity can also be built up systematically by increasing the number of independent variables and hence degrees of freedom within the model. Results from a very simple “known truth” experiment are shown in Figure 15 to illustrate the ideas of model expansion and parsimony. This type of approach could begin to address the problem of structural uncertainty rigorously, which up to this point has been more or less ignored, not just for microphysics schemes but for physical parameterizations in weather and climate models more generally. There may still be philosophical questions as to whether this is truly inductive learning, but as a practical matter, this is irrelevant; we seek to obtain a model that describes (well) a set of observed phenomena, whether or not that model is a correct representation of some ethereal set of “true” physical laws.

**Figure 16:**
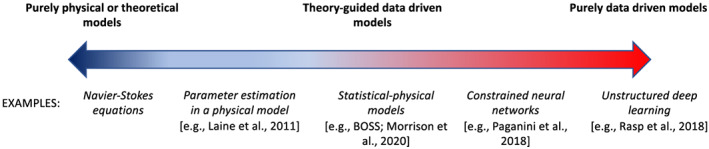
Illustration of the continuum of modeling approaches, from *purely physical/theoretical* approaches on the left to *purely data driven unstructured* approaches on the right. In the center are *theory‐guided data driven* approaches which combine elements from physical and statistical modeling. Examples across the continuum are provided below the arrow.

Following this logic, we envision a Bayesian approach to scheme development built successively on “known” physical truths and theory and incorporating the ideas of model checking and model expansion with complexity added and tested systematically. If additional assumptions can be made, such as smoothness in the relationship between process rates and state variables, mathematical rigor can be applied in building up scheme complexity. To put it simply, the idea is to meet in the middle ground between purely physical models and purely statistical models (Figure 16). There are some commonalities with the climate model parameterization approach proposed by Schneider et al. (2017), who similarly advocated for parameter learning within a well‐defined physical framework. More generally, this type of statistical‐physical approach has emerged recently in other scientific disciplines such as turbulence modeling, materials, and quantum chemistry and has been referred to as “theory‐guided data science” (Karpatne et al., 2017).

There are some practical challenges for parameterization development within a Bayesian framework. Perhaps foremost are those related to the use of Bayesian methods with full weather and climate models. MCMC, while rigorous, is computationally expensive and requires a huge number of model runs for many problems. This is not insurmountable for simple models, or even fully 3D models with small domains (Posselt, 2016), but becomes practically intractable for typical weather or climate model simulation lengths and domain sizes. Estimation of posterior distributions is also inherently challenging for cloud and weather models in the face of state uncertainty. This is closely related to the problem of initial condition uncertainty and the rapid growth of small errors (i.e., inherently limited predictability), which may render the calculation of full posterior parameter PDFs or even parameter estimation practically untenable once state errors grow too large. State error growth can be reduced using data assimilation, but this presents other challenges; moreover, inclusion of an explicit assimilation component for reducing state error would add significant complexity to the infrastructure of any Bayesian framework for scheme development and parameter estimation. Nonetheless, methods for simultaneous state and parameter estimation have been recently demonstrated (e.g., Laine et al., 2011) and could be pursued in the context of microphysics. Alternatively, applying Bayesian inference at climate scales to estimate posterior parameter distributions, based on longer‐term statistical model output rather than simulation of specific weather events, has some potential to alleviate this problem. However, long model integrations make the problem of computational tractability for Bayesian methods even worse. The challenges of using Bayesian inference are especially acute for bin and Lagrangian particle‐based schemes because of their computational expense. To our knowledge, no attempt has yet been made to test bin and Lagrangian particle‐based microphysics within a Bayesian framework.

One possible solution to the daunting computational challenge inherent in Bayesian model development is to use emulation, which replaces the full‐complexity physical model with a functional or statistical approximation called a *surrogate* model. This has been applied previously for parameter sensitivity analysis using Gaussian process emulation (e.g., Carslaw et al., 2013; He et al., 2018; Johnson et al., 2015; Posselt et al., 2016). Other emulation approaches include polynomial chaos expansion (Iskandarani et al., 2016; Marzouk & Najm, 2009; Sraj et al., 2016) and multivariate adaptive regression splines (MARS; Friedman, 1991; Friedman & Roosen, 1995). Recently developed neural network and other unstructured machine learning approaches (see below) could also be used to more flexibly and generally emulate the model. Surrogate models and emulators enable a far greater number of computations because they are much more computationally efficient compared with the full complexity model. However, there are potential limitations that should be kept in mind. First, surrogate models are necessarily trained on a limited number of full model realizations. This means, in practice, that they are better at interpolation than extrapolation; they often find realistic solutions within the range of parameters the model has been trained on but may fail when asked to extrapolate beyond the training data set. Second, emulation often has built‐in assumptions; for example, linearity or a smooth functional relationship between the model input and output. If these assumptions are violated, the surrogate model may not be able to realistically reproduce the full‐complexity model behavior. Even so, well‐trained surrogate models may be the only possible way forward to probe the full multidimensional parameter space of detailed schemes in modern cloud models, given the large number of degrees of freedom and computational expense of running such models.

**Figure 17:**
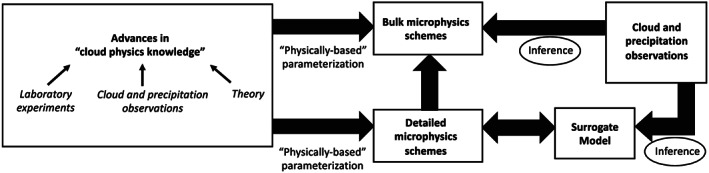
Conceptual diagram of the proposed hierarchical statistical‐physical approach for microphysics scheme development. In the proposed approach, laboratory experiments, natural cloud and precipitation observations, and theory improve cloud physics knowledge (left box). This directly informs physically based parameterizations for bulk and detailed (bin or Lagrangian particle‐based) schemes. Emulation is used to develop a surrogate model of the response to parameter changes within the detailed scheme. The surrogate model is constrained by natural cloud and precipitation observations using Bayesian inference, which informs parameter distributions in the detailed scheme. The constrained detailed scheme together with advances in cloud physics knowledge inform the physical framework for the bulk scheme. Bayesian inference is then used to constrain parameters and structure of the bulk scheme via cloud and precipitation observations.

Combining surrogate models, Bayesian inference, and detailed process modeling leads to several paths for hierarchical microphysics scheme development (Figure 17). This contrasts with the traditional approach of scheme development, which relies heavily on heuristics and ad hoc tuning (Figure 8). Overall, the basic idea is to combine statistical and physical tools optimally, in a way that leverages advances in emulation, machine learning, and Bayesian methods, process modeling such as Lagrangian particle‐based schemes, and fundamental cloud physics knowledge. One could imagine, for example, developing a surrogate model (via process emulation, machine learning, etc.) from training data generated using a Lagrangian particle‐based scheme to emulate the scheme response to changes in parameter values. Bayesian inference could be used with the surrogate model to calculate posterior parameter PDFs constrained by cloud and precipitation observations (lower right in Figure 17), providing not only maximum a posteriori (optimal) parameter values but also rigorous quantification of uncertainty. The surrogate model could itself serve as a basis for parameterization. However, to avoid limitations of the surrogate model, namely, the lack of a physically based framework and hence the danger of extrapolating to conditions outside of the training data, the full‐complexity particle‐based scheme with the observationally constrained posterior parameter PDFs from the surrogate model could be used, in addition to fundamental cloud physics knowledge, to inform the physical framework and prior parameter distributions for bulk scheme development. The bulk scheme, in terms of both parameters and structure, could in turn be constrained using a wider set of observations via Bayesian inference in the context of weather and climate simulations (upper right in Figure 17).

### 4.4 Machine Learning

Another approach that has gained recent traction within the atmospheric science community has been to develop parameterizations wholly via *machine learning* (e.g., Brenowitz & Bretherton, 2018; Gentine et al., 2018; O’Gorman & Dwyer, 2018; Rasp et al., 2018). These approaches replace the entire parameterization with a data‐driven neural network or statistical model. This approach has met with some success as a replacement for traditional cloud and convection parameterizations within global climate models. For example, Rasp et al. (2018) trained their data‐driven parameterization on data from a multiscale “super‐parameterized” global model in which convection is treated explicitly and were able to reasonably approximate physical constraints (e.g., energy conservation) that were not explicitly built into the parameterization. Nonetheless, there are criticisms of using purely data‐driven approaches for parameterization development (e.g., Karpatne et al., 2017): (i) lack of *interpretability* owing to the “black box” nature of machine learning, that is, the difficulty of using machine learning to gain physical insight into the system being modeled; (ii) often poor performance when extrapolating to conditions outside of the original training data set; (iii) limited quantification of parameterization uncertainty; and (iv) inconsistency with physical constraints. Obtaining a sufficient and robust training data set for the development of a purely data‐driven microphysics scheme is likely to be especially difficult or even impossible because there is no benchmark model with which to generate a robust training dataset.

We note that there are ways to address some of the above criticisms. In particular, physical constraints such as water and energy conservation can be incorporated directly into a machine learning approach using, for example, constrained neural networks (e.g., Paganini et al., 2018). Bayesian neural networks (e.g., Auld et al., 2007; Titterington, 2004) can also provide information about uncertainty. Nonetheless, interpretability and extrapolation will likely remain difficult problems for any microphysics scheme that is built using a purely data‐driven machine learning approach, without an underlying physical framework. Though there are important gaps in cloud physics knowledge, we argue that it is important to include in schemes the knowledge that is available.

In contrast to a purely data‐driven parameterization approach, using machine learning in conjunction with a physical model, for example by “learning” parameter values within the model (i.e., parameter estimation), would help to address both the lack of interpretability and problem of extrapolation. This idea is consistent with a broader definition of “machine learning” as any data‐driven approach to modeling, as advocated by Schneider et al. (2017) and Karpatne et al. (2017), rather than referring only to *unstructured* data‐driven methods. The Bayesian statistical‐physical approach advocated above falls into this broader paradigm of *theory‐guided data science* (Karpatne et al., 2017; see Figure 16). That said, we note the potential for unstructured machine learning as a tool for emulation of bin and Lagrangian particle‐based microphysics schemes as discussed above, not as a parameterization itself but rather to explore the behavior of these schemes (see section 4.3 and Figure 17).

## 5 Conclusions and Broader Outlook

Microphysics is a key component of cloud, weather, and climate models. It has arguably taken on an even more important role as these models have steadily increased in resolution and as the coupling of microphysics and cloud dynamics has become more direct. A characteristic feature of microphysics is its extreme complexity, involving myriad interacting microscale processes and complicated feedbacks between hydrometeors and their environment over a wide range of scales. Owing to this, the representation of microphysics in atmospheric models is beset by a number of challenges. In this paper, we divided these challenges into two distinct categories: (i) how to parameterize the population of hydrometeors in a model grid volume given that it is computationally impossible to represent all hydrometeors individually, even for small cloud volumes; and (ii) how to address fundamental process uncertainties at the scale of individual hydrometeors.

The first aspect—a manifestation of the classical *parameterization problem* of representing unresolved, subgrid‐scale model features—is centered on developing improved methods to represent hydrometeor populations that cannot be modeled on an individual particle‐by‐particle basis. Traditional Eulerian bulk and bin approaches have been used since the mid‐20th century and remain the workhorses of nearly all models. Bin schemes provide many degrees of freedom to represent hydrometeor populations and can simulate microphysical evolution in detail, but face a number of challenges that are difficult to overcome. Bulk schemes are computationally efficient and will remain the mainstay of operational weather and climate modeling, but the analytic functions (or empirical moment relationships) assumed for the particle SDs, needed for closing the set of microphysical equations, are uncertain. Given the lack of a solid theoretical foundation, these distribution functions are determined empirically from observations or detailed model simulations (i.e., using bin schemes). Although not the focus of this paper, we also emphasize the challenge of parameterizing cloud *macrophysics*—the grid cell fractional cloudiness and subgrid‐scale distributions of bulk cloud, precipitation, and thermodynamic quantities and vertical motion directly coupled to the microphysics in larger‐scale models.

In contrast to bin and bulk approaches, a new tool emerged in the early 21st century: the Lagrangian particle‐based method. This approach addresses several practical challenges of bin schemes. We also emphasize an important conceptual advantage: as the number of “super‐particles” approaches the number of actual cloud particles, and the model resolution approaches that of DNS, the Lagrangian particle‐based approach converges to particle‐by‐particle DNS, which is the most complete model representation of a turbulent cloud currently available. The main difficulty with Lagrangian particle‐based schemes is the computational cost, although there are some methods to help mitigate this problem. Lagrangian particle‐based schemes will likely come into much wider use in the coming years for research, and we anticipate this approach becoming a staple of cloud modeling within the next decade. We note the potential for these schemes to be used not only for probing fundamental research questions in cloud physics, but also for developing and testing bulk schemes for use in operational weather and climate models. Lagrangian particle‐based schemes can also be used for comparison with bin microphysics schemes in research models. Such work is ongoing, and we expect it to accelerate in the coming years. This can address important questions, such as under what circumstances (if any) will these types of schemes produce similar results? What are specific effects of numerical errors and approximations in bin schemes, relative to Lagrangian particle‐based schemes?

The second critical challenge—fundamental process‐level uncertainty and complexity—is closely related to gaps in cloud physics knowledge. This is particularly true for ice‐phase microphysics, owing mainly to the intricacies of particle shape and density evolution. Ultimately, this problem stems from the fact that theoretical descriptions of many microphysical processes are limited, and there is no complete set of governing equations or benchmark model for microphysics. We highlighted several specific gaps in cloud physics understanding in section 3.2 and how these gaps contribute to scheme uncertainty. We emphasize a critical point: These knowledge gaps lead to uncertainty in *all* models, regardless of how they represent the hydrometeor population; this includes Lagrangian particle‐based schemes and even particle‐by‐particle DNS. Confronting this problem is therefore a necessary step to address overall scheme uncertainty and ultimately to improve models.

Although theoretical and process‐level descriptions are limited for many microphysical processes owing to these knowledge gaps, there is now a wealth of cloud and precipitation observations for evaluating schemes. Unfortunately, individual process rates within schemes generally cannot be obtained from these observations directly. We therefore argue that it is useful to frame microphysics as an inverse problem that uses observations to constrain schemes indirectly by way of comparison with model output. Framed in this way, it is natural to bring to bear tools from statistical modeling. We also emphasize that cloud and precipitation microphysical processes constitute a physical system. As such, there are important physical constraints that, although currently limited, should be incorporated. Thus, broadly speaking, we advocate approaches that pursue a “middle ground” by incorporating elements of both physical and statistical modeling (Figure 16). This is distinct from purely physical models, such as those that numerically solve the Navier‐Stokes equations for fluid flow, yet also different from “black box” unstructured machine learning approaches that have been advocated in recent years as an avenue for parameterization development. We highlighted a statistical‐physical framework that combines physical and theoretical insight with Bayesian inference to produce a scheme that is capable of continuous updates as new theory and observations become available. Bayesian inference has an additional and important benefit, in that it produces rigorous quantitative estimates of scheme uncertainty. This type of general statistical‐physical framework, falling under the broad umbrella of *theory‐guided data science* (Karpatne et al., 2017), may also be relevant to other physical parameterizations in weather and climate models, especially those that lack complete governing equations or a benchmark model similar to microphysics, such as land surface schemes.

Following the ideas outlined in this paper, we highlight six specific recommendations for advancing the representation of microphysics in models:

1. *Sustained support for laboratory facilities to study microphysical processes*, addressing major gaps in cloud physics knowledge and providing data to develop physically based parameterizations and to support or refute cloud physics theories.
2. *Sustained support for new airborne and ground‐based instrument development and next‐generation instruments in space* to provide the field data that are required to constrain microphysics in global as well as regional models.
3. *Increased emphasis on critical evaluation of model performance using field observations*, including statistically robust sampling from in situ or remote‐sensing approaches and targeted data collection in well‐defined regions where microphysical properties can be robustly characterized for model evaluation.
4. *Development of new frameworks to facilitate rigorous model evaluation and constraint by observations*, leveraging statistical modeling tools and accounting for observational uncertainty characteristics. This includes the use of machine learning, not as a replacement for microphysics schemes but as a tool to understand scheme behavior (e.g., via emulation).
5. *Increased focus on systematic quantification of parameter and structural uncertainty in schemes*, which can help direct efforts for scheme improvement and point to particular needs for observational constraint.
6. *Continued development and use of new methods for microphysical modeling*, especially Lagrangian particle‐based schemes.

More broadly, we envision a hierarchical approach for microphysics scheme development that ties together the various pieces advocated in this paper (Figure 17). This contrasts with the “traditional” approach for scheme development that relies heavily on heuristics and (often ad hoc) tuning (Figure 8). A crucial element of both approaches is the incorporation of advances in cloud physics knowledge gained from laboratory experimentation, natural cloud and precipitation observations, and theory. The ultimate ideal is to develop schemes entirely from a “complete” body of cloud physics knowledge, but it is not clear when that might be achievable—likely decades into the future, if ever. This underlies our basic argument for why it may be useful to incorporate a statistical element into scheme development.

In the proposed approach, centered on the idea of microphysics parameterization as an inverse problem, scheme development is constrained by cloud and precipitation observations via Bayesian inference within the confines of a physically based framework (the latter informed by fundamental cloud physics knowledge and, for bulk schemes, detailed Lagrangian particle‐based and bin schemes). There is a two‐way street in addressing this inverse problem, especially in a probabilistic framework: While observations constrain the scheme, this approach could also provide a way to quantify rigorously what information is gained from particular measurements, helping to guide future observational efforts.

Incorporating these statistical elements does not have to be limited to the development of bulk schemes. Natural cloud and precipitation observations could also be used to constrain detailed bin and Lagrangian particle‐based schemes themselves, although solving this inverse problem is more challenging from a technical standpoint owing to the computational cost of these schemes. It may be worthwhile to explore the use of much less costly surrogate models of these detailed schemes via *emulation* as a way to make the problem computationally tractable. Leveraging advances in computing infrastructure, such as developing schemes that can be run on GPUs, should also be pursued. There are other technical and practical challenges that need to be worked out for this kind of hierarchical statistical‐physical approach. Nonetheless, it provides a possible blueprint for accelerating progress in how microphysics is represented in models.

## Data Availability

All new data generated for this paper (shown in Figures 6, 9, and 14) and the accompanying metadata files are stored in a repository and can be accessed online (10.5065/mn1v‐6a55).

## References

[jame21128-bib-0001] Abade, G. C. , Grabowski, W. W. , & Pawlowska, H. (2018). Broadening of cloud droplet spectra through eddy hopping: Turbulent entraining parcel simulations. Journal of the Atmospheric Sciences, 75, 3365–3379. 10.1175/jas-d-18-0078.1

[jame21128-bib-0002] Abdelmonem, A. , Järvinen, E. , Duft, D. , Hirst, E. , Vogt, S. , Leisner, T. , & Schnaiter, M. (2016). PHIPS–HALO: The airborne particle habit imaging and polar scattering probe—Part 1: Design and operation. Atmospheric Measurement Techniques, 9, 3131–3144. 10.5194/amt-9-3131-2016

[jame21128-bib-0003] Abdelmonem, A. , Lützenkirchen, J. , & Leisner, T. (2015). Probing ice‐nucleation processes on the molecular level using second harmonic generation spectroscopy. Atmospheric Measurement Techniques, 8, 3519–3526. 10.5194/amt-8-3519-2015

[jame21128-bib-0004] Ackerman, A. S. , Kirkpatrick, M. P. , Stevens, D. E. , & Toon, O. B. (2004). The impact of humidity above stratiform clouds on indirect aerosol climate forcing. Nature, 432, 1014–1017. 10.1038/nature03174 15616559

[jame21128-bib-0005] Adams‐Selin, R. D. , van den Heever, S. C. , & Johnson, R. H. (2013). Impact of graupel parameterization schemes on idealized bow echo simulations. Monthly Weather Review, 141, 1241–1262. 10.1175/mwr-d-12-00064.1

[jame21128-bib-0006] Alexandrov, M. D. , Cairns, B. , Emde, C. , Ackerman, A. S. , & van Diedenhoven, B. (2012). Accuracy assessments of cloud droplet size retrievals from polarized reflectance measurements by the research scanning polarimeter. Remote Sensing of Environment, 125, 92–111. 10.1016/j.rse.2012.07.012

[jame21128-bib-0007] Andrejczuk, M. , Grabowski, W. W. , Malinowski, S. P. , & Smolarkiewicz, P. K. (2009). Numerical simulation of cloud‐clear air interfacial mixing: Homogeneous versus inhomogeneous mixing. Journal of the Atmospheric Sciences, 66, 2493–2500. 10.1175/2009jas2956.1

[jame21128-bib-0008] Andrejczuk, M. , Grabowski, W. W. , Reisner, J. , & Gadian, A. (2010). Cloud‐aerosol interactions for boundary layer stratocumulus in the Lagrangian cloud model. Journal of Geophysical Research: Atmospheres, 115, D22214 10.1029/2010JD014248

[jame21128-bib-0009] Andrejczuk, M. , Reisner, J. M. , Henson, B. , Dubey, M. K. , & Jeffery, C. A. (2008). The potential impacts of pollution on a non‐drizzling stratus deck: Does aerosol number matter more than type? Journal of Geophysical Research: Atmospheres, 113, D19204 10.1029/2007JD009445

[jame21128-bib-0010] Andric, J. , Kumjian, M. R. , Zrnic, D. S. , Straka, J. M. , & Melnikov, V. M. (2013). Polarimetric signatures above the melting layer in winter storms: An observational and modeling study. Journal of Applied Meteorology and Climatology, 52, 682–700. 10.1175/JAMC-D-12-028.1

[jame21128-bib-0011] Arabas, S. , & Shima, S. (2013). Large‐eddy simulations of trade wind cumuli using particle‐based microphysics with Monte Carlo coalescence. Journal of the Atmospheric Sciences, 70, 2768–2777. 10.1175/JAS-D-12-0295.1

[jame21128-bib-0012] Aristov, V. V. , Voronich, I. V. , & Zabelok, S. A. (2019). Direct methods for solving the Boltzmann equations: Comparisons with direct simulation Monte Carlo and possibilities. Physics of Fluids, 31, 097106 10.1063/1.5108670

[jame21128-bib-1003] Aufdermaur, A. N. , & Johnson, D. A. (1972). Charge separation due to riming in an electric field. 98, 369–382, 10.1002/qj.49709841609

[jame21128-bib-0013] Auld, T. , Moore, A. W. , & Gull, S. F. (2007). Bayesian neural networks for internet traffic classification. IEEE Transactions on Neural Networks, 18, 223–239. 10.1109/tnn.2006.883010 17278474

[jame21128-bib-0014] Bacon, N. J. , Swanson, B. D. , Baker, M. B. , & Davis, E. J. (1998). Breakup of levitated frost particles. Journal of Geophysical Research: Atmospheres, 103(D12), 13,763–13,775. 10.1029/98JD01162

[jame21128-bib-0015] Bailey, M. , & Hallett, J. (2002). Nucleation effects on the habit of vapor grown ice crystals from −18 to −42°C. Quarterly Journal of the Royal Meteorological Society, 128, 1461–1483. 10.1002/qj.200212858304

[jame21128-bib-0016] Bailey, M. , & Hallett, J. (2004). Growth rates and habits of ice crystals between 2208 and 2708°C. Journal of the Atmospheric Sciences, 61, 514–544. 10.1175/1520-0469(2004)061,0514:GRAHOI.2.0.CO;2

[jame21128-bib-0017] Baker, M. , Corbin, R. G. , & Latham, J. (1980). The influence of entrainment on the ervoliution of cloud drop spectra: I: A model of inhomogeneous mixing. Quarterly Journal of the Royal Meteorological Society, 106, 581–598. 10.1002/qj.49710644914

[jame21128-bib-0018] Ban‐Weiss, G. A. , Jin, L. , Bauer, S. E. , Bennartz, R. , Liu, X. , Zhang, K. , Ming, Y. , Guo, Y. , & Jiang, J. H. (2014). Evaluating clouds, aerosols, and their interactions in three global climate models using satellite simulators and observations. Journal of Geophysical Research, 119, 10,876–10,901. 10.1002/2014JD021722

[jame21128-bib-0019] Barahona, D. , Rodriguez, J. , & Nenes, A. (2010). Sensitivity of the global distribution of cirrus ice crystal concentration to heterogeneous freezing. Journal of Geophysical Research: Atmospheres, 115, D23213 10.1029/2010JD014273

[jame21128-bib-0020] Barrett, A. I. , Westbrook, C. D. , Nicol, J. C. , & Stein, T. H. M. (2019). Rapid ice aggregation process revealed through triple‐wavelength Doppler spectrum radar analysis. Atmospheric Chemistry and Physics, 19, 5753–5769. 10.5194/acp-19-5753-2019

[jame21128-bib-0021] Barros, A. P. , Prat, O. P. , Shrestha, P. , Testik, F. Y. , & Bliven, L. F. (2008). Revisiting Low and List (1982): Evaluation of raindrop collision parameterizations using laboratory observations and modeling. Journal of the Atmospheric Sciences, 65, 2983–2993. 10.1175/2008jas2630.1

[jame21128-bib-0022] Baum, B. A. , Yang, P. , Heymsfield, A. J. , Schmitt, C. G. , Xie, Y. , Bansemer, A. , Hu, Y. X. , & Zhang, Z. (2011). Improvements in shortwave bulk scattering and absorption models for the remote sensing of ice clouds. Journal of Applied Meteorology and Climatology, 50, 1037–1056. 10.1175/2010JAMC2608.1

[jame21128-bib-0023] Baumgardner, D. , Abel, S. J. , Axisa, D. , Cotton, R. , Crosier, J. , Field, P. , Gurganus, C. , Heymsfield, A. , Korolev, A. , Krämer, M. , Lawson, P. , McFarquhar, G. , Ulanowski, Z. , & Um, J. (2017). Cloud ice properties: In situ measurement challenges. Meteorological Monographs, 58, 9.1–9.23. 10.1175/AMSMONOGRAPHS-D-16-0011.1

[jame21128-bib-0024] Beard, K. V. (1992). Ice initiation in warm‐base convective clouds: An assessment of microphysical mechanisms. Atmospheric Research, 28, 125–152. 10.1016/0169-8095(92)90024-5

[jame21128-bib-0025] Beard, K. V. , & Ochs, H. T. (1995). Collisions between small precipitation drops. Part II: Formulas for coalescence, temporary coalescence, and satellites. Journal of the Atmospheric Sciences, 52, 3977–3996. 10.1175/1520-0469(1995)052<3977:cbspdp>2.0.co;2

[jame21128-bib-0026] Beard, K. V. , & Pruppacher, H. R. (1971). A wind tunnel investigation of the rate of evaporation of small water drops falling at terminal velocity in air. Journal of the Atmospheric Sciences, 28, 1455–1464. 10.1175/1520-0469(1971)028<1455:awtiot>2.0.co;2

[jame21128-bib-0027] Beheng, K. D. , Jellinghaus, K. , Sander, W. , Roth, N. , & Weigand, B. (2006). Investigation of collision‐induced breakup of raindrops by numerical simulations: First results. Geophysical Research Letters, 33, L10811 10.1029/2005GL025519

[jame21128-bib-0028] Benjamin, S. G. , Weygandt, S. S. , Brown, J. M. , Hu, M. , Alexander, C. R. , Smirnova, T. G. , Olson, J. B. , James, E. P. , Dowell, D. C. , Grell, G. A. , Lin, H. , Peckham, S. E. , Smith, T. L. , Moninger, W. R. , Kenyon, J. S. , & Manikin, G. S. (2016). A North American hourly assimilation and forecast cycle: The Rapid Refresh. Monthly Weather Review, 144, 1669–1694. 10.1175/mwr-d-15-0242.1

[jame21128-bib-0029] Bergeron, T. (1935). On the physics of cloud and precipitation, Proc. 5th Assembly U.G.G.I. Lisbon, Vol. 2, p. 156.

[jame21128-bib-0030] Berner, J. , Fossell, K. R. , Ha, S.‐Y. , Hacker, J. P. , & Snyder, C. (2015). Increasing the skill of probabilistic forecasts: Understanding performance improvements from model‐error representations. Monthly Weather Review, 143, 1295–1320. 10.1175/mwr-d-14-00091.1

[jame21128-bib-0031] Berry, E. X. (1967). Cloud droplet growth by collection. Journal of the Atmospheric Sciences, 24, 688–701. 10.1175/1520-0469(1967)024<0688:cdgbc>2.0.co;2

[jame21128-bib-0032] Berry, E. X. (1969). A mathematical framework for cloud models. Journal of the Atmospheric Sciences, 26, 109–111. 10.1175/1520-0469(1969)026<0109:amffcm>2.0.co;2

[jame21128-bib-0033] Berry, E. X. , & Reinhardt, R. L. (1974a). An analysis of cloud drop growth by coalescence: Part 1. Double distributions. Journal of the Atmospheric Sciences, 31, 1814–1824. 10.1175/1520-0469(1974)031<1814:aaocdg>2.0.co;2

[jame21128-bib-0034] Berry, E. X. , & Reinhardt, R. L. (1974b). An analysis of cloud drop growth by coalescence: Part II. Single initial distributions. Journal of the Atmospheric Sciences, 31, 1825–1831. 10.1175/1520-0469(1974)031<1825:aaocdg>2.0.co;2

[jame21128-bib-0035] Berry, E. X. , & Reinhardt, R. L. (1974c). An analysis of cloud drop growth by coalescence: Part III. Accretion and self‐collection. Journal of the Atmospheric Sciences, 31, 2118–2126. 10.1175/1520-0469(1974)031<2118:aaocdg>2.0.co;2

[jame21128-bib-0036] Berry, E. X. , & Reinhardt, R. L. (1974d). An analysis of cloud drop growth by coalescence: Part IV. A new parameterization. Journal of the Atmospheric Sciences, 31, 2127–2135. 10.1175/1520-0469(1974)031<2127:aaocdg>2.0.co;2

[jame21128-bib-0037] Bessho, K. , Date, K. , Hayashi, M. , Ikeda, A. , Imai, T. , Inoue, H. , Kumagai, Y. , Miyakawa, T. , Murata, H. , Ohno, T. , Okuyama, A. , Oyama, R. , Sasaki, Y. , Shimazu, Y. , Shimoji, K. , Sumida, Y. , Suzuki, M. , Taniguchi, H. , Tsuchiyama, H. , Uesawa, D. , Yokota, H. , & Yoshida, R. (2016). An introduction to Himawari‐8/9—Japan's new‐generation geostationary meteorological satellites. Journal of the Meteorological Society of Japan, 94, 151–183. 10.2151/jmsj.2016-009

[jame21128-bib-0038] Best, M. J. , Abramowitz, G. , Johnson, H. R. , Pitman, A. J. , Balsamo, G. , Boone, A. , Cuntz, M. , Decharme, B. , Dirmeyer, P. A. , Dong, J. , Ek, M. , Guo, Z. , Haverd, V. , van den Hurk, B. J. J. , Nearing, G. S. , Pak, B. , Peters‐Lidard, C. , Santanello, J. A. , Stevens, L. , & Vuichard, N. (2015). The plumbing of land surface models: Benchmarking model performance. Journal of Hydrometeorology, 16, 1425–1442. 10.1175/jhm-d-14-0158.1 PMC5884676 29630073

[jame21128-bib-0039] Beven, K. , Smith, P. , & Freer, J. (2007). Comment on “hydrological forecasting uncertainty assessment: Incoherence of the GLUE methodology” by Pietro Mantovan and Ezio Todini. Journal of Hydrology, 338, 315–318. 10.1016/j.jhydrol.2007.02.023

[jame21128-bib-0040] Beven, K. J. (1993). Prophecy, reality and uncertainty in distributed hydrological modeling. Advances in Water Resources, 16, 41–51. 10.1016/0309-1708(93)90028-e

[jame21128-bib-0041] Bigg, E. K. (1953). The supercooling of water. Proceedings of the Physical Society. Section B, 66, 688 10.1088/0370-1301/66/8/309

[jame21128-bib-0042] Bigg, E. K. (1957). The fragmentation of freezing water drop. Bulletin de l’Observatoire du Puy‐de‐Dôme, N3(65–69), 1957.

[jame21128-bib-0043] Bird, G. A. (1963). Approach to translational equilibrium in a rigid sphere gas. Physics of Fluids, 6, 1518–1519. 10.1063/1.1710976

[jame21128-bib-0044] Bodas‐Salcedo, A. , Mulcahy, J. P. , Andrews, T. , Williams, K. D. , Ringer, M. A. , Field, P. R. , & Elsaesser, G. S. (2019). Strong dependence of atmospheric feedbacks on mixed‐phase microhysics and aerosol‐cloud interactions. Journal of Advances in Modeling Earth Systems, 11, 1735–1758. 10.1029/2019MS001688 31598189 PMC6774284

[jame21128-bib-0045] Bodas‐Salcedo, A. , Webb, M. J. , Bony, S. , Chepfer, H. , Dufresne, J.‐L. , Klein, S. A. , Zhang, Y. , Marchand, R. , Haynes, J. M. , Pincus, R. , & John, V. O. (2011). Cosp: Satellite simulation software for model assessment. Bulletin of the American Meteorological Society, 92, 1023–1043. 10.1175/2011BAMS2856.1

[jame21128-bib-0046] Bower, K. , Moss, S. J. , Johnson, D. W. , Choularton, T. W. , Latham, J. , Brown, P. R. A. , Blyth, A. M. , Cardwell, J. , & J. (1996). A parametrization of ice water content observed in frontal and convective clouds. Quarterly Journal of the Royal Meteorological Society, 122, 1815–1844. 10.1002/qj.49712253605

[jame21128-bib-0047] Box, G. E. P. (1980). Sampling and Bayes' inference in scientific modelling and robustness. Journal of the Royal Statistical Society, Series A, 143, 383–430. 10.2307/2982063

[jame21128-bib-0048] Braham, R. R. (1964). What is the role of ice in summer rain‐showers? Journal of the Atmospheric Sciences, 21(6), 640–645. 10.1175/1520-0469(1964)021<0640:WITROI>2.0.CO;2

[jame21128-bib-0049] Brandes, E. A. (1977). Flow in severe thunderstorms observed by dual‐Doppler radar. Monthly Weather Review, 105, 113–120. 10.1175/1520-0493(1977)105<0113:fistob>2.0.co;2

[jame21128-bib-0050] Brandes, E. A. , Ryzhkov, A. V. , & Zrnic, D. S. (2001). An evaluation of radar rainfall estimates from specific differential phase. Journal of Atmospheric and Oceanic Technology, 18, 363–375. 10.1175/1520-0426(2001)018<0363:aeorre>2.0.co;2

[jame21128-bib-0052] Brdar, S. , & Seifert, A. (2018). McSnow: A Monte‐Carlo particle model for riming and aggregation of ice particles in a multidimensional microphysical phase space. Journal of Advances in Modeling Earth Systems, 10, 187–206. 10.1002/2017ms001167

[jame21128-bib-0053] Brenowitz, N. D. , & Bretherton, C. S. (2018). Prognostic validation of a neural network unified physics parameterization. Geophysical Research Letters, 45, 6289–6298. 10.1029/2018GL078510

[jame21128-bib-0054] Brewer, A. W. , & Palmer, H. P. (1949). Condensation processes at low temperatures, and the production of new sublimation nuclei by the splintering of ice. Nature, 164(4164), 312–313. 10.1038/164312a0

[jame21128-bib-0055] Bringi, V. N. , & Chandrasekar, V. (2001). Polarimetric Doppler weather radar: Principles and applications (636 pp). Cambridge, U.K.: Cambridge University Press.

[jame21128-bib-0056] Bringi, V. N. , Huang, G. J. , Chandrasekar, V. , & Keenan, T. D. (2001). An areal rainfall estimator using differential propagation phase: Evaluation using a C‐band radar and a dense gauge network in the tropics. Journal of Atmospheric and Oceanic Technology, 18, 1810–1818. 10.1175/1520-0426(2001)018<1810:aareud>2.0.co;2

[jame21128-bib-0057] Bringi, V. N. , Rasmussen, R. M. , & Vivekanandan, J. (1986). Multiparameter radar measurements in Colorado convective storms. Part II: Hail detection studies. Journal of the Atmospheric Sciences, 43, 2564–2577. 10.1175/1520-0469(1986)043<2564:mrmicc>2.0.co;2

[jame21128-bib-0058] Brown, P. S. (1986). Analysis of the Low and List drop‐breakup formulation. Journal of Climate and Applied Meteorology, 25, 313–321. 10.1175/1520-0450(1986)025<0313:aotlal>2.0.co;2

[jame21128-bib-0061] Brown, P. S. (1993). Analysis and parameterization of the combined coalescence, breakup, and evaporation processes. Journal of the Atmospheric Sciences, 50, 2940–2951. 10.1175/1520-0469(1993)050<2940:aapotc>2.0.co;2

[jame21128-bib-0064] Bryan, G. , & Morrison, H. (2012). Sensitivity of a simulated squall line to horizontal resolution and parameterization of microphysics. Monthly Weather Review, 140, 202–225. 10.1175/mwr-d-11-00046.1

[jame21128-bib-0065] Bukovčić, P. , Ryzhkov, A. , Zrnić, D. , & Zhang, G. (2018). Polarimetric radar relations for quantification of snow based on disdrometer data. Journal of Applied Meteorology and Climatology, 57, 103–120. 10.1175/JAMC-D-17-0090.1

[jame21128-bib-0068] Carslaw, K. S. , Lee, L. A. , Reddington, C. L. , Pringle, K. J. , Rap, A. , Forster, P. M. , Mann, G. W. , Spracklen, D. V. , Woodhouse, M. T. , Regayre, L. A. , & Pierce, J. R. (2013). Large contribution of natural aerosols to uncertainty in indirect forcing. Nature, 503, 67–71. 10.1038/nature12674 24201280

[jame21128-bib-0069] Chandrakar, K. K. , Cantrell, W. , Chang, K. , Ciochetto, D. , Niedermeier, D. , Ovchinnikov, M. , Shaw, R. A. , & Yang, F. (2016). Turbulence‐induced cloud‐aerosol indirect effect. Proceedings of the National Academy of Sciences, 113(50), 14,243–14,248. 10.1073/pnas.1612686113 PMC5167139 27911802

[jame21128-bib-0070] Chandrakar, K. K. , Cantrell, W. , Ciochetto, D. , Karki, S. , Kinney, G. , & Shaw, R. A. (2017). Aerosol removal and cloud collapse accelerated by supersaturation fluctuations in turbulence. Geophysical Research Letters, 44, 4359–4367. 10.1002/2017GL072762

[jame21128-bib-0071] Chandrakar, K. K. , Cantrell, W. , Kostinski, A. B. , & Shaw, R. A. (2018). Dispersion aerosol indirect effect in turbulent clouds: Laboratory measurements of effective radius. Geophysical Research Letters, 45, 10,738–10,745. 10.1029/2018GL079194 PMC6376200 30778268

[jame21128-bib-0072] Chandrakar, K. K. , Cantrell, W. , & Shaw, R. A. (2018). Influence of turbulent fluctuations on cloud droplet size dispersion and aerosol indirect effects. Journal of the Atmospheric Sciences, 75, 3191–3209. 10.1175/jas-d-18-0006.1 30631213 PMC6322217

[jame21128-bib-0073] Chang, K. , Bench, J. , Brege, M. , Cantrell, W. , Chandrakar, K. , Ciochetto, D. , Mazzoleni, C. , Mazzoleni, L. R. , Niedermeier, D. , & Shaw, R. A. (2016). A laboratory facility to study gas–aerosol–cloud interactions in a turbulent environment: The Π chamber. Bulletin of the American Meteorological Society, 97, 2343–2358. 10.1175/bams-d-15-00203.1

[jame21128-bib-0074] Chapman, S. , & Cowling, T. G. (1970). The mathematical theory of non‐uniform gases: An account of the kinetic theory of viscosity, thermal conduction and diffusion in gases (3rd ed. Prepared in cooperation with D. Burnett). Cambridge, Eng.: Cambridge University Press.

[jame21128-bib-0075] Chen, J. P. , & Lamb, D. (1999). Simulation of cloud microphysical and chemical processes using a multicomponent framework. Part II: Microphysical evolution of a wintertime orographic cloud. Journal of the Atmospheric Sciences, 56, 2293–2312. 10.1175/1520-0469(1999)056<2293:socmac>2.0.co;2

[jame21128-bib-0076] Chen, J. P. , & Liu, S. T. (2004). Physically based two‐moment bulk water parameterization for warm‐cloud microphysics. Quarterly Journal of the Royal Meteorological Society, 130, 51–78. 10.1256/qj.03.41

[jame21128-bib-0077] Chen, S. , Bartello, P. , Yau, M. K. , Vaillancourt, P. A. , & Zwijsen, K. (2016). Cloud droplet collisions in turbulent environment: Collision statistics and parameterization. Journal of the Atmospheric Sciences, 73(2), 621–636. 10.1175/jas-d-15-0203.1

[jame21128-bib-0078] Chen, S. , Yau, M. K. , & Bartello, P. (2018). Turbulence effects of collision efficiency and broadening of droplet size distribution in cumulus clouds. Journal of the Atmospheric Sciences, 75(1), 203–217. 10.1175/jas-d-17-0123.1

[jame21128-bib-0079] Chen, S. , Yau, M. K. , Bartello, P. , & Xue, L. (2018). Bridging the condensation‐collision size gap: A direct numerical simulation of continuous droplet growth in turbulent clouds. Atmospheric Chemistry and Physics, 18(10), 7251–7262. 10.5194/acp-18-7251-2018

[jame21128-bib-0080] Cheng, A. , & Xu, K. M. (2009). A pdf‐based microphysics parameterization for simulation of drizzling boundary layer clouds. Journal of the Atmospheric Sciences, 66, 2317–2334. 10.1175/2009jas2944.1

[jame21128-bib-0081] Cholette, M. , Morrison, H. , Milbrandt, J. A. , & Theriault, J. M. (2019). Parameterization of the bulk liquid fraction in the predicted particle properties (P3) scheme: Description and idealized tests. Journal of the Atmospheric Sciences, 76, 561–582. 10.1175/jas-d-18-0278.1

[jame21128-bib-0082] Chong, E. , King, M. , Marak, K. E. , & Freedman, M. A. (2019). The effect of crystallinity and crystal structure on the immersion freezing of alumina. The Journal of Physical Chemistry. A, 123(12), 2447–2456. 10.1021/acs.jpca.8b12258 30821971

[jame21128-bib-1004] Choularton, T. W. , Griggs, D. J. , Humood, B. Y. , & Latham, J. (1980). Laboratory studies of riming, and its relation to ice splinter production. Quarterly Journal of the Royal Meteorological Society, 106, 367–374. 10.1002/qj.49710644809

[jame21128-bib-0084] Choularton, T. W. , Latham, J. , & Mason, B. J. (1978). A possible mechanism of ice splinter production during riming. Nature, 274(5673), 791–792. 10.1038/274791a0

[jame21128-bib-0085] Chuang, P. Y. , Saw, E. W. , Small, J. D. , Shaw, R. A. , Sipperley, C. M. , Payne, G. A. , & Bachalo, W. D. (2008). Airborne phase Doppler interferometry for cloud microphysical measurements. Aerosol Science and Technology, 42, 685–703. 10.1175/1520-0426(2002)019<1577:WTTOTA>2.0.CO;2

[jame21128-bib-0086] Cifelli, R. , Chandrasekar, V. , Lim, S. , Kennedy, P. , Wang, Y. , & Rutledge, S. (2011). A new dual‐polarization radar rainfall algorithm: Application in Colorado precipitation events. Journal of Atmospheric and Oceanic Technology, 28, 352–364. 10.1175/2010jtecha1488.1

[jame21128-bib-0087] Clark, A. J. , Weiss, S. J. , Kain, J. S. , Jirak, I. L. , Coniglio, M. , Melick, C. J. , Siewert, C. , Sobash, R. A. , Marsh, P. T. , Dean, A. R. , Xue, M. , Kong, F. , Thomas, K. W. , Wang, Y. , Brewster, K. , Gao, J. , Wang, X. , Du, J. , Novak, D. R. , Barthold, F. E. , Bodner, M. J. , Levit, J. J. , Entwistle, C. B. , Jensen, T. L. , & Correia, J. (2012). An overview of the 2010 Hazardous Weather Testbed experimental forecast program spring experiment. Bulletin of the American Meteorological Society, 93, 55–74. 10.1175/bams-d-11-00040.1

[jame21128-bib-0088] Clark, P. , Choularton, T. W. , Brown, P. R. A. , Field, P. R. , Illingworth, A. J. , & Hogan, R. J. (2005). Numerical modelling of mixed‐phase frontal clouds observed during the CWVC project. Quarterly Journal of the Royal Meteorological Society, 131, 1677–1693. 10.1256/qj.03.210

[jame21128-bib-0089] Clark, T. L. (1974). On modeling nucleation and condensation theory in an eulerian spatial domain. Journal of the Atmospheric Sciences, 31, 2099–2117. 10.1175/1520-0469(1974)031<2099:omnact>2.0.co;2

[jame21128-bib-0090] Cohard, J.‐M. , & Pinty, J.‐P. (2000). A comprehensive two‐moment warm microphysical bulk scheme. 1. Description and tests. Quarterly Journal of the Royal Meteorological Society, 126, 1815–1842. 10.1002/qj.49712656613

[jame21128-bib-0091] Connolly, P. , Emersic, C. , & Field, P. (2012). A laboratory investigation into the aggregation efficiency of small ice crystals. Atmospheric Chemistry and Physics, 12, 2055–2076. 10.5194/acp-12-2055-2012

[jame21128-bib-0092] Connolly, P. J. , Choularton, T. W. , Gallagher, M. W. , Bower, K. N. , Flynn, M. J. , & Whiteway, J. A. (2006). Cloud‐resolving simulations of intense tropical Hector thunderstorms: Implications for aerosol‐cloud interactions. Quarterly Journal of the Royal Meteorological Society, 132, 3079–3106. 10.1256/qj.05.86

[jame21128-bib-0093] Connolly, P. J. , Saunders, C. P. R. , Gallagher, M. W. , Bower, K. N. , Flynn, M. J. , Choularton, T. W. , Whiteway, J. , & Lawson, P. (2005). Aircraft observations of the influence of electric fields on the aggregation of ice crystals. Quarterly Journal of the Royal Meteorological Society, 131, 1695–1712. 10.1256/qj.03.217

[jame21128-bib-0094] Cooper, W. A. (1989). Effects of variable droplet growth histories on stochastic condensation theory. Journal of the Atmospheric Sciences, 49, 1301–1311. 10.1175/1520-0469(1989)046<1301:EOVDGH>2.0.CO;2

[jame21128-bib-0095] Cotton, W. R. (1972). Numerical simulation of precipitation development in supercooled cumuli—Part II. Monthly Weather Review, 100, 764–784. 10.1175/1520-0493(1972)100<0764:nsopdi>2.3.co;2

[jame21128-bib-0096] Cotton, W. R. , Stephens, M. A. , Nehrkorn, T. , & Tripoli, G. J. (1982). The Colorado State University three‐dimensional cloud/mesoscale model—1982. Part II: An ice phase parameterization. Journal de Recherches Atmospheriques, 16, 295–320.

[jame21128-bib-0097] Cotton, W. R. , Tripoli, G. J. , Rauber, R. M. , & Mulvhill, E. A. (1986). Numerical simulation of the effects of varying ice crystal nucleation rates and aggregation processes on orographic snowfall. Journal of Climate and Applied Meteorology, 25, 1658–1680. 10.1175/1520-0450(1986)025<1658:nsoteo>2.0.co;2

[jame21128-bib-0098] David, R. O. , Marcolli, C. , Fahrni, J. , Qiu, Y. , Perez Sirkin, Y. A. , Molinero, V. , Mahrt, F. , Brühwiler, D. , Lohmann, U. , & Kanji, Z. A. (2019). Pore condensation and freezing is responsible for ice formation below water saturation for porous particles. Proceedings of the National Academy of Sciences, 116(17), 8184–8189. 10.1073/pnas.1813647116 PMC6486705 30948638

[jame21128-bib-0099] Davison, C. R. , MacLeod, J. D. , & Strapp, J. W. (2009). Naturally aspirating isokinetic total water content probe: Evaporator design and testing, First AIAA Atmospheric and Space Environments Conf., San Antonio, TX, AIAA‐2009‐3861.

[jame21128-bib-0100] Dawson, D. T. , Xue, M. , Milbrandt, J. A. , & Shapiro, A. (2015). Sensitivity of real‐data simulations of the 3 May 1999 Oklahoma City tornadic superce3ll and associated tornadoes to multimoment microphysics. Part I: Storm‐ and tornado‐scale numerical forecasts. Monthly Weather Review, 143, 2241–2265. 10.1175/MWR-D-14-00279.1

[jame21128-bib-0101] de Rooy, W. C. , Bechtold, P. , Frohlich, K. , Hohenegger, C. , Jonker, H. , Mironov, D. , Siebesma, A. P. , Teixeira, J. , & Yano, J. I. (2013). Entrainment and detrainment in cumulus convection: An overview. Quarterly Journal of the Royal Meteorological Society, 139, 1–19. 10.1002/qj.1959

[jame21128-bib-0102] Dearden, C. , Vaughan, G. , Tsai, T. , & Chen, J. (2016). Exploring the diabatic role of ice microphysical processes in two North Atlantic summer cyclones. Monthly Weather Review, 144, 1249–1272. 10.1175/MWR-D-15-0253.1

[jame21128-bib-0104] DeMott, P. J. , Möhler, O. , Cziczo, D. J. , Hiranuma, N. , Petters, M. D. , Petters, S. S. , Belosi, F. , Bingemer, H. , Brooks, S. D. , Budke, C. , Burkert‐Kohn, M. , Collier, K. N. , Danielczok, A. , Eppers, O. , Felgitsch, L. , Garimella, S. , Grothe, H. , Herenz, P. , Hill, T. C. J. , Höhler, K. , Kanji, Z. A. , Kiselev, A. , Koop, T. , Kristensen, T. B. , Krüger, K. , Kulkarni, G. , Levin, E. J. T. , Murray, B. J. , Nicosia, A. , O'Sullivan, D. , Peckhaus, A. , Polen, M. J. , Price, H. C. , Reicher, N. , Rothenberg, D. A. , Rudich, Y. , Santachiara, G. , Schiebel, T. , Schrod, J. , Seifried, T. M. , Stratmann, F. , Sullivan, R. C. , Suski, K. J. , Szakáll, M. , Taylor, H. P. , Ullrich, R. , Vergara‐Temprado, J. , Wagner, R. , Whale, T. F. , Weber, D. , Welti, A. , Wilson, T. W. , Wolf, M. J. , & Zenker, J. (2018). The Fifth International Workshop on Ice Nucleation phase 2 (FIN‐02): Laboratory intercomparison of ice nucleation measurements. Atmospheric Measurement Techniques, 11, 6231–6257. 10.5194/amt-11-6231-2018

[jame21128-bib-0105] DeMott, P. J. , Prenni, A. J. , Liu, X. , Kreidenweis, S. M. , Petters, M. D. , Twohy, C. H. , Richardson, M. S. , Eidhammer, T. , & Rogers, D. C. (2010). Predicting global atmospheric ice nuclei distributions and their impacts on climate. Proceedings of the National Academy of Sciences, 107(25), 11,217–11,222. 10.1073/pnas.0910818107 PMC2895116 20534566

[jame21128-bib-0106] Desai, N. , Chandrakar, K. K. , Chang, K. , Cantrell, W. , & Shaw, R. A. (2018). Influence of microphysical variability on stochastic condensation in a turbulent laboratory cloud. Journal of the Atmospheric Sciences, 75(1), 189–201. 10.1175/jas-d-17-0158.1

[jame21128-bib-0107] Desai, N. , Chandrakar, K. K. , Kinney, G. , Cantrell, W. , & Shaw, R. A. (2019). Aerosol‐mediated glaciation of mixed‐phase clouds: Steady‐state laboratory measurements. Geophysical Research Letters, 46, 9154–9162. 10.1029/2019GL083503

[jame21128-bib-0108] DeVille, R. E. , Riemer, N. , & West, M. (2011). Weighted flow algorithms (WFA) for stochastic particle coagulation. Journal of Computational Physics, 230, 8427–8451. 10.1016/j.jcp.2011.07.027

[jame21128-bib-0109] Di Noia, A. , Hasekamp, O. P. , van Diedenhoven, B. , & Zhang, Z. (2019). Retrieval of liquid water cloud properties from POLDER‐3 measurements using a neural network ensemble approach. Atmospheric Measurement Techniques, 12, 1697–1716. 10.5194/amt-12-1697-2019

[jame21128-bib-0110] Dolan, B. , & Rutledge, S. A. (2009). A theory‐based hydrometeor identification algorithm for X‐band polarimetric radars. Journal of Atmospheric and Oceanic Technology, 26, 2071–2088. 10.1175/2009jtecha1208.1

[jame21128-bib-0111] Dong, Y. Y. , Oraltay, R. G. , & Hallett, J. (1994). Ice particle generation during evaporation. Atmospheric Research, 32(1–4), 45–53. 10.1016/0169-8095(94)90050-7

[jame21128-bib-0112] Drake, R. L. (1972). The scalar transport equation of coalescence theory: Moments and kernels. Journal of the Atmospheric Sciences, 29, 537–547. 10.1175/1520-0469(1972)029<0537:tsteoc>2.0.co;2

[jame21128-bib-0113] Dye, J. E. , & Hobbs, P. V. (1968). The influence of environmental parameters on the freezing and fragmentation of suspended water drops. Journal of the Atmospheric Sciences, 25(1), 82–96. 10.1175/1520-0469(1968)025<0082:TIOEPO>2.0.CO;2

[jame21128-bib-0114] Dziekan, P. , & Pawlowska, H. (2017). Stochastic coalescence in Lagrangian cloud microphysics. Atmospheric Chemistry and Physics, 17(2), 13,509–13,520. 10.5194/acp-17-13509-2017

[jame21128-bib-0115] Dziekan, P. , Waruszewski, M. , & Pawlowska, H. (2019). University of Warsaw Lagrangian cloud model (UWLCM) 1.0: A modern large‐eddy simulation tool for warm cloud modeling with Lagrangian microphysics. Geoscientific Model Development Discussion, 12, 1–26. 10.5194/gmd-2018-281 https://www.geosci-model-dev-discuss.net/gmd-2018-281/

[jame21128-bib-0116] Egan, B. A. , & Mahoney, J. R. (1972). Numerical modeling of advection and diffusion of urban area source pollutants. Journal of Applied Meteorology, 11, 312–322. 10.1175/1520-0450(1972)011<0312:nmoaad>2.0.co;2

[jame21128-bib-0117] Einstein, A. (1921). Geometrie und Erfahrung In Geometrie und Erfahrung, 2–20. Berlin, Heidelberg: Springer 10.1007/978-3-642-49903-6_1

[jame21128-bib-0118] Eliasson, S. , Karlsson, K. G. , van Meijgaard, E. , Meirink, J. F. , Stengel, M. , & Willén, U. (2019). The Cloud_cci simulator v1.0 for the Cloud_cci climate data record and its application to a global and a regional climate model. Geoscientific Model Development, 12, 829–847. 10.5194/gmd-12-829-2019

[jame21128-bib-0119] Emersic, C. , & Connolly, P. J. (2017). Microscopic observations of riming on an ice surface using high speed video. Atmospheric Research, 185, 65–72. 10.1016/j.atmosres.2016.10.014

[jame21128-bib-0121] Fan, J. , Leung, L. R. , Li, Z. , Morrison, H. , Qian, Y. , Zhou, Y. , & Chen, H. (2012). Aerosol impacts on clouds and precipitation in southeast China—Results from bin and bulk microphysics for the 2008 AMF‐China field campaign. Journal of Geophysical Research, 117, D00K36 10.1029/2011JD016537

[jame21128-bib-0122] Feingold, G. , Cotton, W. R. , Kreidenweis, S. M. , & Davis, J. T. (1999). The impact of giant cloud condensation nuclei on drizzle formation in stratocumulus: Implications for cloud radiative properties. Journal of the Atmospheric Sciences, 56, 4100–4117. 10.1175/1520-0469(1999)056<4100:tiogcc>2.0.co;2

[jame21128-bib-0123] Feingold, G. , Kreindenweis, S. M. , Stevens, B. , & Cotton, W. R. (1996). Numerical simulations of stratocumulus processing of cloud condensation nuclei through collision‐coalescence. Journal of Geophysical Research, 101, 21,391–21,402. 10.1029/96JD01552

[jame21128-bib-0124] Feingold, G. , Tzivion, T. , & Leviv, S. (1988). Evolution of raindrop spectra. Part I: Solution to the stochastic collection/breakup equation using the method of moments. Journal of the Atmospheric Sciences, 45, 3387–3399. 10.1175/1520-0469(1988)045<3387:eorspi>2.0.co;2

[jame21128-bib-0125] Feingold, G. , Walko, R. L. , Stevens, B. , & Cotton, W. R. (1998). Simulations of marine stratocumulus using a new microphysical parameterization scheme. Atmospheric Research, 47–48, 505–528. 10.1016/s0169-8095(98)00058-1

[jame21128-bib-0126] Ferrier, B. S. (1994). A double‐moment multiple‐phase four‐class bulk ice scheme. Part I: Description. Journal of the Atmospheric Sciences, 51, 249–280. 10.1175/1520-0469(1994)051<0249:admmpf>2.0.co;2

[jame21128-bib-0127] Field, P. (1999). Aircraft observations of ice crystal evolution in an altostratus cloud. Journal of the Atmospheric Sciences, 56, 1925–1941. 10.1175/1520-0469(1999)056<1925:AOOICE>2.0.CO;2

[jame21128-bib-0128] Field, P. R. , Brown, P. R. A. , Lawson, R. P. , & Lloyd, G. (2017). Secondary ice production—Current state of the science and recommendations for the future. Meteorological Monographs, 58, 7.1–7.20. 10.1175/AMSMONOGRAPHS-D-16-0014.1

[jame21128-bib-0129] Field, P. R. , Heymsfield, A. J. , Shipway, B. J. , DeMott, P. J. , Pratt, K. A. , Rogers, D. C. , Stith, J. , & Prather, K. A. (2012). Ice in clouds experiment–layer clouds. Part II: Testing characteristics of heterogeneous ice formation in lee wave clouds. Journal of the Atmospheric Sciences, 69, 1066–1079. 10.1175/JAS-D-11-026.1

[jame21128-bib-0130] Findeisen, W. (1940). Über die Entstehung der Gewitterelek‐trizität (On the origin of lightning electricity). Meteorologische Zeitschrift, 6, 201–221.

[jame21128-bib-0131] Findeisen, W. , & Findeisen, E. (1943). Untersuchungen über die Eissplitterbildung an Reifschichten (Eio Beitrag zur Frage der Ents tebung der Gewitterelekfrizität und zur Mikrostruktur der Cumulonirnben). Investigations on the ice splinters formation on rime layers (A contribution to the problem of the origin of storm electricity and to the microstructure of cumulonimbus). Meteorologische Zeitschrift, 60, 145–154.

[jame21128-bib-0132] Firda, J. M. , Sekelsky, S. M. , & McIntosh, R. E. (1999). Application of dual‐frequency millimeter‐wave Doppler spectra for the retrieval of drop size distributions and vertical air motion in rain. Journal of Atmospheric and Oceanic Technology, 16, 216–236. 10.1175/1520-0426(1999)016<0216:AODFMW>2.0.CO;2

[jame21128-bib-0133] Flack, D. L. A. , Gray, S. L. , & Plant, R. S. (2019). A simple ensemble approach for more robust process‐based sensitivity analysis of case studies in convection‐permitting models. Quarterly Journal of the Royal Meteorological Society, 145, 3089–3101. 10.1002/qj3606

[jame21128-bib-0134] Fletcher, N. H. (1958). Size effect in heterogeneous nucleation. The Journal of Chemical Physics, 29(3), 572–576. 10.1063/1.1744540

[jame21128-bib-0135] Fletcher, N. H. (1962). The physics of rainclouds, 390 pp. Cambridge, U.K.: Cambridge University Press.

[jame21128-bib-0136] Forbes, R. , Tompkins, A. M. , & Untch, A. (2011). A new prognostic bulk microphysics scheme for the IFS In ECMWF Technical Memorandum (No. 649) 1–28 pp. Reading, U.K.: ECMWF 10.21957/bf6vjvxk, https://www.ecmwf.int/node/9441

[jame21128-bib-0137] Fovell, R. G. , & Ogura, Y. (1988). Numerical simulation of a midlatitude squall line in two dimensions. Journal of the Atmospheric Sciences, 45, 3846–3879. 10.1175/1520-0469(1988)045<3846:nsoams>2.0.co;2

[jame21128-bib-0138] Franks, S. W. , & Beven, K. J. (1997). Bayesian estimation of uncertainty in land surface‐atmosphere flux predictions. Journal of Geophysical Research, 102, 23,991–23,999. 10.1029/97JD02011 11541236

[jame21128-bib-0140] Fridlind, A. M. , Li, X. , Wu, D. , van Lier‐Walqui, M. , Ackerman, A. S. , Tao, W.‐K. , McFarquhar, G. M. , Wu, W. , Dong, X. , Wang, J. , Ryzhkov, A. , Zhang, P. , Poellot, M. R. , Neumann, A. , & Tomlinson, J. M. (2017). Derivation of aerosol profiles for MC3E convection studies and use in simulations of the 20 May squall line case. Atmospheric Chemistry and Physics, 17, 5947–5972. 10.5194/acp-17-5947-2017

[jame21128-bib-0141] Fridlind, A. M. , van Diedenhoven, B. , Ackerman, A. S. , Avramov, A. , Mrowiec, A. , Morrison, H. , Zuidema, P. , & Shupe, M. D. (2012). A FIRE‐ACE/SHEBA case study of mixed‐phase Arctic boundary‐layer clouds: Entrainment rate limitations on rapid primary ice nucleation processes. Journal of the Atmospheric Sciences, 69, 365–389. 10.1175/JAS-D-11-052.1

[jame21128-bib-0142] Fridlind, A. M. , van Lier‐Walqui, M. , Collis, S. , Giangrande, S. E. , Jackson, R. C. , Li, X. , Matsui, T. , Orville, R. , Picel, M. H. , Rosenfeld, D. , Ryzhkov, A. , Weitz, R. , & Zhang, P. (2019). Use of polarimetric radar measurements to constrain simulated convective cell evolution: A pilot study with Lagrangian tracking. Atmospheric Measurement Techniques, 12, 2979–3000. 10.5194/amt-12-2979-2019

[jame21128-bib-0143] Friedman, J. H. (1991). Multivariate adaptive regression splines (with discussion). Annals of Statistics, 19, 1–141. 10.1214/aos/1176347963

[jame21128-bib-0144] Friedman, J. H. , & Roosen, C. B. (1995). An introduction to multivariate adaptive regression splines. Statistical Methods in Medical Research, 4, 197–217. 10.1177/096228029500400303 8548103

[jame21128-bib-0145] Fugal, J. P. , & Shaw, R. A. (2009). Cloud particle size distributions measured with an airborne digital in‐line holographic instrument. Atmospheric Measurement Techniques, 2(1), 259–271. 10.5194/amt-2-259-2009

[jame21128-bib-0146] Fukuta, N. (1969). Experimental studies on the growth of small ice crystals. Journal of the Atmospheric Sciences, 26, 522–531. 10.1175/1520-0469(1969)026,0522:ESOTGO.2.0.CO;2

[jame21128-bib-0147] Fulton, C. , Salazar, J. L. , Zhang, Y. , Zhang, G. , Kelly, R. , Meier, J. , McCord, M. , Schmidt, D. , Byrd, A. D. , Bhowmik, L. M. , Karimkashi, S. , Zrnic, D. S. , Doviak, R. J. , Zahrai, A. , Yeary, M. , & Palmer, R. D. (2017). Cylindrical polarimetric phased array radar: Beamforming and calibration for weather applications. IEEE Transactions on Geoscience, 55, 2827–2841. 10.1109/tgrs.2017.2655023

[jame21128-bib-0148] Gagin, A. (1972). Effect of supersaturation on the ice crystal production by natural aerosols. Journal de Recherches Atmospheriques, 6, 175–185.

[jame21128-bib-0149] Gagin, A. , & Nozyce, N. (1984). The nucleation of ice crystals during the freezing of large supercooled drops. Journal de Recherches Atmospheriques, 18, 119–129.

[jame21128-bib-0150] Gao, S. , Ran, L. , & Li, X. (2006). Impacts of ice microphysics on rainfall and thermodynamic processes in the tropical deep convective regime: A 2D cloud‐resolving modeling study. Monthly Weather Review, 134, 3015–3024. 10.1175/mwr3220.1

[jame21128-bib-0151] Garimella, S. , Rothenberg, D. A. , Wolf, M. J. , Wang, C. , Rösch, M. , & Cziczo, D. J. (2018). How uncertainty in field measurements of ice nucleating particles influences modeled cloud forcing. Journal of the Atmospheric Sciences, 75 10.1175/JAS-D-17-0089.1

[jame21128-bib-0152] Garrett, T. J. (2019). Analytical solutions for precipitation size distributions at steady state. Journal of the Atmospheric Sciences, 76, 1031–1037. 10.1175/JAS-D-18-0309.1

[jame21128-bib-0153] Garrett, T. J. , Fallgatter, C. , Shkurko, K. , & Howlett, D. (2012). Fall speed measurement and high‐resolution multi‐angle photography of hydrometeors in free fall. Atmospheric Measurement Techniques, 5, 2625–2633. 10.5194/amt-5-2625-2012

[jame21128-bib-0154] Gaussiat, N. , Sauvageot, H. , & Illingworth, A. J. (2003). Cloud liquid water and ice content retrieved by multiwavelength radar. Journal of Atmospheric and Oceanic Technology, 20, 1264–1275. 10.1175/1520-0426(2003)020<1264:clwaic>2.0.co;2

[jame21128-bib-0155] Gelman, A. , & Shaliz, C. R. (2013). Philosophy and the practice of Bayesian statistics. The British Journal of Mathematical and Statistical Psychology, 66, 8–38. 10.1111/j.2044-8317.2011.02037.x 22364575 PMC4476974

[jame21128-bib-0156] Gentine, P. , Pritchard, M. , Rasp, S. , Reinaudi, G. , & Yacalis, G. (2018). Could machine learning break the convection parameterization deadlock? Geophysical Research Letters, 45, 5742–5751. 10.1029/2018GL078202

[jame21128-bib-0157] Geresdi, I. (1998). Idealized simulation of the Colorado hailstorm case: Comparison of bulk and detailed microphysics. Atmospheric Research, 45, 237–252. 10.1016/s0169-8095(97)00079-3

[jame21128-bib-0158] Gettelman, A. , Liu, X. , Barahona, D. , Lohmann, U. , & Chen, C. (2012). Climate impacts of ice nucleation. Journal of Geophysical Research, 117, D20201 10.1029/2012JD017950

[jame21128-bib-0159] Gettelman, A. , & Morrison, H. (2015). Advanced two‐moment bulk microphysics for global models. Part I: Off‐line tests and comparison with other schemes. Journal of Climate, 28, 1268–1287. 10.1175/jcli-d-14-00102.1

[jame21128-bib-0160] Gettelman, A. , Morrison, H. , Terai, C. R. , & Wood, R. (2013). Microphysical process rates and global aerosol‐cloud interactions. Atmospheric Chemistry and Physics, 13, 9855–9867. 10.5194/acp-13-9855-2013

[jame21128-bib-0161] Ghan, S. J. , Leung, L. R. , & Easter, R. C. (1997). Prediction of cloud droplet number in a general circulation model. Journal of Geophysical Research, 102, 21,777–21,794. 10.1029/97JD01810

[jame21128-bib-0162] Giangrande, S. E. , & Ryzhkov, A. V. (2008). Estimation of rainfall based on the results of polarimetric echo classification. Journal of Applied Meteorology and Climatology, 47, 2445–2462. 10.1175/2008jamc1753.1

[jame21128-bib-0163] Gierens, K. M. , Monier, M. , & Gayet, J. F. (2003). The deposition coefficient and its role for cirrus. Journal of Geophysical Research, 108, 4069 10.1029/2001JD001558

[jame21128-bib-0164] Gillepsie, D. T. (1972). The stochastic coalescence model for cloud droplet growth. Journal of the Atmospheric Sciences, 29, 1492–1510. 10.1175/1520-0469(1972)029<1496:tscmfc>2.0.co;2

[jame21128-bib-0166] Grabowski, W. W. (2007). Representation of turbulent mixing and buoyancy reversal in bulk cloud models. Journal of the Atmospheric Sciences, 64, 3666–3680. 10.1175/JAS4047.1

[jame21128-bib-0167] Grabowski, W. W. (2020). Comparison of Eulerian bin and Lagrangian particle‐based schemes in simulations of Pi Chamber dynamics and microphysics. Journal of the Atmospheric Sciences, 77, 1151–1165. 10.1175/jas-d-19-0216.1

[jame21128-bib-0168] Grabowski, W. W. , & Abade, G. C. (2017). Broadening of cloud droplet spectra through eddy hopping: Turbulent adiabatic parcel simulations. Journal of the Atmospheric Sciences, 74, 1485–1493. 10.1175/jas-d-17-0043.1

[jame21128-bib-0169] Grabowski, W. W. , Dziekan, P. , & Pawlowska, H. (2018). Lagrangian condensation microphysics with Twomey CCN activation. Geoscientific Model Development, 11, 103–120. 10.5194/gmd-11-103-2018

[jame21128-bib-0170] Grabowski, W. W. , Morrison, H. , Shima, S. I. , Abade, G. , Pawlowska, H. , & Dziekan, P. (2019). Modeling of cloud microphysics: Can we do better? Bulletin of the American Meteorological Society, 100, 655–672. 10.1175/bams-d-18-0005.1

[jame21128-bib-0171] Gross, M. , Wan, H. , Rasch, P. J. , Caldwell, P. M. , Williamson, D. L. , Klocke, D. , Jablonowski, C. , Thatcher, D. R. , Wood, N. , Cullen, M. , Beare, B. , Willett, M. , Lemarié, F. , Blayo, E. , Malardel, S. , Termonia, P. , Gassmann, A. , Lauritzen, P. H. , Johansen, H. , Zarzycki, C. M. , Sakaguchi, K. , & Leung, R. (2018). Physics‐dynamics coupling in weather, climate and Earth system models: Challenges and recent progress. Monthly Weather Review, 146, 3505–3544. 10.1175/mwr-d-17-0345.1

[jame21128-bib-0172] Grosvenor, D. P. , Sourdeval, O. , Zuidema, P. , Ackerman, A. , Alexandrov, M. D. , Bennartz, R. , Boers, R. , Cairns, B. , Chiu, J. C. , Christensen, M. , Deneke, H. , Diamond, M. , Feingold, G. , Fridlind, A. , Hünerbein, A. , Knist, C. , Kollias, P. , Marshak, A. , McCoy, D. , Merk, D. , Painemal, D. , Rausch, J. , Rosenfeld, D. , Russchenberg, H. , Seifert, P. , Sinclair, K. , Stier, P. , van Diedenhoven, B. , Wendisch, M. , Werner, F. , Wood, R. , Zhang, Z. , & Quaas, J. (2018). Remote sensing of cloud droplet number concentration: Review of current and perspectives for new approaches. Reviews of Geophysics, 56, 409–453. 10.1029/2017RG000593 30148283 PMC6099364

[jame21128-bib-0173] Haddad, Z. S. , Sy, O. O. , Stephens, G. L. , van den Heever, S. C. , & Posselt, D. J. (2018). Atmospheric remote sensing with convoys of miniature radars, Proc. SPIE 10776, Remote Sensing of the Atmosphere, Clouds, and Precipitation VII, 1077601 10.1117/12.2500285

[jame21128-bib-0174] Hall, W. D. (1980). A detailed microphysical model within a two‐dimensional dynamic framework: Model description and preliminary results. Journal of the Atmospheric Sciences, 37, 2486–2507. 10.1175/1520-0469(1980)037<2486:admmwa>2.0.co;2

[jame21128-bib-0176] Hallett, J. (1968). Nucleation and growth of ice crystals in water and biological systems In Low temperature biology of foodstuffs (pp. 23–52). New York: Pergamon Press.

[jame21128-bib-0177] Hallett, J. , & Mossop, S. C. (1974). Production of secondary ice particles during the riming process. Nature, 249(5452), 26–28. 10.1038/249026a0

[jame21128-bib-0178] Hallett, J. , Sax, R. I. , Lamb, D. , & Murty, A. S. R. (1978). Aircraft measurements of ice in Florida cumuli. Quarterly Journal of the Royal Meteorological Society, 104(441), 631–651. 10.1002/qj.49710444108

[jame21128-bib-0179] Ham, F. (1959). Shape‐preserving solutions of the time‐dependent diffusion equation. Quarterly of Applied Mathematics, 17, 137–145. 10.1090/qam/108196

[jame21128-bib-0180] Hardy, K. R. (1963). The development of raindrop‐size distribution and implications related to the physics of precipitation. Journal of the Atmospheric Sciences, 20, 299–312. 10.1175/1520-0469(1963)020<0299:tdorsd>2.0.co;2

[jame21128-bib-0181] Harrington, J. Y. , Moyle, A. , Hanson, L. E. , & Morrison, H. (2019). On calculating deposition coefficients and aspect‐ratio evolution in approximate models of ice crystal vapor growth. Journal of the Atmospheric Sciences, 76, 1609–1625. 10.1175/jas-d-18-0319.1

[jame21128-bib-0182] Harrington, J. Y. , Sulia, K. , & Morrison, H. (2013). A method for adaptive habit prediction in bulk microphysical models. Part I: Theoretical development. Journal of the Atmospheric Sciences, 70, 349–364. 10.1175/jas-d-12-040.1

[jame21128-bib-0183] Harris‐Hobbs, R. L. , & Cooper, W. A. (1987). Field evidence supporting quantitative predictions of secondary ice production rates. Journal of the Atmospheric Sciences, 44(7), 1071–1082. 10.1175/1520-0469(1987)044<1071:FESQPO>2.0.CO;2

[jame21128-bib-0184] Harrison, A. D. , Lever, K. , Sanchez‐Marroquin, A. , Holden, M. A. , Whale, T. F. , Tarn, M. D. , McQuaid, J. B. , & Murray, B. J. (2019). The ice‐nucleating ability of quartz immersed in water and its atmospheric importance compared to K‐feldspar. Atmospheric Chemistry and Physics, 19, 11,343–11,361. 10.5194/acp-19-11343-2019

[jame21128-bib-0185] Hashino, T. , & Tripoli, G. J. (2007). The spectral ice habit prediction system (SHIPS). Part I: Model description and simulation of the vapor deposition process. Journal of the Atmospheric Sciences, 64, 2210–2237. 10.1175/jas3963.1

[jame21128-bib-0186] He, F. , Posselt, D. J. , Narisetty, N. N. , Zarzycki, C. M. , & Nair, V. N. (2018). Application of multivariate sensitivity analysis techniques to AGCM‐simulated tropical cyclones. Monthly Weather Review, 146, 2065–2088. 10.1175/mwr-d-17-0265.1

[jame21128-bib-0187] Heymsfield, A. J. , & Donner, L. J. (1990). A scheme for parameterizing ice‐cloud water content in general circulation models. Journal of the Atmospheric Sciences, 47, 1865–1877. 10.1175/1520-0469(1990)047<1865:asfpic>2.0.co;2

[jame21128-bib-0188] Hindman, E. E. II , & Johnson, D. B. (1970). Numerical simulation of ice hydrometeor development In Preprints—Conference on Cloud Physics (pp. 63–64). Boston, Mass: American Meteorological Society, Boston, Mass.

[jame21128-bib-0189] Hindman, E. E. II , & Johnson, D. B. (1972). Numerical simulation of ice particle growth in a cloud of supercooled water droplets. Journal of the Atmospheric Sciences, 29, 1313–1321. 10.1175/1520-0469(1972)029<1313:nsoipg>2.0.co;2

[jame21128-bib-0190] Hiranuma, N. , Paukert, M. , Steinke, I. , Zhang, K. , Kulkarni, G. , Hoose, C. , Schnaiter, M. , Saathoff, H. , & Möhler, O. (2014). A comprehensive parameterization of heterogeneous ice nucleation of dust surrogate: Laboratory study with hematite particles and its application to atmospheric models. Atmospheric Chemistry and Physics, 14, 13,145–13,158. 10.5194/acp-14-13145-2014

[jame21128-bib-0191] Hobbs, P. V. (1969). Ice multiplication in clouds. Journal of the Atmospheric Sciences, 26(2), 315–318. 10.1175/1520-0469(1969)026<0315:IMIC>2.0.CO;2

[jame21128-bib-0193] Hobbs, P. V. , & Burrows, D. A. (1966). The electrification of an ice sphere moving through natural clouds. Journal of the Atmospheric Sciences, 23(6), 757–763. 10.1175/1520-0469(1966)023<0757:TEOAIS>2.0.CO;2

[jame21128-bib-0195] Hobbs, P. V. , & Farber, R. (1972). Fragmentation of ice particles in clouds. Journal de Recherches Atmospheriques, 6, 245–258.

[jame21128-bib-0196] Hobbs, P. V. , & Rangno, A. L. (1985). Ice particle concentrations in clouds. Journal of the Atmospheric Sciences, 42(23), 2523–2549. 10.1175/1520-0469(1985)042<2523:IPCIC>2.0.CO;2

[jame21128-bib-0197] Hobbs, P. V. , & Rangno, A. L. (1989). Rapid development of high ice particle concentrations in small polar maritime cumuliform clouds. Journal of the Atmospheric Sciences, 47(22), 2710–2722. 10.1175/1520-0469(1990)047<2710:RDOHIP>2.0.CO;2

[jame21128-bib-0198] Hofer, S. , Tedstone, A. J. , Fettweis, X. , & Bamber, J. L. (2019). Cloud microphysics and circulation anomalies in future Greenland melt. Nature Climate Change, 9, 523–528. 10.1038/s41558-019-0507-8

[jame21128-bib-0199] Hoffmann, F. , Yamaguchi, T. , & Feingold, G. (2019). Inhomogeneous mixing in Lagrangian cloud models: Effects on the production of precipitation embryos. Journal of the Atmospheric Sciences, 76, 113–133. 10.1175/JAS-D-18-0087.1

[jame21128-bib-0200] Hoge, M. , Wohling, T. , & Nowak, W. (2018). A primer for model selection: The decisive role of model complexity. Water Resources Research, 54, 1688–1715. 10.1002/2017WR021902

[jame21128-bib-0201] Hohenegger, C. , Brockhaus, P. , & Schar, C. (2008). Towards climate simulations at cloud‐resolving scales. Meteorologische Zeitschrift, 17, 383–394. 10.1127/0941-2948/2008/0303

[jame21128-bib-0202] Holl, G. , Eliasson, S. , Mendrok, J. , & Buehler, S. A. (2014). SPARE‐ICE: Synergistic ice water path from passive operational sensors. Journal of Geophysical Research, 119, 1504–1523. 10.1002/2013JD020759

[jame21128-bib-0203] Hong, S. Y. , Dudhia, J. , & Chen, S. H. (2004). A revised approach to ice microphysical processes for the bulk parameterization of clouds and precipitation. Monthly Weather Review, 132, 103–120. 10.1175/1520-0493(2004)132,0103:ARATIM.2.0.CO;2

[jame21128-bib-0204] Hong, S. Y. , & Lim, J. O. J. (2006). The WRF single‐moment 6‐class microphysics scheme (WSM6). Journal of the Korean Meteorological Society, 42, 129–151.

[jame21128-bib-0205] Hoose, C. , Kristjánsson, J. E. , Chen, J. P. , & Hazra, A. (2010). A classical‐theory‐based parameterization of heterogeneous ice nucleation by mineral dust, soot and biological particles in a global climate model. Journal of the Atmospheric Sciences, 67(8), 2483–2503. 10.1175/2010jas3425.1

[jame21128-bib-0206] Hoose, C. , & Möhler, O. (2012). Heterogeneous ice nucleation on atmospheric aerosols: A review of results from laboratory experiments. Atmospheric Chemistry and Physics, 12, 9817–9854. 10.5194/acp-12-9817-2012

[jame21128-bib-0207] Hourdin, F. , Mauritsen, T. , Gettelman, A. , Golaz, J.‐C. , Balaji, V. , Duan, Q. , Folini, D. , Ji, D. , Klocke, D. , Qian, Y. , Rauser, F. , Rio, C. , Tomassini, L. , Watanabe, M. , & Williamson, D. (2017). The art and science of climate model tuning. Bulletin of the American Meteorological Society, 98, 589–602. 10.1175/BAMS-D-15-00135.1

[jame21128-bib-0208] Houze, R. A. Jr. , Hobbs, P. V. , Herzegh, P. H. , & Parsons, D. B. (1979). Size distributions of precipitation particles in frontal clouds. Journal of the Atmospheric Sciences, 36, 156–162. 10.1175/1520-0469(1979)036<0156:sdoppi>2.0.co;2

[jame21128-bib-0210] Hu, Z. , & Srivastava, R. C. (1995). Evolution of raindrop size distribution by coalescence, breakup, and evaporation: Theory and observations. Journal of the Atmospheric Sciences, 52, 1761–1783. 10.1175/1520-0469(1995)052<1761:eorsdb>2.0.co;2

[jame21128-bib-1005] Juisto, J. E. , & Weickmann, H. K. (1973). Types of snowfall. Amer. Meteor. Soc., 54(11), 1148–1162. 10.1175/1520-0477(1973)054<1148:TOS>2.0.CO;2

[jame21128-bib-0211] Igel, A. L. , Igel, M. R. , & van den Heever, S. C. (2015). Make it a double? Sobering results from simulations using single‐moment microphysics schemes. Journal of the Atmospheric Sciences, 72, 910–925. 10.1175/JAS-D-14-0107.1

[jame21128-bib-0212] Illingworth, A. J. , & Caylor, I. J. (1989). Polarization radar estimates of raindrop size spectra and rainfall rates. Journal of Atmospheric and Oceanic Technology, 6, 939–949. 10.1175/1520-0426(1989)006<0939:preors>2.0.co;2

[jame21128-bib-0213] IPCC (2013). Climate change 2013: The physical science basis. Contribution of Working Group I to the Fifth Assessment Report of the Intergovernmental Panel on Climate Change. In Stocker T. F., Qin D., Plattner G.‐K., Tignor M., Allen S. K., Boschung J., Nauels A., Xia Y., Bex V., & Midgley P. M. (Eds.). Cambridge, United Kingdom and New York, NY, USA, 1535 pp: Cambridge University Press 10.1017/CBO9781107415324

[jame21128-bib-0214] Iskandarani, M. , Wang, S. , Srinivasan, A. , Carlisle Thacker, W. , Winokur, J. , & Knio, O. M. (2016). An overview of uncertainty quantification techniques with application to oceanic and oil‐spill simulations. Journal of Geophysical Research: Oceans, 121, 2789–2808. 10.1002/2015JC011366

[jame21128-bib-0215] Isom, B. , Palmer, R. , Kelley, R. , Meier, J. , Bodine, D. , Yeary, M. , Cheong, B.‐L. , Zhang, Y. , Yu, T.‐Y. , & Biggerstaff, M. I. (2013). The atmospheric imaging radar: Simultaneous volumetric observations using a phased array weather radar. Journal of Atmospheric and Oceanic Technology, 30, 655–675. 10.1175/jtech-d-12-00063.1

[jame21128-bib-0217] Jacobson, M. Z. (2011). Numerical solution to drop coalescence/breakup with a volume‐conserving, positive‐definite, and unconditionally stable scheme. Journal of the Atmospheric Sciences, 68, 334–346. 10.1175/2010jas3605.1

[jame21128-bib-0218] Jankov, I. , Berner, J. , Beck, J. , Jiang, H. , Olson, J. B. , Grell, G. , Smirnova, T. G. , Benjamin, S. G. , & Brown, J. M. (2017). A performance comparison between multiphysics and stochastic approaches within a north american rap ensemble. Monthly Weather Review, 145, 1161–1179. 10.1175/mwr-d-16-0160.1

[jame21128-bib-0219] Jarecka, D. , Grabowski, W. W. , Morrison, H. , & Pawlowska, H. (2013). Homogeneity of the subgrid‐scale turbulent mixing in large‐eddy simulation of shallow convection. Journal of the Atmospheric Sciences, 70, 2751–2767. 10.1175/jas-d-13-042.1

[jame21128-bib-0220] Jaruga, A. , & Pawlowska, H. (2018). Libcloudph++ 2.0: Aqueous‐phase chemistry extension of the particle‐based cloud microphysics scheme. Geoscientific Model Development, 11, 3623–3645. 10.5194/gmd-11-3623-2018

[jame21128-bib-0221] Jaynes, E. T. (2003). Probability theory: The logic of science (762 pp). Cambridge, U.K.: Cambridge University Press.

[jame21128-bib-0223] Jensen, A. A. , Harrington, J. Y. , & Morrison, H. (2018). Microphysical characteristics of squall‐line stratiform precipitation and transition zones simulated using an ice particle property‐evolving model. Monthly Weather Review, 146, 723–743. 10.1175/mwr-d-17-0215.1

[jame21128-bib-0224] Jensen, A. A. , Harrington, J. Y. , Morrison, H. , & Milbrandt, J. A. (2017). Predicting ice shape evolution in a bulk microphysics model. Journal of the Atmospheric Sciences, 74, 2081–2104. 10.1175/jas-d-16-0350.1

[jame21128-bib-0225] Jensen, E. , & Pfister, L. (2004). Transport and freeze‐drying in the tropical tropopause layer. Journal of Geophysical Research: Atmospheres, 109, D02207 10.1029/2003JD004022 https://agupubs.onlinelibrary.wiley.com/doi/abs/10.1029/2003JD004022

[jame21128-bib-0226] Jensen, J. B. , Austin, P. H. , Baker, M. B. , & Blyth, A. M. (1985). Turbulent mixing, spectral evolution and dynamics in a warm cumulus cloud. Journal of the Atmospheric Sciences, 42, 173–192. 10.1175/1520-0469(1985)042<0173:tmsead>2.0.co;2

[jame21128-bib-0227] Jensen, J. B. , & Nugent, A. D. (2017). Condensational growth of drops formed on giant sea‐salt aerosol particles. Journal of the Atmospheric Sciences, 74, 679–697. 10.1175/jas-d-15-0370.1

[jame21128-bib-0228] Jensen, M. P. , Petersen, W. A. , Bansemer, A. , Bharadwaj, N. , Carey, L. D. , Cecil, D. J. , Collis, S. M. , Del Genio, A. D. , Dolan, B. , Gerlach, J. , Giangrande, S. E. , Heymsfield, A. , Heymsfield, G. , Kollias, P. , Lang, T. J. , Nesbitt, S. W. , Neumann, A. , Poellot, M. , Rutledge, S. A. , Schwaller, M. , Tokay, A. , Williams, C. R. , Wolff, D. B. , Xie, S. , & Zipser, E. J. (2016). The midlatitude continental convective clouds experiment (MC3E). Bulletin of the American Meteorological Society, 97, 1667–1686. 10.1175/BAMS-D-14-00228.1 32669729 PMC7362300

[jame21128-bib-0229] Johansen, A. , Youdin, A. N. , & Lithwick, Y. (2012). Adding particle collisions to the formation of asteroids and Kuiper belt objects via streaming instabilities. Astronomy and Astrophysics, 537, 17 pp. 10.1051/0004-6361/201117701

[jame21128-bib-0230] Johnson, D. A. , & Hallett, J. (1968). Freezing and shattering of supercooled water drops. Quarterly Journal of the Royal Meteorological Society, 94(402), 468–482. 10.1002/qj.49709440204

[jame21128-bib-0231] Johnson, J. S. , Cui, Z. , Lee, L. A. , Gosling, J. P. , Blyth, A. M. , & Carslaw, K. S. (2015). Evaluating uncertainty in convective cloud microphysics using statistical emulation. Journal of Advances in Modeling Earth Systems, 7, 162–187. 10.1002/2014ms000383

[jame21128-bib-0232] Kachurin, L. G. , & Bekryaev, V. I. (1960). Investigation of the electrification of crystallizing water. Doklady Akademii Nauk SSSR, 130, 57–60.

[jame21128-bib-0233] Kärcher, B. , Abbatt, J. P. D. , Cox, R. A. , Popp, P. J. , & Voigt, C. (2009). Trapping of trace gases by growing ice surfaces including surface‐saturated adsorption. Journal of Geophysical Research, 114, D13306 10.1029/2009JD011857

[jame21128-bib-0234] Karpatne, A. , Atluri, G. , Faghmous, J. H. , Steinbach, M. , Banerjee, A. , Ganguly, A. , Shekhar, S. , Samatova, N. , & Kumar, V. (2017). Theory‐guided data science: A new paradigm for scientific discovery from data. IEEE Transactions on Knowledge and Data Engineering, 29, 2318–2331. 10.1109/TKDE.2017.2720168

[jame21128-bib-0235] Kavetski, D. , Kuczera, G. , & Franks, S. W. (2006). Bayesian analysis of input uncertainty in hydrological modeling: 2. Application. Water Resources Research, 42, W03407 10.1029/2005WR004368

[jame21128-bib-0236] Kendon, E. J. , Roberts, N. M. , Senior, C. A. , & Roberts, M. J. (2012). Realism of rainfall in a very high‐resolution regional climate model. Journal of Climate, 25, 5791–5806. 10.1175/JCLI-D-11-00562.1

[jame21128-bib-0237] Kessler, E. (1969). On the distribution and continuity of water substance in atmospheric circulations In Meteor. Monogr, 32 (Vol. 10), 88 pp. Boston, Mass: American Meteorological Society.

[jame21128-bib-0238] Kessler, E. (1995). On the continuity and distribution of water substance in atmospheric circulations. Atmospheric Research, 38, 109–145. 10.1016/0169-8095(94)00090-z

[jame21128-bib-0239] Kessler, E. , Feteris, P. J. , & Newburg, E. A. (1963). Role of microphysical processes in shaping vertical profiles of precipitation and cloud, Proc. Tenth Wea. Radar Conf., Boston, Amer. Meteor. Soc., 91–97.

[jame21128-bib-0240] Khain, A. , Pokrovsky, A. , Pinsky, M. , Seifert, A. , & Phillips, V. (2004). Simulation of effects of atmospheric aerosols on deep turbulent convective clouds using a spectral microphysics mixed‐phase cumulus cloud model. Part I: Model description and possible applications. Journal of the Atmospheric Sciences, 61(24), 2963–2982. 10.1175/jas-3350.1

[jame21128-bib-0241] Khain, A. P. , Beheng, K. D. , Heymsfield, A. , Korolev, A. , Krichak, S. O. , Levin, Z. , Pinsky, M. , Phillips, V. , Prabhakaran, T. , Teller, A. , van den Heever, S. C. , & Yano, J.‐I. (2015). Representation of microphysical processes in cloud‐resolving models: Spectral (bin) microphysics versus bulk parameterization. Reviews of Geophysics, 53, 247–322. 10.1002/2014RG000468

[jame21128-bib-0242] Khain, A. P. , Benmoshe, N. , & Pokrovsky, A. (2008). Factors determining the impact of aerosols on surface precipitation from clouds: Attempt of classification. Journal of the Atmospheric Sciences, 65, 1721–1748. 10.1175/2007JAS2515.1

[jame21128-bib-0243] Khain, A. P. , & Pinsky, M. (2018). Physical processes in clouds and cloud modeling (626 pp). Cambridge, U.K.: Cambridge University Press.

[jame21128-bib-0244] Khairoutdinov, M. F. , & Kogan, Y. (2000). A new cloud physics parameterization in a large‐eddy simulation model of marine stratocumulus. Monthly Weather Review, 128, 229–243. 10.1175/1520-0493(2000)128<0229:ancppi>2.0.co;2

[jame21128-bib-0245] Kikuchi, K. (1969). Unknown and peculiar shapes of snow crystals observed at Syowa Station, Antarctica. Journal of the Faculty of Science, 3, 99–116.

[jame21128-bib-0246] Kintea, D. M. , Hauk, T. , Roisman, I. V. , & Tropea, C. (2015). Shape evolution of a melting nonspherical particle. Physical Review E, 92, 033012 10.1103/physreve.92.033012 26465561

[jame21128-bib-0247] Kippenberger, M. , Schustger, G. , Lelieveld, J. , & Crowley, J. N. (2019). Trapping of HCL and oxidized, organic trace‐gases in growing ice at temperatures relevant for cirrus clouds. Atmospheric Chemistry and Physics Discussions, 19 10.5194/acp-2019-557

[jame21128-bib-0248] Kiselev, A. , Bachmann, F. , Pedevilla, P. , Cox, S. J. , Michaelides, A. , Gerthsen, D. , & Leisner, T. (2017). Active sites in heterogeneous ice nucleation—The example of K‐rich feldspars. Science, 355, 367–371. 10.1126/science.aai8034 27940582

[jame21128-bib-0250] Kneifel, S. , Kulie, M. , & Bennartz, R. (2011). A triple‐frequency approach to retrieve microphysical snowfall parameters. Journal of Geophysical Research, 116, D11203 10.1029/2010JD015430

[jame21128-bib-0251] Kneifel, S. , von Lerber, A. , Tiira, J. , Moisseev, D. , Kollias, P. , & Leinonen, J. (2015). Observed relations between snowfall microphysics and triple‐frequency radar measurements. Journal of Geophysical Research: Atmospheres, 120, 6034–6055. 10.1002/2015JD023156

[jame21128-bib-0252] Knorr, W. , & Kattge, J. (2005). Inversion of terrestrial ecosystem model parameter values against eddy covariance measurements by Monte Carlo sampling. Global Change Biology, 11, 1333–1351. 10.1111/j.1365-2486.2005.00977

[jame21128-bib-0253] Koenig, L. R. (1963). The glaciating behavior of small cumulonimbus clouds. Journal of the Atmospheric Sciences, 20(1), 29–47. 10.1175/1520-0469(1963)020<0029:TGBOSC>2.0.CO;2

[jame21128-bib-0254] Koenig, L. R. (1965). Drop freezing through drop breakup. Journal of the Atmospheric Sciences, 22(4), 448–451. 10.1175/1520-0469(1965)022<0448:DFTDB>2.0.CO;2

[jame21128-bib-0255] Koenig, L. R. (1971). Numerical modeling of ice deposition. Journal of the Atmospheric Sciences, 28, 226–237. 10.1175/1520-0469(1971)028<0226:nmoid>2.0.co;2

[jame21128-bib-0256] Koenig, L. R. , & Murray, F. W. (1976). Ice‐bearing cumulus cloud evolution: Numerical simulation and general comparison against observations. Journal of Applied Meteorology, 15(7), 747–762. 10.1175/1520-0450(1976)015<0747:ibccen>2.0.co;2

[jame21128-bib-0257] Kogan, Y. (1991). The simulation of a convective cloud in a 3‐d model with explicit microphysics. Part I: Model description and sensitivity experiments. Journal of the Atmospheric Sciences, 48, 1160–1189. 10.1175/1520-0469(1991)048<1160:tsoacc>2.0.co;2

[jame21128-bib-0258] Kogan, Y. L. (2013). A cumulus cloud microphysics parameterization for cloud‐resolving models. Monthly Weather Review, 70, 1423–1436. 10.1175/jas-d-12-0183.1

[jame21128-bib-0259] Kogan, Y. L. , & Belochitski, A. (2012). Parameterization of cloud microphysics based on full integral moments. Journal of the Atmospheric Sciences, 69, 2229–2242. 10.1175/jas-d-11-0268.1

[jame21128-bib-0260] Korolev, A. (2007). Reconstruction of the sizes of spherical particles from their shadow images. Part I: Theoretical considerations. Journal of Atmospheric and Oceanic Technology, 24, 376–389. 10.1175/JTECH1980.1

[jame21128-bib-0261] Korolev, A. , Heckman, I. , Wolde, M. , Ackerman, A. S. , Fridlind, A. M. , Ladino, L. , Lawson, P. , Milbrandt, J. , & Williams, E. (2020). A new look at the environmental conditions favorable to secondary ice production. Atmospheric Chemistry and Physics, 20, 1391–1429. 10.5194/acp-20-1391-2020

[jame21128-bib-0262] Korolev, A. , McFarquhar, G. , Field, P. R. , Franklin, C. , Lawson, P. , Wang, Z. , Williams, E. , Abel, S. J. , Axisa, D. , Borrmann, S. , Crosier, J. , Fugal, J. , Krämer, M. , Lohmann, U. , Schlenczek, O. , Schnaiter, M. , & Wendisch, M. (2017). Mixed‐phase clouds: Progress and challenges. Meteorological Monographs, 58, 5.1–5.50. 10.1175/amsmonographs-d-17-0001.1

[jame21128-bib-0263] Korolev, A. , Pinsky, M. , & Khain, A. (2013). A new mechanism of droplet size distribution broadening during diffusional growth. Journal of the Atmospheric Sciences, 70, 2051–2071. 10.1175/jas-d-12-0182.1

[jame21128-bib-0264] Korolev, A. V. (1995). Effect of supersaturation fluctuations on droplet size spectra formation. Journal of the Atmospheric Sciences, 52, 3620–3634. 10.1175/1520-0469(1995)052<3620:tiosfo>2.0.co;2

[jame21128-bib-0266] Korolev, A. V. , & Isaac, G. A. (2004). Observations of sublimating ice particles in clouds In Proceedings of the 14th International Conference on clouds and precipitation (pp. 808–811). Bologna, Italy: International Commission of Clouds and Precipitation.

[jame21128-bib-0267] Korolev, A. V. , & Mazin, I. P. (2003). Supersaturation of water vapor in clouds. Journal of the Atmospheric Sciences, 60, 2957–2974. 10.1175/1520-0469(2003)060<2957:sowvic>2.0.co;2

[jame21128-bib-0268] Kostinski, A. B. , & Shaw, R. A. (2005). Fluctuations and luck in droplet growth by coalescence. Bulletin of the American Meteorological Society, 86, 235–224. 10.1175/BAMS-86-2-235

[jame21128-bib-0269] Kovetz, A. , & Olund, B. (1969). The effects of coalescence and condensation on rain formation in a cloud of finite vertical extent. Journal of the Atmospheric Sciences, 26, 1060–1065. 10.1175/1520-0469(1969)026<1060:teocac>2.0.co;2

[jame21128-bib-0270] Krueger, S. K. (2000). Cloud system modeling In Randall D. A. (Ed.), General circulation model development (pp. 605–640). San Diego: Academic Press.

[jame21128-bib-0271] Kulkarni, G. , Fan, J. , Comstock, J. M. , Liu, X. , & Ovchinnikov, M. (2012). Laboratory measurements and model sensitivity to studies of dust deposition nucleation. Atmospheric Chemistry and Physics, 12, 7295–7308. 10.5194/acp-12-7295-2012

[jame21128-bib-0272] Kumjian, M. R. (2018). Weather radars In Andronache C. (Ed.), Remote sensing of clouds and precipitation, Chapter 2, (p. 15–63). New York City, New York: Springer.

[jame21128-bib-0273] Kumjian, M. R. , & Lombardo, K. A. (2017). Insights into the evolving microphysical and kinematic structure of northeastern U.S. winter storms from dual‐polarization Doppler radar. Monthly Weather Review, 145, 1033–1061. 10.1175/mwr-d-15-0451.1

[jame21128-bib-0274] Kumjian, M. R. , Martinkus, C. P. , Prat, O. , Collis, S. , van Lier‐Walqui, M. , & Morrison, H. (2019). A moment‐based polarimetric radar forward operator for warm rain microphysics. Journal of Applied Meteorology and Climatology, 58, 113–130. 10.1175/jamc-d-18-0121.1

[jame21128-bib-0275] Kumjian, M. R. , & Prat, O. P. (2014). The impact of raindrop collisional processes on the polarimetric radar variables. Journal of the Atmospheric Sciences, 71, 3052–3067. 10.1175/jas-d-13-0357.1

[jame21128-bib-0276] Kumjian, M. R. , Richardson, Y. , Meyer, T. , Kosiba, K. , & Wurman, J. (2018). Resonance scattering effects in wet hail observed with a dual‐X‐band‐frequency, dual‐polarization Doppler on wheels radar. Journal of Applied Meteorology and Climatology, 57, 2713–2731. 10.1175/jamc-d-17-0362.1

[jame21128-bib-0277] Kumjian, M. R. , & Ryzhkov, A. V. (2010). The impact of evaporation on polarimetric characteristics of rain: Theoretical model and practical implications. Journal of Applied Meteorology and Climatology, 49, 1247–1267. 10.1175/2010jamc2243.1

[jame21128-bib-0278] Kumjian, M. R. , & Ryzhkov, A. V. (2012). The impact of size sorting on the polarimetric radar variables. Journal of the Atmospheric Sciences, 69, 2042–2060. 10.1175/jas-d-11-0125.1

[jame21128-bib-0279] Kunihiro, T. , & Tsumura, K. (2006). Application of the renormalization‐group method to the reduction of transport equations. Journal of Physics A: Mathematical and General, 39(39), 8089 10.1088/0305-4470/39/25/s20

[jame21128-bib-0280] Kuo, T. H. , Murakami, M. , Tajiri, T. , & Orisaka, N. (2019). Cloud condensation nuclei and immersion freezing abilities of Al2O3 and Fe2O3 particles measured with the Meteorological Research Institute's Cloud Simulation Chamber. Journal of the Meteorological Society of Japan, 97(3), 597–614. 10.2151/jmsj.2019-032

[jame21128-bib-0281] Laine, M. , Solonen, A. , Haario, H. , & Jarvinen, H. (2011). Ensemble prediction and parameter estimation system: The method. Quarterly Journal of the Royal Meteorological Society, 138, 289–297. 10.1002/qj.922

[jame21128-bib-0282] Langham, E. J. , & Mason, B. J. (1958). The heterogeneous and homogeneous nucleation of supercooled water. Proceedings of the Royal Society A, 247, 493–504. 10.1098/rspa.1958.0207

[jame21128-bib-0283] Langmuir, I. (1948). The production of rain by a chain reaction in cumulus clouds at temperatures above freezing. Journal of the Atmospheric Sciences, 5, 175–192. 10.1175/1520-0469(1948)005<0175:TPORBA>2.0.CO;2

[jame21128-bib-0284] Larson, V. E. , Golaz, J.‐C. , Jiang, H. , & Cotton, W. R. (2005). Supplying local microphysics parameterizations with information about subgrid variability: Latin hypercube sampling. Journal of the Atmospheric Sciences, 62, 4010–4026. 10.1175/jas3624.1

[jame21128-bib-0285] Larson, V. E. , & Griffin, B. M. (2013). Analytic upscaling of a local microphysics schemes. Part 1: Derivation. Quarterly Journal of the Royal Meteorological Society, 139, 46–57. 10.1002/qj.1967

[jame21128-bib-0286] Lasher‐Trapp, S. G. , Cooper, W. A. , & Blyth, A. M. (2005). Broadening of droplet size distributions from entrainment and mixing in a cumulus cloud. Quarterly Journal of the Royal Meteorological Society, 131, 195–220. 10.1256/qj.03.199

[jame21128-bib-0287] Latham, J. , & Mason, B. J. (1961). Generation of electric charge associated with the formation of soft hail in thunderclouds. Proceedings of the Royal Society A, 260, 237–249. 10.1098/rspa.1961.0052

[jame21128-bib-0288] Latham, J. , & Saunders, C. P. R. (1970). The electrostatic forces on charged ice crystals separated by small distances in an electric field. Quarterly Journal of the Royal Meteorological Society, 96, 266–272. 10.1002/qj.49709640809

[jame21128-bib-0289] Lauber, A. , Kiselev, A. , Pander, T. , Handmann, P. , & Leisner, T. (2018). Secondary ice formation during freezing of levitated droplets. Journal of the Atmospheric Sciences, 75(8), 2815–2826. 10.1175/JAS-D-18-0052.1

[jame21128-bib-0290] Lauritzen, P. H. , & Thuburn, J. (2012). Evaluating advection/transport schemes using interrelated tracers, scatter plots and numerical mixing diagnostics. Quarterly Journal of the Royal Meteorological Society, 138, 906–918. 10.1002/qj.986

[jame21128-bib-0291] Lawson, P. , Gurganus, C. , Woods, S. , & Bruintjes, R. (2017). Aircraft observations of cumulus microphysics ranging from the tropics to didlatitudes: Implications for a “new” secondary ice process. Journal of the Atmospheric Sciences, 74(9), 2899–2920. 10.1175/JAS-D-17-0033.1

[jame21128-bib-0292] Lawson, P. , Woods, S. , Jensen, E. , Erfani, E. , Gurganus, C. , Gallagher, M. , Connolly, P. , Whiteway, J. , Baran, A. J. , May, P. , Heymsfield, A. , Schmitt, C. G. , McFarquhar, G. , Um, J. , Protat, A. , Bailey, M. , Lance, S. , Muehlbauer, A. , Stith, J. , Korolev, A. , Toon, O. B. , & Krämer, M. (2019). A review of ice particle shapes in cirrus formed in situ and in anvils. Journal of Geophysical Research, 124, 10,049–10,090. 10.1029/2018JD030122

[jame21128-bib-0293] Lean, H. W. , Clark, P. A. , Dixon, M. , Roberts, N. M. , Fitch, A. , Forbes, R. , & Halliwell, C. (2008). Characteristics of high‐resolution versions of the Met Office Unified Model for forecasting convection over the United Kingdom. Monthly Weather Review, 136, 3408–3424. 10.1175/2008MWR2332.1

[jame21128-bib-0294] Lebo, Z. J. , Morrison, H. , & Seinfeld, J. H. (2012). Are simulated aerosol effects on deep convective clouds strongly dependent on saturation adjustment? Atmospheric Chemistry and Physics, 12, 9941–9964. 10.5194/acp-12-9941-2012

[jame21128-bib-0295] Lebo, Z. J. , & Seinfeld, J. H. (2011). Theoretical basis for convective invigoration due to increased aerosol concentration. Atmospheric Chemistry and Physics, 11, 5407–5429. 10.5194/acp-11-5407-2011

[jame21128-bib-0296] Lee, H. , Fridlind, A. M. , & Ackerman, A. S. (2019). An evaluation of size‐resolved cloud microphysics scheme numerics for use with radar observations. Part I: Collision‐coalescence. Journal of the Atmospheric Sciences, 76(1), 247–263. 10.1175/JAS-D-18-0174.1

[jame21128-bib-0297] Lehmann, K. , Siebert, H. , & Shaw, R. A. (2009). Homogeneous and inhmogeneous mixing in cumulus clouds: Dependence on local turbulence structure. Journal of the Atmospheric Sciences, 66, 3641–3659. 10.1175/2009jas3012.1

[jame21128-bib-0298] Leinonen, J. , Lebsock, M. D. , Stephens, G. L. , & Suzuki, K. (2016). Improved retrieval of cloud liquid water from CloudSat and MODIS. Journal of Applied Meteorology and Climatology, 55, 1831–1844. 10.1175/JAMC-D-16-0077.1

[jame21128-bib-0299] Li, X. , Tao, W.‐K. , Matsui, T. , Liu, C. , & Masunaga, H. (2010). Improving a spectral bin microphysical scheme using TRMM satellite observations. Quarterly Journal of the Royal Meteorological Society, 136, 382–399. 10.1002/qj.569

[jame21128-bib-0300] Li, X.‐Y. , Brandenburg, A. , Haugen, N. E. L. , & Svensson, G. (2017). Eulerian and Lagrangian approaches to multidimensional condensation and collection. Journal of Advances in Modeling Earth Systems, 9, 1116–1137. 10.1002/2017MS000930

[jame21128-bib-0301] Lim, K.‐S. S. , & Hong, S.‐Y. (2010). Development of an effective double‐moment cloud microphysics scheme with prognostic cloud condensation nuclei (CCN) for weather and climate models. Monthly Weather Review, 138(5), 1587–1612. 10.1175/2009mwr2968.1

[jame21128-bib-0302] Lin, Y. , & Colle, B. A. (2011). A new bulk microphysical scheme that includes riming intensity and temperature‐dependent ice characteristics. Monthly Weather Review, 139, 1013–1035. 10.1175/2010mwr3293.1

[jame21128-bib-0303] Lin, Y.‐L. , Farley, R. D. , & Orville, H. D. (1983). Bulk parameterization of the snow field in a cloud model. Journal of Climate and Applied Meteorology, 22, 1065–1092. 10.1175/1520-0450(1983)022<1065:bpotsf>2.0.co;2

[jame21128-bib-0304] List, R. , Donaldson, N. R. , & Stewart, R. E. (1987). Temporal evolution of drop spectra to collisional equilibrium in steady and pulsating rain. Journal of the Atmospheric Sciences, 44, 362–372. 10.1175/1520-0469(1987)044<0362:teodst>2.0.co;2

[jame21128-bib-0305] List, R. , & McFarquhar, G. M. (1990). The role of breakup and coalescence in the three‐peak equilibrium distribution of raindrops. Journal of the Atmospheric Sciences, 47, 2274–2292. 10.1175/1520-0469(1990)047<2274:trobac>2.0.co;2

[jame21128-bib-0306] Liu, C.‐H. , Moncrieff, M. W. , & Zipser, E. J. (1997). Dynamical influence of microphysics in tropical squall lines: A numerical study. Monthly Weather Review, 125, 2193–2210. 10.1175/1520-0493(1997)125<2193:diomit>2.0.co;2

[jame21128-bib-0307] Liu, H. , & Chandrasekar, V. (2000). Classification of hydrometeors based on polarimetric radar measurements: Development of fuzzy logic and neuro‐fuzzy systems, and in situ verification. Journal of Atmospheric and Oceanic Technology, 17, 140–164. 10.1175/1520-0426(2000)017<0140:COHBOP>2.0.CO;2

[jame21128-bib-0308] Liu, J. Y. , & Orville, H. D. (1969). Numerical modeling of precipitation and cloud shadow effects on mountain‐induced cumuli. Journal of the Atmospheric Sciences, 26, 1283–1298. 10.1175/1520-0469(1969)026<1283:NMOPAC>2.0.CO;2

[jame21128-bib-0309] Liu, Q. , Kogan, Y. L. , Lilly, D. K. , & Khairoutdinov, M. P. (1997). Variational optimization method for calculation of cloud drop growth in an Eulerian drop‐size framework. Journal of the Atmospheric Sciences, 54, 2439–2504. 10.1175/1520-0469(1997)054<2493:VOMFCO>2.0.CO;2

[jame21128-bib-0310] Liu, X. , & Penner, J. E. (2005). Ice nucleation parameterization for global models. Meteorologische Zeitschrift, 14(4), 499–514. 10.1127/0941-2948/2005/0059

[jame21128-bib-0311] Liu, X. , Shi, X. , Zhang, K. , Jensen, E. J. , Gettelman, A. , Barahona, D. , Nenes, A. , & Lawson, P. (2012). Sensitivity studies of dust ice nuclei effect on cirrus clouds with the community atmosphere model CAM5. Atmospheric Chemistry and Physics, 12, 12,061–12,079. 10.5194/acp-12-12061-2012

[jame21128-bib-0312] Liu, Y. , Laiguang, Y. , Weinong, Y. , & Feng, L. (1995). On the size distribution of cloud droplets. Atmospheric Research, 35, 201–216. 10.1016/0169-8095(94)00019-A

[jame21128-bib-0313] Locatelli, J. D. , & Hobbs, P. V. (1974). Fall speeds and masses of solid precipitating particles. Journal of Geophysical Research, 79, 2185–2197. 10.1029/jc079i015p02185

[jame21128-bib-0314] Loftus, A. M. , Cotton, W. R. , & Carrió, G. G. (2014). A triple‐moment hail bulk microphysics scheme. Part I: Description and initial evaluation. Atmospheric Research, 149, 35–57. 10.1016/j.atmosres.2014.05.013

[jame21128-bib-0315] Lohmann, U. , Feichter, J. , Chuang, C. C. , & Penner, J. E. (1999). Prediction of the number of cloud droplets in the ECHAM GCM. Journal of Geophysical Research, 104, 9169–9198. 10.1029/1999JD900046

[jame21128-bib-0316] Long, A. B. (1974). Solutions to the droplet collection equation for polynomial kernels. Journal of the Atmospheric Sciences, 31, 1040–1052. 10.1175/1520-0469(1974)031<1040:sttdce>2.0.co;2

[jame21128-bib-0317] Lord, S. J. , Willoughby, H. E. , & Piotrowicz, J. M. (1984). Role of parameterized ice‐phase microphysics in an axisymmetric, nonhydrostatic tropical cyclone model. Journal of the Atmospheric Sciences, 41, 2836–2848. 10.1175/1520-0469(1984)041<2836:roapip>2.0.co;2

[jame21128-bib-0318] Lou, X. , Shi, Y. , Sun, J. , Xue, L. , Hu, Z. , Fang, W. , & Liu, W. (2012). Cloud‐resolving model for weather modification in China. Chinese Science Bulletin, 57(9), 1055–1061. 10.1007/s11434-011-4934-9

[jame21128-bib-0319] Lovering, K. A. , Bertram, A. K. , & Chou, K. C. (2017). Transient phase of ice observed by sum frequency generation at the water/mineral interface during freezing. Journal of Physical Chemistry Letters, 8(4), 871–875. 10.1021/acs.jpclett.6b02920 28151687

[jame21128-bib-0320] Low, T. B. , & List, R. (1982a). Collision, coalescence and breakup of raindrops. Part I: Experimentally established coalescence efficiencies and fragment size distributions in breakup. Journal of the Atmospheric Sciences, 39, 1591–1606. 10.1175/1520-0469(1982)039<1591:ccabor>2.0.co;2

[jame21128-bib-0321] Low, T. B. , & List, R. (1982b). Collision, coalescence and breakup of raindrops. Part II: Parameterization of fragment size distributions. Journal of the Atmospheric Sciences, 39, 1607–1618. 10.1175/1520-0469(1982)039<1607:ccabor>2.0.co;2

[jame21128-bib-0322] Ludlam, F. H. (1951). The production of showers by the coalescence of cloud droplets. Quarterly Journal of the Royal Meteorological Society, 77, 402–417. 10.1002/qj.49707733306

[jame21128-bib-0323] Luke, E. , & Kollias, P. (2013). Separating cloud and drizzle radar moments during precipitation onset using Doppler spectra. Journal of Atmospheric and Oceanic Technology, 30, 1656–1671. 10.1175/jtech-d-11-00195.1

[jame21128-bib-0324] Macklin, W. C. (1960). The production of ice splinters during riming. Nubila, 3, 30–33.

[jame21128-bib-0325] Magono, C. (1970). On the crystal shape of snow and ice crystals in the cold temperature region: Part I In American Meteorological Society Conference on Cloud Physics (pp. 75–76). Boston, Mass: Ft. Collins, Colorado.

[jame21128-bib-0326] Malkina, A. D. , & Zak, E. G. (1952). Mechanism of freezing of liquid droplets. Transactions of the Central Aerological Observatory (Trudi TsAO), 9, 61–75.

[jame21128-bib-0327] Mansell, E. R. , & Ziegler, C. L. (2013). Aerosol effects on simulated storm electrification and precipitation in a two‐moment bulk microphysics model. Journal of the Atmospheric Sciences, 70, 2032–2050. 10.1175/JAS-D-12-0264.1

[jame21128-bib-0328] Marshall, J. S. , & Palmer, W. M. K. (1948). The distribution of raindrops with size. Journal of Meteorology, 5(4), 165–166. 10.1175/1520-0469(1948)005<0165:tdorws>2.0.co;2

[jame21128-bib-0329] Marzouk, Y. , & Najm, H. N. (2009). Dimensionality reduction and polynomial chaos acceleration of Bayesian inference in inverse problems. Journal of Computational Physics, 228, 1862–1902. 10.1016/j.jcp.2008.11.024

[jame21128-bib-0330] Mason, B. J. , & Maybank, J. (1960). The fragmentation and electrification of freezing water drops. Quarterly Journal of the Royal Meteorological Society, 86, 176–185. 10.1002/qj.49708636806

[jame21128-bib-0331] Mason, B. J. , & Ramanadham, R. (1954). Modification of the size distribution of falling raindrops by coalescence. Quarterly Journal of the Royal Meteorological Society, 80, 388–394. 10.1002/qj.49708034508

[jame21128-bib-0332] Matrosov, S. Y. , Clark, K. , Martner, B. , & Tokay, A. (2002). X‐band polarimetric radar measurements of rainfall. Journal of Applied Meteorology, 41, 941–952. 10.1175/1520-0450(2002)041<0941:xbprmo>2.0.co;2

[jame21128-bib-0333] McCumber, M. , Tao, W.‐K. , Simpson, J. , Penc, R. , & Soong, S.‐T. (1991). Comparison of ice‐phase microphysical parameterization schemes using numerical simulations of tropical convection. Journal of Applied Meteorology, 30, 985–1004. 10.1175/1520-0450-30.7.985

[jame21128-bib-0334] McFarquhar, G. M. (2004). A new representation of collision induced breakup of raindrops and its implications for the shapes of raindrop size distributions. Journal of the Atmospheric Sciences, 61, 777–794. 10.1175/1520-0469(2004)061<0777:anrocb>2.0.co;2

[jame21128-bib-0335] McFarquhar, G. M. , Baumgardner, D. , Bansemer, A. , Abel, S. J. , Crosier, J. , French, J. , Rosenberg, P. , Korolev, A. , Schwarzoenboeck, A. , Leroy, D. , & Um, J. (2017). Processing of ice cloud in situ data collected by bulk water, scattering, and imaging probes: fundamentals, uncertainties, and efforts toward consistency. Meteorological Monographs, 58, 11–11. 10.1175/amsmonographs-d-16-0007.1

[jame21128-bib-0336] McFarquhar, G. M. , Zhang, G. , Poellot, M. R. , Kok, G. L. , Mccoy, R. , Tooman, T. , Fridlind, A. , & Heymsfield, A. J. (2007). Ice properties of single‐layer stratocumulus during the mixed‐phase Arctic cloud experiment: 1. Observations. Journal of Geophysical Research, 112, D24201 10.1029/2007JD008633

[jame21128-bib-0337] McGraw, R. (2007). Numerica advection of correlated tracers: Preserving particle size/composition moment sequences during transport of aerosol mixtures. Journal of Physics Conference Series, 78, 012045 10.1088/1742-6596/78/1/012045

[jame21128-bib-0338] McTaggart‐Cowan, J. D. , & List, R. (1975). Collision and breakup of water drops at terminal velocity. Journal of the Atmospheric Sciences, 32, 1401–1411. 10.1175/1520-0469(1975)032<1401:cabowd>2.0.co;2

[jame21128-bib-0339] McTaggart‐Cowan, R. , Vaillancourt, P. A. , Zadra, A. , Chamberland, S. , Charron, M. , Corvec, S. , Milbrandt, J. A. , Paquin‐Ricard, D. , Patoine, A. , Roch, M. , Separovic, L. , & Yang, J. (2019). Modernization of atmospheric physics parameterization in Canadian NWP. Journal of Advances in Modeling Earth Systems, 11, 3593–3653. 10.1029/2019MS001781

[jame21128-bib-0340] Mellado, J. P. (2010). The evaporatively driven cloud‐top mixing layer. Journal of Fluid Mechanics, 660, 5–36. 10.1017/S0022112010002831

[jame21128-bib-0341] Meyers, M. P. , DeMott, P. J. , & Cotton, W. R. (1992). New primary ice‐nucleation parameterizations in an explicit cloud model. Journal of Applied Meteorology, 31, 708–721. 10.1175/1520-0450(1992)031<0708:npinpi>2.0.co;2

[jame21128-bib-0342] Meyers, M. P. , Walko, R. L. , Harrington, J. Y. , & Cotton, W. R. (1997). New RAMS cloud microphysics parameterization. Part II: The two‐moment scheme. Atmospheric Research, 45, 3–39. 10.1016/s0169-8095(97)00018-5

[jame21128-bib-0343] Milbrandt, J. A. , Belair, S. , Faucher, M. , Vallee, M. , Carrera, M. L. , & Glazer, A. (2016). The pan‐Canadian high resolution (2.5 km) deterministic prediction system. Weather and Forecasting, 31, 1791–1816. 10.1175/WAF-D-16-0035.1

[jame21128-bib-0344] Milbrandt, J. A. , & Morrison, H. (2016). Parameterization of cloud microphysics based on the prediction of bulk ice particle properties. Part III: Introduction of multiple free categories. Journal of the Atmospheric Sciences, 73(3), 975–995. 10.1175/jas-d-15-0204.1

[jame21128-bib-0345] Milbrandt, J. A. , & Yau, M. K. (2005a). A multimoment bulk microphysics parameterization. Part I: Analysis of the role of the spectral shape parameter. Journal of the Atmospheric Sciences, 62(9), 3051–3064. 10.1175/jas3534.1

[jame21128-bib-0346] Milbrandt, J. A. , & Yau, M. K. (2005b). A multimoment bulk microphysics parameterization. Part II: A proposed three‐moment closure and scheme description. Journal of the Atmospheric Sciences, 62(9), 3065–3081. 10.1175/jas3535.1

[jame21128-bib-0347] Milbrandt, J. A. , Yau, M. K. , Mailhot, J. , Belair, S. , & McTaggart‐Cowan, R. (2010). Simulation of an orographic precipitation event during IMPROVE‐2. Part II: Sensitivity to the number of moments in the bulk microphysics scheme. Monthly Weather Review, 138, 625–642. 10.1175/2009MWR3121.1

[jame21128-bib-0348] Miller, D. J. , Zhang, Z. , Platnick, S. E. , Ackerman, A. S. , Werner, F. , Cornet, C. , & Knobelspiesse, K. (2018). Comparisons of bispectral and polarimetric cloud microphysical retrievals using LES‐Satellite retrieval simulator. Atmospheric Measurement Techniques, 11, 3689–3715. 10.5194/amt-11-3689-2018

[jame21128-bib-0349] Minamide, M. , & Zhang, F. (2018). Assimilation of all‐sky infrared radiances from Himawari‐8 and impacts of moisture and hydrometeor initialization on convection‐permitting tropical cyclone prediction. Monthly Weather Review, 146, 3241–3258. 10.1175/MWR-D-17-0367.1

[jame21128-bib-0350] Ming, Y. , Ramaswamy, V. , Donner, L. J. , Phillips, V. T. , Klein, S. A. , Ginous, P. A. , & Horowitz, L. W. (2007). Modeling the interactions between aerosols and liquid water clouds with a self‐consistent cloud scheme in a general circulation model. Journal of the Atmospheric Sciences, 64, 1189–1209. 10.1175/jas3874.1

[jame21128-bib-0351] Misumi, R. , Hashimoto, A. , Murakami, M. , Kuba, N. , Orikasa, N. , Saito, A. , Tajiri, T. , Yamashita, K. , & Chen, J. (2010). Microphysical structure of a developing convective snow cloud simulated by an improved version of the multi‐dimensional bin model. Atmospheric Science Letters, 11, 186–191. 10.1002/asl.268

[jame21128-bib-0352] Mittermaier, M. , Roberts, N. , & Thompson, S. (2013). A long‐term assessment of precipitation forecast skill using the fractions skill score. Meteorological Applications, 20, 176–186. 10.1002/met.296

[jame21128-bib-0353] Moisseev, D. N. , Lautaportti, S. , Tyynela, J. , & Lim, S. (2015). Dual‐polarization radar signatures in snowstorms: Role of snowflake aggregation. Journal of Geophysical Research, 120, 12,644–12,655. 10.1002/2015JD023884

[jame21128-bib-0354] Morris, C. N. (1986). Comment on “why isn't everyone a Bayesian?”. American Statistician, 40, 7–8. 10.2307/2683108

[jame21128-bib-0355] Morrison, H. , Curry, J. A. , & Khvorostyanov, V. I. (2005). A new double‐moment microphysics parameterization for application in cloud and climate models. Part I: Description. Journal of the Atmospheric Sciences, 62(6), 1665–1677. 10.1175/jas3446.1

[jame21128-bib-0356] Morrison, H. , & Gettelman, A. (2008). A new two‐moment bulk stratiform cloud microphysics scheme in the community atmosphere model, version 3 (CAM3). Part I: Description and numerical tests. Journal of Climate, 21, 3642–3659. 10.1175/2008jcli2105.1

[jame21128-bib-0357] Morrison, H. , & Grabowski, W. W. (2007). Comparison of bulk and bin warm rain microphysics models using a kinematic framework. Journal of the Atmospheric Sciences, 64, 2839–2861. 10.1175/jas3980

[jame21128-bib-0358] Morrison, H. , & Grabowski, W. W. (2008). A novel approach for representing ice microphysics in models: Description and tests using a kinematic framework. Journal of the Atmospheric Sciences, 65, 1528–1548. 10.1175/2007JAS2491.1

[jame21128-bib-0359] Morrison, H. , Jensen, A. A. , Harrington, J. Y. , & Milbrandt, J. A. (2016). Advection of coupled hydrometeor quantities in bulk cloud microphysics schemes. Monthly Weather Review, 144(8), 2809–2829. 10.1175/mwr-d-15-0368.1

[jame21128-bib-0360] Morrison, H. , Kumjian, M. R. , Martinkus, C. P. , Prat, O. P. , & van Lier‐Walqui, M. (2019). A general N‐moment normalization method for deriving rain drop size distribution scaling relationships. Journal of Applied Meteorology and Climatology, 68, 247–267. 10.1175/jamc-d-18-0060.1

[jame21128-bib-0361] Morrison, H. , & Milbrandt, J. A. (2011). Comparison of two‐moment bulk microphysics schemes in idealized supercell thunderstorm simulations. Monthly Weather Review, 139, 1103–1130. 10.1175/2010mwr3433.1

[jame21128-bib-0362] Morrison, H. , & Milbrandt, J. A. (2015). Parameterization of cloud microphysics based on the prediction of bulk ice particle properties. Part I: Scheme description and idealized tests. Journal of the Atmospheric Sciences, 72, 287–311. 10.1175/jas-d-14-0065.1

[jame21128-bib-0363] Morrison, H. , Milbrandt, J. A. , Bryan, G. H. , Ikeda, K. , Tessendorf, S. A. , & Thompson, G. (2015). Parameterization of cloud microphysics based on the prediction of bulk ice particle properties. Part II: Case study comparisons with observations and other schemes. Journal of the Atmospheric Sciences, 72, 312–339. 10.1175/jas-d-14-0066.1

[jame21128-bib-0364] Morrison, H. , Tessendorf, S. A. , Ikeda, K. , & Thompson, G. (2012). Sensitivity of a simulated midlatitude squall line to parameterization of raindrop breakup. Monthly Weather Review, 140(8), 2437–2460. 10.1175/mwr-d-11-00283.1

[jame21128-bib-0365] Morrison, H. , van Lier‐Walqui, M. , Kumjian, M. R. , & Prat, O. P. (2020). A Bayesian approach for statistical‐physical bulk parameterization of rain microphysics. Part I: Scheme description. Journal of the Atmospheric Sciences, 77, 1019–1041. 10.1175/JAS-D-19-0070.1

[jame21128-bib-0366] Morrison, H. , Witte, M. , Bryan, G. H. , Harrington, J. Y. , & Lebo, Z. J. (2018). Broadening of modeled cloud droplet spectra using bin microphysics in an Eulerian spatial domain. Journal of the Atmospheric Sciences, 75, 4005–4030. 10.1175/jas-d-18-0055.1

[jame21128-bib-0367] Mossop, S. C. (1970). Concentrations of ice crystals in clouds. Bulletin of the American Meteorological Society, 51(6), 474–479. 10.1175/1520-0477(1970)051<0474:COICIC>2.0.CO;2

[jame21128-bib-0369] Mossop, S. C. (1985). The origin and concentration of ice crystals in clouds. Bulletin of the American Meteorological Society, 66(3), 264–273. 10.1175/1520-0477(1985)066<0264:TOACOI>2.0.CO;2

[jame21128-bib-0370] Mossop, S. C. , Cottis, R. E. , & Bartlett, B. M. (1972). Ice crystal concentrations in cumulus and stratocumulus clouds. Quarterly Journal of the Royal Meteorological Society, 98 10.1002/qj.49709841509

[jame21128-bib-0371] Mossop, S. C. , & Hallett, J. (1974). Ice crystal concentration in cumulus clouds: Influence of the drop spectrum. Science, 186, 632–634. 10.1126/science.186.4164.632 17833720

[jame21128-bib-0373] Murray, B. J. , Broadley, S. L. , Wilson, T. W. , Atkinson, J. D. , & Wills, R. H. (2011). Heterogeneous freezing of water droplets containing kaolinite particles. Atmospheric Chemistry and Physics, 11, 4191–4207. 10.5194/acp-11-4191-2011

[jame21128-bib-0374] National Research Council (2003). Critical issues in weather modification research. Washington, D.C.: The National Academies Press. 130 pp.

[jame21128-bib-0375] Naumann, A. K. , & Seifert, A. (2016). Evolution of the shape of the raindrop size distribution in simulated shallow cumulus. Journal of the Atmospheric Sciences, 73(6), 2279–2297. 10.1175/jas-d-15-0263.1

[jame21128-bib-0376] Nelson, J. (1994). A theoretical study of ice crystal growth in the atmosphere, Ph.D. thesis, 183 pp. Seattle, Washington: University of Washington.

[jame21128-bib-0377] Nelson, J. (2001). Growth mechanisms to explain the primary and secondary habits of snow crystals. Philosophical Magazine, 81A, 2337–2373. 10.1080/01418610108217152

[jame21128-bib-0378] Nelson, J. (2005). Interactive comment on “supersaturation dehydration, and denitrification in Arctic cirrus” by B. Kärcher. Atmospheric Chemistry and Physics Discussions, 5, S257–S260, https://www.atmos‐chem‐phys‐discuss.net/5/S545/2005/acpd‐5‐S545‐2005.pdf. 10.5194/acpd-5-1829-2005

[jame21128-bib-0379] Nelson, J. , & Swanson, B. (2019). Air pockets and secondary habits in ice from lateral‐type growth. Atmospheric Chemistry and Physics Discussions, 40 10.5194/acp-2019-280 accepted

[jame21128-bib-0380] Niedermeier, D. , Shaw, R. A. , Hartmann, S. , Wex, H. , Clauss, T. , Voigtländer, J. , & Stratmann, F. (2011). Heterogeneous ice nucleation: Exploring the transition from stochastic to singular freezing behavior. Atmospheric Chemistry and Physics, 11, 8767–8775. 10.5194/acp-11-8767-2011

[jame21128-bib-0381] Noh, Y. , Oh, D. , Hoffmann, F. , & Raasch, S. (2018). A cloud microphysics parameterization for shallow cumulus clouds based on Lagrangian cloud model simulations. Journal of the Atmospheric Sciences, 75, 4031–4047. 10.1175/JASD-18-0080.1

[jame21128-bib-0382] Nousiainen, T. , Lindqvist, H. , McFarquhar, G. M. , & Um, J. (2011). Small irregular ice crystals in tropical cirrus. Journal of the Atmospheric Sciences, 68, 2614–2627. 10.1175/2011JAS3733.1

[jame21128-bib-0383] O’Connor, E. J. , Hogan, R. J. , & Illingworth, A. J. (2005). Retrieving stratocumulus drizzle parameters using Doppler radar and lidar. Journal of Applied Meteorology, 44, 14–27. 10.1175/JAM-2181.1

[jame21128-bib-0384] O’Gorman, P. A. , & Dwyer, J. G. (2018). Using machine learning to parameterize moist convection: Potential for modeling of climate, climate change, and extreme events. Journal of Advances in Modeling Earth Systems, 10, 2548–2563. 10.1029/2018ms001351

[jame21128-bib-0385] O’Rourke, P. J. (1981). Collective drop effects on vaporizing liquid sprays, Ph.D. thesis, Princeton, New Jersey: Princeton University.

[jame21128-bib-0386] Ollinaho, P. , Laine, M. , Solonen, A. , Haario, H. , & Järvinen, H. (2013). NWP model forecast skill optimization via closure parameter variations. Quarterly Journal of the Royal Meteorological Society, 139, 1520–1532. 10.1002/qj.2044

[jame21128-bib-0387] Ono, A. (1971). Some aspects of the natural glaciation processes in relatively warm maritime clouds. Journal of the Meteorological Society of Japan, 49A(0), 845–858. 10.2151/jmsj1965.49A.0_845

[jame21128-bib-0388] Ono, A. (1972). Evidence on the nature of ice crystal multiplication processes in natural cloud. Journal de Recherches Atmospheriques, 6, 399–408.

[jame21128-bib-0389] Oraltay, R. G. , & Hallett, J. (1989). Evaporation and melting of ice crystals: A laboratory study. Atmospheric Research, 24(1–4), 169–189. 10.1016/0169-8095(89)90044-6

[jame21128-bib-0390] Ormel, C. W. , & Spaans, M. (2008). Monte Carlo simulation of particle interactions at high dynamic range: Advancing beyond the Googol. The Astrophysical Journal, 684, 1291–1309. 10.1086/590052

[jame21128-bib-0391] O'Sullivan, D. , Murray, B. J. , Malkin, T. L. , Whale, T. F. , Umo, N. S. , Atkinson, J. D. , Price, H. C. , Baustian, K. J. , Browse, J. , & Webb, M. E. (2014). Ice nucleation by fertile soil dusts: Relative importance of mineral and biogenic components. Atmospheric Chemistry and Physics, 14, 1853–1867. 10.5194/acp-14-1853-2014

[jame21128-bib-0392] Oue, M. , Galletti, M. , Verlinde, J. , Ryzhkov, A. , & Lu, Y. (2016). Use of X‐band differential reflectivity measurements to study shallow Arctic mixed‐phase clouds. Journal of Applied Meteorology and Climatology, 55, 403–424. 10.1175/JAMC-D-15-0168.1

[jame21128-bib-0393] Ovtchinnikov, M. , & Easter, R. C. (2009). Nonlinear advection algorithms applied to interrated tracers: Errors and implications for modeling cloud‐aerosol interactions. Monthly Weather Review, 137, 632–644. 10.1175/2008mwr2626.1

[jame21128-bib-0394] Paganini, M. , de Oliviera, L. , & Nachman, B. (2018). CaloGAN: Simulating 3D high energy particle showers in multilayer electromagnetic calorimeters with generative adversarial networks. Physical Review, 97, 04021 10.1103/PhysRevD.97.014021 29437460

[jame21128-bib-0395] Palmer, T. N. , Buizza, R. , Doblas‐Reyes, F. , Jung, T. , Leutbecher, M. , Shutts, G. , Steinheimer, M. , & Weisheimer, A. (2009). Stochastic parametrization and model uncertainty. ECMWF Technical Memorandum, 598, 42 pp. [Available at https://www2.physics.ox.ac.uk/sites/default/files/2011-08-15/techmemo598_stochphys_2009_pdf_50419.pdf.]. 10.21957/ps8gbwbdv

[jame21128-bib-0396] Palmer, T. N. , Doblas‐Reyes, F. J. , Weisheimer, A. , & Rodwell, M. J. (2008). Toward seamless prediction: Calbibration of climate change projections using seasonal forecasts. Bulletin of the American Meteorological Society, 89, 459–470. 10.1175/BAMS-89-4-459

[jame21128-bib-0397] Paoli, R. , Hélie, J. , & Poinsot, T. (2004). Contrail formation in aircraft wakes. Journal of Fluid Mechanics, 502, 361–373. 10.1017/S0022112003007808

[jame21128-bib-0398] Park, H. , Ryzhkov, A. V. , Zrnic, D. S. , & Kim, K.‐E. (2009). The hydrometeor classification algorithm for the polarimetric WSR‐88D: Description and application to an MCS. Weather and Forecasting, 24, 730–748. 10.1175/2008waf2222205.1

[jame21128-bib-0399] Paukert, M. , Fan, J. , Rasch, P. J. , Morrison, H. , Milbrandt, J. A. , Shpund, K. , & Khain, A. P. (2018). Three‐moment representation of rain in a bulk microphysics model. Journal of Advances in Modeling Earth Systems, 11, 257–277. 10.1029/2018ms001512

[jame21128-bib-0400] Paukert, M. , Hoose, C. , & Simmel, M. (2017). Redistribution of ice nuclei between cloud and rain droplets: Parameterization and application to deep convective clouds. Journal of Advances in Modeling Earth Systems, 9, 514–535. 10.1002/2016MS000841

[jame21128-bib-0401] Pena, J. A. , de Pena, R. G. , & Hosler, C. L. (1969). Freezing of water droplets in equilibrium with different gases. Journal of the Atmospheric Sciences, 26(2), 309–314. 10.1175/1520-0469(1969)026<0309:FOWDIE>2.0.CO;2

[jame21128-bib-0402] Phillips, T. J. P. , DeMott, P. J. , & Andronache, C. (2008). An empirical parameterization of heterogeneous ice nucleation for multiple chemical species. Journal of the Atmospheric Sciences, 65, 2757–2783. 10.1175/2007jas2546.1

[jame21128-bib-0403] Phillips, V. T. J. , Patade, S. , Gutierrez, J. , & Bansemer, A. (2018). Secondary ice production by fragmentation of freezing drops: Formulation and theory. Journal of the Atmospheric Sciences, 75, 3031–3070. 10.1175/jas-d-17-0190.1

[jame21128-bib-0404] Pincus, R. , & Klein, S. A. (2000). Unresolved spatial variability and microphysical process rates in large‐scale models. Journal of Geophysical Research, 105, 27,059–27,065. 10.1029/2000JD900504

[jame21128-bib-0405] Pinsky, M. , Mazin, I. P. , Korolev, A. , & Khain, A. (2014). Supersaturation and diffusional droplet growth in liquid clouds: Polydisperse spectra. Journal of Geophysical Research, 119, 12,872–12,887. 10.1029/2014JD021885

[jame21128-bib-0408] Planche, C. , Tridon, F. , Banson, S. , Thompson, G. , Monier, M. , Battaglia, A. , & Wobrock, W. (2019). On the realism of the rain microphysics representation of a squall line in the WRF model. Part II: Sensitivity studies on the rain drop size distributions. Monthly Weather Review, 147, 2811–2825. 10.1175/mwr-d-18-0019.1

[jame21128-bib-0409] Platnick, S. E. (2000). Vertical photon transport in cloud remote sensing problems. Journal of Geophysical Research, 105(D18), 22,919–22,935. 10.1029/2000JD900333

[jame21128-bib-0410] Pokrifka, G. (2018). Using laboratory measurements of vapor‐grown ice crystals to infer surface kinetics and estimate the deposition coefficient, M.S. thesis (89 pp). University Park, Pennsylvania: Dept. of Meteorology and Atmospheric Science, The Pennsylvania State University.

[jame21128-bib-0411] Popper, K. R. (1959). The logic of scientific discovery. London, U.K. (re‐printed in 1999 by Routledge: Hutchinson Education. 480 pp.

[jame21128-bib-0412] Popper, K. R. (1978). The two fundamental problems of the theory of knowledge, Tubingen (re‐printed in English in 2009 by Routledge) (509 pp). Abingdon, U.K.: Routledge.

[jame21128-bib-0413] Posselt, D. J. (2016). A Bayesian examination of deep convective squall line sensitivity to changes in cloud microphysical parameters. Journal of the Atmospheric Sciences, 73, 637–665. 10.1175/jas-d-15-0159.1

[jame21128-bib-0414] Posselt, D. J. , Fryxell, B. , Molod, A. , & Williams, B. (2016). Quantitative sensitivity analysis of physical parameterizations for cases of deep convection in the NASA GEOS‐5 model. Journal of Climate, 29, 455–479. 10.1175/jcli-d-15-0250.1

[jame21128-bib-0415] Posselt, D. J. , He, F. , Bukowski, J. , & Reid, J. S. (2019). On the relative sensitivity of a tropical deep convective storm to changes in environment and cloud microphysical parameters. Journal of the Atmospheric Sciences, 76, 1163–1185. 10.1175/JAS-D-18-0181.1

[jame21128-bib-0416] Posselt, D. J. , Kessler, J. , & Mace, G. G. (2017). Bayesian retrievals of vertically resolved cloud particle size distribution properties. Journal of Applied Meteorology and Climatology, 56, 745–765. 10.1175/JAMC-D-16-0276.1

[jame21128-bib-0417] Posselt, D. J. , Li, X. , Tushaus, S. A. , & Mecikalski, J. R. (2015). Assimilation of dual‐polarization radar observations in mixed‐ and ice‐phase regions of convective storms: Information content and forward model errors. Monthly Weather Review, 143, 2611–2636. 10.1175/mwr-d-14-00347.1

[jame21128-bib-1006] Posselt, D. J. , & Mace, G. G. (2014). MCMC‐based assessment of the error characteristics of a surface‐based combined radar-passive microwave cloud property retrieval. Journal of Applied Meteorology and Climatology, 53(8), 2034–2057. 10.1175/JAMC-D-13-0237.1

[jame21128-bib-0418] Posselt, D. J. , & Vukicevic, T. (2010). Robust characterization of model physics uncertainty for simulations of deep moist convection. Monthly Weather Review, 138, 1513–1535. 10.1175/2009mwr3094.1

[jame21128-bib-0419] Posselt, R. , & Lohmann, U. (2009). Sensitivity of the total anthropogenic aerosol effect to the treatment of rain in a global climate model. Geophysical Research Letters, 36, L02805 10.1029/2008GL035796

[jame21128-bib-0420] Prat, O. P. , & Barros, A. P. (2007). A robust numerical solution of the stochastic collection‐breakup equation for warm rain. Journal of Applied Meteorology and Climatology, 46, 1480–1497. 10.1175/jam2544.1

[jame21128-bib-0421] Prat, O. P. , & Barros, A. P. (2009). Exploring the transient behavior of Z‐R relationships: Implications for radar rainfall estimation. Journal of Applied Meteorology and Climatology, 48, 2127–2143. 10.1175/2009JAMC2165.1

[jame21128-bib-0422] Prat, O. P. , Barros, A. P. , & Testik, F. Y. (2012). On the influence of raindrop collision outcomes on equilibrium drop size distributions. Journal of the Atmospheric Sciences, 69, 1534–1546. 10.1175/JAS-D-11-0192.1

[jame21128-bib-0423] Prein, A. F. , Langhans, W. , Fosser, G. , Ferrone, A. , Ban, N. , Goergen, K. , Keller, M. , Tölle, M. , Gutjahr, O. , Feser, F. , Brisson, E. , Kollet, S. , Schmidli, J. , Lipzig, N. P. M. , & Leung, R. (2015). A review on regional convection‐permitting climate modeling: Demonstrations, prospects, and challenges. Reviews of Geophysics, 53, 323–361. 10.1002/2014RG000475 27478878 PMC4949718

[jame21128-bib-0424] Pruppacher, H. R. , & Klett, J. D. (1997). Microphysics of clouds and precipitation (2nd ed.). Dordrecht, The Netherlands: Kluwer Academic Publishers. 954 pp.

[jame21128-bib-0425] Pruppacher, H. R. , & Rasmussen, R. (1979). A wind tunnel investigation of the rate of evaporation of large water drops falling at terminal velocity in air. Journal of the Atmospheric Sciences, 36, 1255–1260. 10.1175/1520-0469(1979)036<1255:awtiot>2.0.co;2

[jame21128-bib-0426] Puzanov, V. P. , & Accuratov, V. I. (1952). Towards formation mechanism of some types of hail. Gidrologia i Meteorologia, N6, 29–33.

[jame21128-bib-0427] Qian, Y. , Wan, H. , Yang, B. , Golaz, J.‐. C. , Harrop, B. , Hou, Z. , Larson, V. E. , Leung, L. R. , Lin, G. , Lin, W. , Ma, P.‐. L. , Ma, H.‐. Y. , Rasch, P. , Singh, B. , Wang, H. , Xie, S. , & Zhang, K. (2018). Parametric sensitivity and uncertainty quantification in the version 1 of E3SM atmosphere model based on short perturbed parameter ensemble simulations. Journal of Geophysical Research, 123, 13,046–13,073. 10.1029/2018JD028927

[jame21128-bib-0428] Qu, Z. , Barker, H. W. , Korolev, A. V. , Milbrandt, J. A. , Heckman, I. , Bélair, S. , Leroyer, S. , Vaillancourt, P. A. , Wolde, M. , Schwarzenböck, A. , Leroy, D. , Strapp, J. W. , Cole, J. N. S. , Nguyen, L. , & Heidinger, A. (2018). Evaluation of a high‐resolution numerical weather prediction model's simulated clouds using observations from CloudSat, GOES‐13 and in situ aircraft. Quarterly Journal of the Royal Meteorological Society, 144, 1681–1694. 10.1002/qj.3318

[jame21128-bib-0429] Randall, D. , Khairoutdinov, M. , Arakawa, A. , & Grabowski, W. (2003). Breaking the cloud parameterization deadlock. Bulletin of the American Meteorological Society, 84, 1547–1564. 10.1175/bams-84-11-1547

[jame21128-bib-0430] Randall, D. A. , Bitz, C. M. , Danabasoglu, G. , Denning, A. S. , Gent, P. R. , Gettelman, A. , Griffies, S. M. , Lynch, P. , Morrison, H. , Pincus, R. , & Thuburn, J. (2019). 100 years of Earth system model development. Meteorological Monographs, 59, 12.1–12.66. 10.1175/amsmonographs-d-18-0018.1

[jame21128-bib-0431] Rangno, A. L. (2008). Fragmentation of freezing drops in shallow maritime frontal clouds. Journal of the Atmospheric Sciences, 65, 1455–1466. 10.1175/2007jas2295.1

[jame21128-bib-0432] Rangno, A. L. , & Hobbs, P. V. (2001). Ice particles in stratiform clouds in the Arctic and possible mechanisms for the production of high ice concentrations. Journal of Geophysical Research, 106, 15,065–15,075. 10.1029/2000JD900286

[jame21128-bib-0433] Raoult, N. M. , Jupp, T. E. , Cox, P. M. , & Luke, C. M. (2016). Land‐surface parameter optimisation using data assimilation techniques: The adJULES system V1.0. Geoscientific Model Development, 9, 2833–2852. 10.5194/gmd-9-2833-2016

[jame21128-bib-0434] Rasmussen, K. L. , Prein, A. F. , Rasmussen, R. M. , Ikeda, K. , & Liu, C. (2017). Changes in the convective population and thermodynamic environments in convection‐permitting regional climate simulations over the United States. Climate Dynamics, 1–26. 10.1007/s00382-017-4000-7

[jame21128-bib-0435] Rasmussen, R. , Liu, C. , Ikeda, K. , Gochis, D. , Yates, D. , Chen, F. , Tewari, M. , Barlage, M. , Dudhia, J. , Yu, W. , Miller, K. , Arsenault, K. , Grubišić, V. , Thompson, G. , & Gutmann, E. (2011). High‐resolution coupled climate runoff simulations of seasonal snowfall over Colorado: A process study of current and warmer climate. Journal of Climate, 24(12), 3015–3048. 10.1175/2010jcli3985.1

[jame21128-bib-0436] Rasmussen, R. M. , & Heymsfield, A. J. (1987). Melting and shedding of graupel and hail. Part I: Model physics. Journal of the Atmospheric Sciences, 44, 2754–2763. 10.1175/1520-0469(1987)044<2754:masoga>2.0.co;2

[jame21128-bib-0437] Rasmussen, R. M. , Levizzani, V. , & Pruppacher, H. R. (1984). A wind tunnel and theoretical study of the melting behavior of atmospheric ice particles. II: A theoretical study for frozen drops of radius < 500 um. Journal of the Atmospheric Sciences, 41, 374–380. 10.1175/1520-0469(1984)041<0374:awtats>2.0.co;2

[jame21128-bib-0438] Rasmussen, R. M. , & Pruppacher, H. R. (1982). A wind tunnel and theoretical study of the melting behavior of atmospheric ice particles. I: A wind tunnel study of frozen drops of radius < 500 um. Journal of the Atmospheric Sciences, 39, 152–158. 10.1175/1520-0469(1982)039<0152:awtats>2.0.co;2

[jame21128-bib-0439] Rasp, S. , Pritchard, M. S. , & Gentine, P. (2018). Deep learning to represent subgrid processes in climate models. Proceedings of the National Academy of Sciences, 115, 9684–9689. 10.1073/pnas.1810286115 PMC6166853 30190437

[jame21128-bib-0440] Reisin, T. , Levin, Z. , & Tzivion, S. (1996). Rain production in convective clouds as simulated in an axisymmetric model with detailed microphysics. Part I: Description of the model. Journal of the Atmospheric Sciences, 53, 497–519. 10.1175/1520-0469(1996)053<0497:rpicca>2.0.co;2

[jame21128-bib-0441] Reisner, J. , Rasmussen, R. M. , & Bruintjes, R. T. (1998). Explicit forecasting of supercooled liquid water in winter storms using the MM5 mesoscale model. Quarterly Journal of the Royal Meteorological Society, 124, 1071–1107. 10.1002/qj.49712454804

[jame21128-bib-0442] Rémillard, J. , Fridlind, A. M. , Ackerman, A. S. , Tselioudis, G. , Kollias, P. , Mechem, D. B. , Chandler, H. E. , Luke, E. , Wood, R. , Witte, M. K. , & Ayers, J. K. (2017). Use of cloud radar Doppler spectra to evaluate stratocumulus drizzle size distributions in large‐eddy simulations with size‐resolved microphysics. Journal of Applied Meteorology and Climatology, 56(12), 3263–3283. 10.1175/JAMC-D-17-0100.1 30740040 PMC6364314

[jame21128-bib-0443] Riechelmann, T. , Noh, Y. , & Raasch, S. (2012). A new method for large‐eddy simulations of clouds with Lagrangian droplets including the effects of turbulent collision. New Journal of Physics, 14 10.1088/1367-2630/14/6/065008

[jame21128-bib-0444] Riedi, J. C. , Marchant, B. , Platnick, S. E. , Baum, B. A. , Thieuleux, F. , Oudard, C. , Parol, F. , Nicolas, J. , & Dubuisson, P. (2010). Cloud thermodynamic phase inferred from merged POLDER and MODIS data. Atmospheric Chemistry and Physics, 10, 11,851–11,865. 10.5194/acp-10-11851-2010

[jame21128-bib-0445] Rodwell, M. J. , & Palmer, T. N. (2007). Using numerical weather prediction to assess climate models. Quarterly Journal of the Royal Meteorological Society, 133, 129–146. 10.1002/qj.23

[jame21128-bib-0446] Rosinski, J. , Langer, G. , & Nagamoto, C. T. (1972). On the effect of microdroplets from the surface of a freezing water drop. Journal of Applied Meteorology, 11, 405–406.

[jame21128-bib-0447] Rutledge, S. A. , & Hobbs, P. V. (1984). The mesoscale and microscale structure and organization of clouds and precipitation in midlatitude cyclones. XII: A diagnostic modeling study of precipitation development in narrow cold‐frontal rainbands. Journal of the Atmospheric Sciences, 41, 2949–2972. 10.1175/1520-0469(1984)041<2949:TMAMSA>2.0.CO;2

[jame21128-bib-0448] Ryzhkov, A. V. , Diederich, M. , Zhang, P. , & Simmer, C. (2014). Potential utilization of specific attenuation for rainfall estimation, mitigation of partial beam blockage, and radar networking. Journal of Atmospheric and Oceanic Technology, 31, 599–619. 10.1175/JTECH-D-13-00038.1

[jame21128-bib-0449] Ryzhkov, A. V. , Giangrande, S. E. , & Schuur, T. J. (2005). Rainfall estimation with a polarimetric prototype of the WSR‐88D. Journal of Applied Meteorology, 44, 502–515. 10.1175/JAM2213.1

[jame21128-bib-0450] Ryzhkov, A. V. , Zhang, P. , Reeves, H. , Kumjian, M. , Tschallener, T. , Troemel, S. , & Simmer, C. (2016). Quasi‐vertical profiles—A new way to look at polarimetric radar data. Journal of Atmospheric and Oceanic Technology, 33, 551–562. 10.1175/JTECH-D-15-0020.1

[jame21128-bib-0451] Ryzhkov, A. V. , & Zrnic, D. S. (1996). Assessment of rainfall measurement that uses specific differential phase. Journal of Applied Meteorology, 35, 2080–2090. 10.1175/1520-0450(1996)035<2080:AORMTU>2.0.CO;2

[jame21128-bib-0452] Ryzhkov, A. V. , & Zrnic, D. S. (2019). Radar polarimetry for weather observations. Springer. 487 pp.

[jame21128-bib-0453] Ryzhkov, A. V. , Zrnic, D. S. , & Gordon, B. A. (1998). Polarimetric method for ice water content determination. Journal of Applied Meteorology, 37, 125–134. 10.1175/1520-0450(1998)037<0125:PMFIWC>2.0.CO;2

[jame21128-bib-0454] Saito, M. , Yang, P. , Hu, Y. , Liu, X. , Loeb, N. G. , Smith, W. L. , & Minnis, P. (2019). An efficient method for microphysical property retrievals in vertically inhomogeneous marine water clouds using MODIS‐CloudSat measurements. Journal of Geophysical Research, 124, 2174–2193. 10.1029/2018JD029659

[jame21128-bib-0455] Saleeby, S. M. , & Cotton, W. R. (2008). A binned approach to cloud‐droplet riming implemented in a bulk microphysics model. Journal of Applied Meteorology and Climatology, 47, 694–703. 10.1175/2007jamc1664.1

[jame21128-bib-0456] Sambridge, M. , Bodin, T. , Gallgher, K. , & Tkalcic, H. (2013). Transdiomensional inference in the geosciences. Philosophical Transactions of the Royal Society A, 371, 20110547 10.1098/rtsa.2011.0547 23277604

[jame21128-bib-0457] Sanderson, B. M. , Piani, C. , Ingram, W. J. , Stone, D. A. , & Allen, M. R. (2008). Towards constraining climate sensitivity by linear analysis of feedback patterns in thousands of perturbed physics GCM simulations. Climate Dynamics, 30, 175–190. 10.1007/s00382-007-0280-7

[jame21128-bib-0458] Satoh, M. , Stevens, B. , Judt, F. , Khairoutdinov, M. , Lin, S.‐J. , Putman, W. M. , & Duben, P. (2019). Global cloud‐resolving models. Current Climate Change Reports, 5, 172–184. 10.1007/s40641-019-00131-0

[jame21128-bib-0459] Saunders, C. P. R. , & Wahab, N. M. A. (1975). The influence of electric fields on the aggregation of ice crystals. Journal of the Meteorological Society of Japan, 53, 121–126. 10.2151/jmsj1965.53.2_121

[jame21128-bib-0460] Schaefer, V. J. (1949). The formation of ice crystals in the laboratory and the atmosphere. Chemical Reviews, 44, 291–320. 10.1021/cr60138a004 18128332

[jame21128-bib-0461] Schlottke, J. W. , Straub, W. , Beheng, K. D. , Gomaa, H. , & Weigand, B. (2010). Numerical investigation of collision induced breakup of raindrops. Part I: Methodology and dependencies on collision and eccentricity. Journal of the Atmospheric Sciences, 67, 557–575. 10.1175/2009jas3174.1

[jame21128-bib-0462] Schmidt, D. P. , & Rutland, C. J. (2000). A new droplet collision algorithm. Journal of Computational Physics, 164, 62–80. 10.1006/jcph.2000.6568

[jame21128-bib-0463] Schmidt, G. A. , Bader, D. , Donner, L. J. , Elsaesser, G. S. , Golaz, J.‐C. , Hannay, C. , Molod, A. , Neale, R. B. , & Saha, S. (2017). Practice and philosophy of climate model tuning across six US modeling centers. Geoscientific Model Development, 10, 3207–3223. 10.5194/gmd-10-3207-2017 30595813 PMC6309528

[jame21128-bib-0464] Schmit, T. J. , Gunshor, M. M. , Menzel, W. P. , Li, J. , Bachmeier, S. , & Gurka, J. J. (2005). Introducing the next‐generation Advanced Baseline Imager (ABI) on GOES‐R. Bulletin of the American Meteorological Society, 86, 1079–1096. 10.1175/bams-86-8-1079

[jame21128-bib-0465] Schneider, T. , Lan, S. , Stuart, A. , & Teixeira, J. (2017). Earth system modeling 2.0: A blueprint for models that learn from observations and targeted high‐resolution simulations. Geophysical Research Letters, 44, 12,396–12,417. 10.1002/2017GL076101

[jame21128-bib-0466] Schönhuber, M. , Lammer, G. , & Randeu, W. L. (2008). The 2D‐video‐distrometer In Precipitation: Advances in measurement, estimation and prediction (pp. 3–31). Berlin, Heidelberg: Springer.

[jame21128-bib-0467] Schrom, R. S. , & Kumjian, M. R. (2018). Bulk‐density representations of branched planar ice crystals: Errors in the polarimetric radar variables. Journal of Applied Meteorology and Climatology, 57, 333–346. 10.1175/jamc-d-17-0114.1

[jame21128-bib-0468] Schrom, R. S. , & Kumjian, M. R. (2019). A probabilistic radar forward model for branched planar ice crystals. Journal of Applied Meteorology and Climatology, 58, 1245–1265. 10.1175/jamc-d-18-0204.1

[jame21128-bib-0469] Schrom, R. S. , Kumjian, M. R. , & Lu, Y. (2015). Polarimetric radar signatures of dendritic growth zones within Colorado winter storms. Journal of Applied Meteorology and Climatology, 54, 2365–2388. 10.1175/jamc-d-15-0004.1

[jame21128-bib-0470] Schwarzenboeck, A. , Shcherbakov, V. , Lefevre, R. , Gayet, J. F. , Pointin, Y. , & Duroure, C. (2009). Indications for stellar‐crystal fragmentation in Arctic clouds. Atmospheric Research, 92, 220–228. 10.1016/j.atmosres.2008.10.002

[jame21128-bib-0471] Scott, W. T. (1968). Analytic studies of cloud droplet coalescence. Journal of the Atmospheric Sciences, 25, 54–65. 10.1175/1520-0469(1968)025<0054:asocdc>2.0.co;2

[jame21128-bib-0472] Seifert, A. (2008). On the parameterization of evaporation of raindrops as simulated by a one‐dimensional rainshaft model. Journal of the Atmospheric Sciences, 65(11), 3608–3619. 10.1175/2008jas2586.1

[jame21128-bib-0473] Seifert, A. , & Beheng, K. (2006). A two‐moment cloud microphysics parameterization for mixed‐phase clouds. Part 1: Model description. Meteorology and Atmospheric Physics, 92, 45–66. 10.1007/s00703-005-0112-4

[jame21128-bib-0474] Seifert, A. , & Beheng, K. D. (2001). A double‐moment parameterization for simulating autoconversion, accretion and self‐collection. Atmospheric Research, 59–60, 265–281. 10.1016/s0169-8095(01)00126-0

[jame21128-bib-0475] Seifert, A. , Khain, A. , Blahak, U. , & Beheng, K. D. (2005). Possible effects of collisional breakup on mixed‐phase deep convection simulated by a spectral (bin) cloud model. Journal of the Atmospheric Sciences, 62, 1917–1931. 10.1175/jas3432.1

[jame21128-bib-0476] Seifert, A. , Leinonen, J. , Siewert, C. , & Kneifel, S. (2019). The geometry of rimed aggregate snowflakes: A modeling Study. Journal of Advances in Modeling Earth Systems, 11, 712–731. 10.1029/2018MS001519

[jame21128-bib-0477] Seity, Y. , Brousseau, P. , Malardel, S. , Hello, G. , Bénard, P. , Bouttier, F. , Lac, C. , & Masson, V. (2010). The AROME‐France convective‐scale operational model. Monthly Weather Review, 139, 876–913. 10.1175/2010MWR3425.1

[jame21128-bib-0478] Shaw, R. A. (2000). Supersaturation intermittency in turbulent clouds. Journal of the Atmospheric Sciences, 57, 3452–3456. 10.1175/1520-0469(2000)057<3452:siitc>2.0.co;2

[jame21128-bib-0479] Shi, Y. , Davis, K. J. , Zhang, F. , Duffy, C. J. , & Yu, X. (2015). Parameter estimation of a physically‐based land surface hydrologic model using an ensemble Kalman filter: A multivariate real‐data experiment. Advances in Water Resources, 83, 421–427. 10.1016/j.advwatres.2015.06.009

[jame21128-bib-0480] Shima, S. , Kusano, K. , Kawano, A. , Sugiyama, T. , & Kawahara, S. (2009). The super‐droplet method for the numerical simulation of clouds and precipitation: A particle‐based and probabilistic microphysics model coupled with a non‐hydrostatic model. Quarterly Journal of the Royal Meteorological Society, 135, 1307–1320. 10.1002/qj.441

[jame21128-bib-0481] Shima, S. , Sato, Y. , Hashimoto, A. , & Misumi, R. (2019). Predicting the morphology of ice particles in deep convection using the super‐droplet method: Development and evaluation of SCALE‐SDM 0.2.5–2.2.0/2.2.1. Geoscientific Model Development Discussion. 10.5194/gmd-2019-294

[jame21128-bib-0482] Shipway, B. J. , & Hill, A. A. (2012). Diagnosis of systematic differences between multiple parametrizations of warm rain microphysics using a kinematic framework. Quarterly Journal of the Royal Meteorological Society, 138(669), 2196–2211. 10.1002/qj.1913

[jame21128-bib-0483] Shirgaonkar, A. , & Lele, S. (2006). Large eddy simulation of early stage contrails: Effect of atmospheric properties, 44th AIAA Aerospace Sciences Meeting and Exhibit*,* 1–13. 10.2514/6.2006-1414

[jame21128-bib-0484] Shuman, F. G. , & Hovermale, J. (1968). An operational six‐layer primitive equation model. Journal of Applied Meteorology, 7, 525–547. 10.1175/1520-0450(1968)007<0525:aoslpe>2.0.co;2

[jame21128-bib-0485] Skamarock, W. C. , J. B. Klemp , J. Dudhia , D. O. Gill , D. M. Barker , W. Wang , & J. G. Powers (2008) A description of the advanced research WRF version 3, NCAR tech. note NCAR/TN‐475+STR (113 pp). Boulder, Colorado: National Center for Atmospheric Research.

[jame21128-bib-0486] Slater, B. , Michaelides, A. , Salzmann, C. G. , & Lohmann, U. (2016). A blue‐sky approach to understanding cloud formation. Bulletin of the American Meteorological Society, 97(10), 1797–1802. 10.1175/BAMS-D-15-00131.1

[jame21128-bib-0487] Smith, T. J. , & Marshall, L. A. (2008). Bayesian methods in hydrologic modeling: A study of recent advancements in Markov chain Monte Carlo techniques. Water Resources Research, 44, W00B05 10.1029/2007WR006705

[jame21128-bib-0488] Smoluchowski, M. V. (1916). Drei vortrage uber diffusion, Brownsche bewegung und koagulation von kolloidteilchen. Physikalishce Zeitschrift, 17, 557–585.

[jame21128-bib-0489] Sölch, I. , & Kärcher, B. (2010). A large‐eddy model for cirrus clouds with explicit aerosol and ice microphysics and Lagrangian ice particle tracking. Quarterly Journal of the Royal Meteorological Society, 136, 2074–2093. 10.1002/qj.689

[jame21128-bib-0490] Sosso, G. C. , Whale, T. F. , Holden, M. A. , Pedevilla, P. , Murray, B. J. , & Michaelides, A. (2018). Unravelling the origins of ice nucleation on organic crystals. Chemical Science, 2041–6520, 2041–6539. 10.1039/C8SC02753F PMC6238755 30542556

[jame21128-bib-0491] Sraj, I. , Zedler, S. E. , Knio, O. M. , Jackson, C. S. , & Hoteit, I. (2016). Polynomial chaos‐based Bayesian inference of k‐profile parameterization in a general circulation model of the tropical Pacific. Monthly Weather Review, 144, 4621–4640. 10.1175/MWR-D-15-0394.1

[jame21128-bib-0492] Srivastava, R. C. (1967). On the role of coalescence between raindrops in shaping their size distribution. Journal of the Atmospheric Sciences, 24, 287–292. 10.1175/1520-0469(1967)024<0287:OTROCB>2.0.CO;2

[jame21128-bib-0493] Stanford, M. W. , Morrison, H. , Varble, A. , Berner, J. , Wu, W. , McFarquhar, G. , & Milbrandt, J. A. (2019). Sensitivity of simulated deep convection to a stochastic ice microphysics framework. Journal of Advances in Modeling Earth Systems, 11, 3362–3389. 10.1029/2019MS001730

[jame21128-bib-0494] Stein, T. H. M. , Hogan, R. J. , Clark, P. A. , Halliwell, C. E. , Hanley, K. E. , Lean, H. W. , Nicol, J. C. , & Plant, R. S. (2015). The DYMECS project: A statistical approach for the evaluation of convective storms in high‐resolution NWP models. Bulletin of the American Meteorological Society, 96, 939–951. 10.1175/BAMS-D-13-00279.1

[jame21128-bib-0495] Steiner, M. , Houze, R. A. Jr. , & Yuter, S. E. (1995). Climatological characterization of three‐dimensional storm structure from operational radar and rain gauge data. Journal of Applied Meteorology, 34, 1978–2007. 10.1175/1520-0450(1995)034<1978:ccotds>2.0.co;2

[jame21128-bib-0496] Steinke, I. , Funk, R. , Busse, J. , Iturri, A. , Kirchen, S. , Leue, M. , Möhler, O. , Schwartz, T. , Schnaiter, M. , Sierau, B. , Toprak, E. , Ullrich, R. , Ulrich, A. , Hoose, C. , & Leisner, T. (2016). Ice nucleation activity of agricultural soil dust aerosols from Mongolia, Argentina, and Germany. Journal of Geophysical Research: Atmospheres, 121, 13,559–13,576. 10.1002/2016JD025160

[jame21128-bib-0497] Stephens, G. L. , van den Heever, S. C. , Haddad, Z. S. , Posselt, D. J. , Storer, R. L. , Grant, L. D. , Sy, O. O. , Rao, T. N. , Tanelli, S. , & Peral, E. (2019). A distributed small satellite approach for measuring convective transports in the Earth's atmosphere. IEEE Transactions on Geoscience and Remote Sensing, 58 10.1109/TGRS.2019.2918090

[jame21128-bib-0498] Stevens, B. , Feingold, G. , Cotton, W. R. , & Walko, R. L. (1996). Elements of the microphysical structure of numerically simulated of non‐precipitating stratocumulus. Journal of the Atmospheric Sciences, 53, 980–1006. 10.1175/1520-0469(1996)053<0980:eotmso>2.0.co;2

[jame21128-bib-0499] Stevens, B. , Satoh, M. , Auger, L. , Biercamp, J. , Bretherton, C. S. , Chen, X. , Düben, P. , Judt, F. , Khairoutdinov, M. , Klocke, D. , Kodama, C. , Kornblueh, L. , Lin, S.‐J. , Neumann, P. , Putman, W. M. , Röber, N. , Shibuya, R. , Vanniere, B. , Vidale, P. L. , Wedi, N. , & Zhou, L. (2019). DYAMOND: The dynamics of the atmospheric general circulation modeled on non‐hydrostatic domains. Progress in Earth and Planetary Science, 6, 61 10.1186/s40645-019-0304-z

[jame21128-bib-0500] Stevens, B. , & Seifert, A. (2008). Understanding macrophysical outcomes of microphysical choices in simulations of shallow cumulus convection. Journal of the Meteorological Society of Japan, 86A, 143–162. 10.2151/jmsj.86a.143

[jame21128-bib-0501] Stith, J. L. , Avallone, L. M. , Bansemer, A. , Basarab, B. , Dorsi, S. W. , Fuchs, B. , Lawson, R. P. , Rogers, D. C. , Rutledge, S. , & Toohey, D. W. (2014). Ice particles in the upper anvil regions of midlatitude continental thunderstorms: the case for frozen‐drop aggregates. Atmospheric Chemistry and Physics, 14, 1973–1985. 10.5194/acp-14-1973-2014

[jame21128-bib-0502] Stoelinga, M. T. , Locatelli, J. D. , & Woods, C. P. (2007). The occurrence of “irregular” ice particles in stratiform clouds. Journal of the Atmospheric Sciences, 64, 2740–2750. 10.1175/jas3962.1

[jame21128-bib-0503] Storelvmo, R. , Hoose, C. , & Erikkson, P. (2011). Global modeling of mixed‐phase clouds—The albedo and lifetime effects of aerosols. Journal of Geophysical Research, 116, D05207 10.1029/2010JD014724

[jame21128-bib-0505] Straka, J. M. , Zrnic, D. S. , & Ryzhkov, A. V. (2000). Bulk hydrometeor classification and quantification using polarimetric radar data: Synthesis of relations. Journal of Applied Meteorology, 39, 1341–1372. 10.1175/1520-0450(2000)039<1341:bhcaqu>2.0.co;2

[jame21128-bib-0506] Strapp, J. W. , Lilie, L. E. , Ratvasky, T. P. , Davison, C. R. , & Dumont, C. (2016). Isokinetic TWC evaporator probe: Development of the IKP2 and performance testing for the HAIC‐HIWC Darwin 2014 and Cayenne field campaigns In 8th AIAA Atmospheric and Space Environments Conference, Reston, Virginia (pp. 1–28). Reston, Virginia: American Institute of Aeronautics and Astronautics.

[jame21128-bib-0507] Straub, W. , Beheng, K. , Seifert, A. , Schlottke, J. , & Weigand, B. (2010). Numerical investigation of collision‐induced breakup of raindrops. Part II: Parameterizations of coalescence efficiencies and fragment size distributions. Journal of the Atmospheric Sciences, 67, 576–588. 10.1175/2009jas3175.1

[jame21128-bib-0508] Sulia, K. J. , Morrison, H. , & Harrington, J. Y. (2014). Dynamical and microphysical evolution during mixed‐phase cloud glaciation simulated using the bulk adaptive habit prediction model. Journal of the Atmospheric Sciences, 71, 4158–4180. 10.1175/jas-d-14-0070.1

[jame21128-bib-0509] Szyrmer, W. , Laroche, S. , & Zawadzki, I. (2005). A microphysical bulk formulation based on scaling normalization of the particle size distribution. Part I: Description. Journal of the Atmospheric Sciences, 62(12), 4206–4221. 10.1175/jas3620.1

[jame21128-bib-0514] Takahashi, T. (1993). High ice crystal production in winter cumuli over the Japan Sea. Geophysical Research Letters, 20(6), 451–454. 10.1029/93GL00613

[jame21128-bib-0515] Takahashi, T. , Nagao, Y. , & Kushiyama, Y. (1995). Possible high ice particle production during graupel‐graupel collisions. Journal of the Atmospheric Sciences, 52(24), 4523–4527. 10.1175/1520-0469(1995)052<4523:phippd>2.0.co;2

[jame21128-bib-1001] Telford, J. W. , & Chai, S. K. (1980). A new aspect of condensation theory. Pure and Applied Geophysics, 118, 720–742.

[jame21128-bib-0516] Testik, F. Y. (2009). Outcome regimes of binary raindrop collisions. Atmospheric Research, 94, 389–399. 10.1016/j.atmosres.2009.06.017

[jame21128-bib-0517] Testik, F. Y. , Barros, A. P. , & Bliven, L. F. (2006). Field observations of multimode raindrop oscillations by high‐speed imaging. Journal of the Atmospheric Sciences, 63, 2663–2668. 10.1175/jas3773.1

[jame21128-bib-0518] Testik, F. Y. , Barros, A. P. , & Bliven, L. F. (2011). Toward a physical characterization of raindrop collision outcomes. Journal of the Atmospheric Sciences, 68, 1097–1113. 10.1175/2010jas3706.1

[jame21128-bib-0519] Thayer‐Calder, K. , Gettelman, A. , Craig, C. , Goldhaber, S. , Bogenschutz, P. A. , Chen, C.‐C. , Morrison, H. , Höft, J. , Raut, E. , Griffin, B. M. , Weber, J. K. , Larson, V. E. , Wyant, M. C. , Wang, M. , Guo, Z. , & Ghan, S. J. (2015). A unified parameterization of clouds and turbulence using CLUBB and subcolumns in the community atmosphere model. Geoscientific Model Development, 8, 3801–3821. 10.5194/gmd-8-3801-2015

[jame21128-bib-0520] Thiemann, M. , Trosset, M. , & Gupta, H. (2001). Bayesian recursive parameter estimation for hydrology. Water Resources Research, 37, 2521–2535. 10.1029/2000WR900405

[jame21128-bib-0521] Thomas, S. , Ovchinnikov, M. , Yang, F. , Van der Voort, D. , Cantrell, W. , Krueger, S. , & Shaw, R. A. (2019). Scaling of an atmospheric model to simulate turbulence and microphysics in the Pi chamber. Journal of Advances in Modeling Earth Systems, 11, 1981–1994. 10.1029/2019MS001670

[jame21128-bib-0522] Thompson, G. , & Eidhammer, T. (2014). A study of aerosol impacts on clouds and precipitation development in a large winter cyclone. Journal of the Atmospheric Sciences, 71, 3636–3658. 10.1175/jas-d-13-0305.1

[jame21128-bib-0523] Thurai, M. , Petersen, W. A. , Tokay, A. , Schultz, C. , & Gatlin, P. (2011). Drop size distribution comparisons between Parsivel and 2‐D video disdrometers. Advances in Geosciences, 30, 3–9. 10.5194/adgeo-30-3-2011

[jame21128-bib-0524] Titterington, D. M. (2004). Bayesian methods for neural networks and related models. Statistical Science 2004, 19(1), 128–139. 10.1214/088342304000000099

[jame21128-bib-0525] Tobo, Y. , DeMott, P. J. , Hill, T. C. J. , Prenni, A. J. , Swoboda‐Colberg, N. G. , Franc, G. D. , & Kreidenweis, S. M. (2014). Organic matter matters for ice nuclei of agricultural soil origin. Atmospheric Chemistry and Physics, 14, 8521–8531. 10.5194/acp-14-8521-2014

[jame21128-bib-0526] Todd, C. J. (1964). A system for computing ice phase hydrometeor development. Meteorology Research Inc., Rept. ARG‐64 Pa‐121, 30 pp.

[jame21128-bib-0527] Tokay, A. , Kruger, A. , & Krajewski, W. F. (2001). Comparison of drop size distribution measurements by impact and optical disdrometers. Journal of Applied Meteorology, 40, 2083–2097. 10.1175/1520-0450(2001)040,2083:CODSDM.2.0.CO;2

[jame21128-bib-0528] Tridon, F. , & Battaglia, A. (2015). Dual‐frequency radar Doppler spectral retrieval of rain drop size distributions and entangled dynamic variables. Journal of Geophysical Research, 120, 5585–5601. 10.1002/2014JD023023

[jame21128-bib-0529] Tridon, F. , Battaglia, A. , Luke, E. , & Kollias, P. (2017). Rain retrieval from dual‐frequency radar Doppler spectra: Validation and potential for a midlatitude precipitating case‐study. Quarterly Journal of the Royal Meteorological Society, 143, 1364–1380. 10.1002/qj.3010

[jame21128-bib-0530] Twomey, S. (1964). Computations of rain formation by coalescence. Journal of the Atmospheric Sciences, 23, 405–411. 10.1175/1520-0469(1966)023<0405:corfbc>2.0.co;2

[jame21128-bib-0531] Tzivion, S. , Feingold, G. , & Levin, Z. (1987). An efficient numerical solution to the stochastic collection equation. Journal of the Atmospheric Sciences, 44, 3139–3149. 10.1175/1520-0469(1987)044<3139:aenstt>2.0.co;2

[jame21128-bib-0532] Tzivion, S. , Feingold, G. , & Levin, Z. (1989). The evolution of raindrop spectra. Part II: Collisional collection/breakup and evaporation in a rainshaft. Journal of the Atmospheric Sciences, 46, 3312–3328. 10.1175/1520-0469(1989)046<3312:teorsp>2.0.co;2

[jame21128-bib-0533] Ullrich, R. , Hoose, C. , Möhler, O. , Niemand, M. , Wagner, R. , Höhler, K. , Hiranuma, N. , Saathoff, H. , & Leisner, T. (2017). A new ice nucleation active site parameterization for desert dust and soot. Journal of the Atmospheric Sciences, 74(3), 699–717. 10.1175/JAS-D-16-0074.1

[jame21128-bib-0534] Unterstrasser, S. , Hoffmann, F. , & Lerch, M. (2017). Collection/aggregation algorithms in Lagrangian cloud micro‐physical models: Rigorous evaluation in box model simulations. Geoscientific Model Development, 10, 1521–1548. 10.5194/gmd-10-1521-2017

[jame21128-bib-0535] Unterstrasser, S. , & Sölch, I. (2014). Optimisation of the simulation particle number in a Lagrangian ice microphysical model. Geoscientific Model Development, 7, 695–709. 10.5194/gmd-7-695-2014

[jame21128-bib-0536] Vaillancourt, P. A. , Yau, M. K. , Bartello, P. , & Grabowski, W. W. (2002). Microscopic approach to cloud droplet growth by condensation. Part II: Turbulence, clustering, and condensational growth. Journal of the Atmospheric Sciences, 59, 3421–3435. 10.1175/1520-0469(2002)059<3421:matcdg>2.0.co;2

[jame21128-bib-0537] Valdez, M. P. , & Young, K. C. (1985). Number fluxes in equilibrium raindrop populations: A Markov chain analysis. Journal of the Atmospheric Sciences, 42, 1024–1036. 10.1175/1520-0469(1985)042<1024:nfierp>2.0.co;2

[jame21128-bib-0538] van den Heever, S. C. , Carrió, G. G. , Cotton, W. R. , Demott, P. J. , & Prenni, A. J. (2006). Impacts of nucleating aerosol on Florida storms. Part I: Mesoscale simulations. Journal of the Atmospheric Sciences, 63(7), 1752–1775. 10.1175/jas3713.1

[jame21128-bib-0539] van den Heever, S. C. , & Cotton, W. R. (2004). The impact of hail size on simulated supercell storms. Journal of the Atmospheric Sciences, 61, 1596–1609. 10.1175/1520-0469(2004)061<1596:tiohso>2.0.co;2

[jame21128-bib-0540] Van Diedenhoven, B. (2018). Remote sensing of crystal shapes in ice clouds In Kokhanovsky A. (Ed.), Light scattering, volume 2: Light scattering, radiative transfer and remote sensing (pp. 197–250). New York City, New York: Springer International 10.1007/978-3-319-70808-9_5

[jame21128-bib-0541] Van Diedenhoven, B. , Ackerman, A. S. , Fridlind, A. M. , Cairns, B. , & Riedi, J. (2020). Global statistics of cloud top ice microphysical and optical properties. Journal of Geophysical Research: Atmospheres, 125, e2019JD031811 10.1029/2019JD031811

[jame21128-bib-0542] Van Diedenhoven, B. , Cairns, B. , Geogdzhayev, I. V. , Fridlind, A. M. , Ackerman, A. S. , Yang, P. , & Baum, B. A. (2012). Remote sensing of ice crystal asymmetry parameter using multi‐directional polarization measurements. Part I: Methodology and evaluation with simulated measurements. Atmospheric Measurement Techniques, 5, 2361–2374. 10.5194/amt-5-2361-2012

[jame21128-bib-0543] Van Diedenhoven, B. , Fridlind, A. M. , Ackerman, A. S. , & Cairns, B. (2012). Evaluation of hydrometeor phase and ice properties in cloud‐resolving model simulations of tropical deep convection using radiance and polarization measurements. Journal of the Atmospheric Sciences, 69, 3290–3314. 10.1175/JAS-D-11-0314.1

[jame21128-bib-0544] Van Diedenhoven, B. , Fridlind, A. M. , Cairns, B. , & Ackerman, A. S. (2014). Variation of ice crystal size, shape and asymmetry parameter in tops of tropical deep convective clouds. Journal of Geophysical Research: Atmospheres, 119, 11,809–11,825. 10.1002/2014JD022385

[jame21128-bib-0545] van Lier‐Walqui, M. , Morrison, H. , Kumjian, M. R. , Reimel, K. J. , Prat, O. P. , Lunderman, S. , & Morzfeld, M. (2020). A Bayesian approach for statistical‐physical bulk parameterization of rain microphysics, Part II: Idealized Markov chain Monte Carlo experiments. Journal of the Atmospheric Sciences, 77, 1043–1064. 10.1175/JAS-D-19-0071.1

[jame21128-bib-0546] van Lier‐Walqui, M. , Vukicevic, T. , & Posselt, D. J. (2012). Quantification of cloud microphysical parameterization uncertainty using radar reflectivity. Monthly Weather Review, 140, 3442–3466. 10.1175/mwr-d-11-00216.1

[jame21128-bib-0547] van Lier‐Walqui, M. , Vukicevic, T. , & Posselt, D. J. (2014). Linearization of microphysical parameterization uncertainty using multiplicative process perturbation parameters. Monthly Weather Review, 142, 401–413. 10.1175/mwr-d-13-00076.1

[jame21128-bib-0548] van Weverberg, K. , Vogelmann, A. M. , Morrison, H. , & Milbrandt, J. (2012). Sensitivity of idealized squall‐line simulations to the level of complexity used in two‐moment bulk microphysics schemes. Monthly Weather Review, 140, 1883–1907. 10.1175/mwr-d-11-00120.1

[jame21128-bib-0549] vanZanten, M. C. , Stevens, B. , Nuijens, L. , Siebesma, A. P. , Ackerman, A. S. , Burnet, F. , Cheng, A. , Couvreux, F. , Jiang, H. , Khairoutdinov, M. , Kogan, Y. , Lewellen, D. C. , Mechem, D. , Nakamura, K. , Noda, A. , Shipway, B. J. , Slawinska, J. , Wang, S. , & Wyszogrodzki, A. (2011). Controls on precipitation and cloudiness in simulations of trade‐wind cumulus as observed during rico. Journal of Advances in Modeling Earth Systems, 3, M06001 10.1029/2011ms000056

[jame21128-bib-0550] Vardiman, L. (1978). The generation of secondary ice particles in clouds by crystal‐crystal collision. Journal of the Atmospheric Sciences, 35(11), 2168–2180. 10.1175/1520-0469(1978)035<2168:TGOSIP>2.0.CO;2

[jame21128-bib-0551] Verlinde, J. , & Cotton, W. R. (1993). Fitting microphysical observations of nonsteady convective clouds to a numerical model: An application of the adjoint technique of data assimilation to a kinematic model. Monthly Weather Review, 121(10), 2776–2793. 10.1175/1520-0493(1993)121<2776:fmoonc>2.0.co;2

[jame21128-bib-0552] Vié, B. , Pinty, J.‐P. , Berthet, S. , & Leriche, M. (2016). LIMA (v1.0): A quasi two‐moment microphysical scheme driven by a multimodal population of cloud condensation and ice freezing nuclei. Geoscientific Model Development, 9, 567–586. 10.5194/gmd-9-567-2016

[jame21128-bib-0553] Vivekanandan, J. , Zhang, G. , & Brandes, E. (2004). Polarimetric radar estimators based on a constrained gamma drop size distribution model. Journal of Applied Meteorology, 43(2), 217–230. 10.1175/1520-0450(2004)043<0217:PREBOA>2.0.CO;2

[jame21128-bib-0554] Vivekanandan, J. , Zrnic, D. S. , Ellis, S. M. , Oye, R. , Ryzhkov, A. , & Straka, J. (1999). Cloud microphysics retrieval using S‐band dual‐polarization radar measurements. Bulletin of the American Meteorological Society, 80, 381–388. 10.1175/1520-0477(1999)080<0381:cmrusb>2.0.co;2

[jame21128-bib-0555] Wagner, R. , Bunz, H. , Linke, C. , Mohler, O. , Naumann, K.‐H. , Saathhoff, H. , Schnaiter, M. , & Schurath, U. (2006). Chamber simulations of cloud chemistry: The AIDA chamber In Barnes I., & Rudzinksi K. J. (Eds.), Environmental simulation chambers: Application to atmospheric chemical processes (pp. 67–82). New York: Springer.

[jame21128-bib-0556] Wagner, R. , Kiselev, A. , Möhler, O. , Saathoff, H. , & Steinke, I. (2016). Pre‐activation of ice‐nucleating particles by the pore condensation and freezing mechanism. Atmospheric Chemistry and Physics, 16, 2025–2042. 10.5194/acp-16-2025-2016

[jame21128-bib-0557] Wakazuki, Y. , Nakamura, M. , Kanada, S. , & Muroi, C. (2008). Climatological reproducibility evaluation and future climate projection of extreme precipitation events in the baiu season using a high‐resolution non‐hydrostatic RCM in comparison with an AGCM. Journal of the Meteorological Society of Japan, 86, 951–967. 10.2151/jmsj.86.951

[jame21128-bib-0558] Wang, C. , Platnick, S. , Fauchez, T. , Meyer, K. , Zhang, Z. , Iwabuchi, H. , & Kahn, B. H. (2019). An assessment of the impacts of cloud vertical heterogeneity on global ice cloud data records from passive satellite retrievals. Journal of Geophysical Research, 124, 1578–1595. 10.1029/2018JD029681

[jame21128-bib-0559] Wang, D. , Giangrande, S. E. , Schiro, K. , Jensen, M. P. , & Houze, R. A. Jr. (2019). The characteristics of tropical and midlatitude mesoscale convective systems as revealed by radar wind profilers. Journal of Geophysical Research: Atmospheres, 124, 4601–4619. 10.1029/2018JD030087

[jame21128-bib-0560] Wang, H. , Skamarock, W. C. , & Feingold, G. (2009). Evaluation of scalar advection schemes in the advanced research WRF model using large‐eddy simulations of aerosol‐cloud interactions. Monthly Weather Review, 137, 2547–2558. 10.1175/2009mwr2820.1

[jame21128-bib-0561] Wang, L.‐P. , Rosa, B. , Gao, H. , He, G. , & Jin, G. (2009). Turbulent collision of inertial particles: Point‐particle based, hybrid simulations and beyond. International Journal of Multiphase Flow, 35, 854–867. 10.1016/j.ijmultiphaseflow.2009.02.012

[jame21128-bib-0562] Weisman, M. L. , Davis, C. , Wang, W. , Manning, K. W. , & Klemp, J. B. (2008). Experiences with 0–36‐h convective forecasts with the WRF‐ARW model. Weather and Forecasting, 23, 407–436. 10.1175/2007WAF2007005.1

[jame21128-bib-0563] Wendisch, M. , & Brenguier, J. L. (2008). Airborne measurements for environmental research (p. 655). Hoboken, New Jersey: Wiley.

[jame21128-bib-0564] Werdell, P. J. , Behrenfeld, M. J. , Bontempi, P. S. , Boss, E. , Cairns, B. , Davis, G. T. , Franz, B. A. , Gliese, U. B. , Gorman, E. T. , Hasekamp, O. , Knobelspiesse, K. D. , Mannino, A. , Martins, J. V. , McClain, C. R. , Meister, G. , & Remer, L. A. (2019). The plankton, aerosol, cloud, ocean ecosystem (PACE) mission: Status, science, advances. Bulletin of the American Meteorological Society, 100(9), 1775–1794. 10.1175/BAMS-D-18-0056.1

[jame21128-bib-0565] Westbrook, C. D. , Illingworth, A. J. , O'Connor, E. J. , & Hogan, R. J. (2010). Doppler lidar measurements of oriented planar ice crystals falling from supercooled and glaciated layer clouds. Quarterly Journal of the Royal Meteorological Society, 136, 260–276. 10.1002/qj.528

[jame21128-bib-0567] Wilkinson, M. (2016). Large deviation analysis of rapid onset of rain showers. Physical Review Letters, 116, 018501 10.1103/PhysRevLett.116.018501 26799046

[jame21128-bib-0568] Williams, C. R. (2016). Reflectivity and liquid water content vertical decomposition diagrams to diagnose vertical evolution of raindrop size distributions. Journal of Atmospheric and Oceanic Technology, 33, 579–595. 10.1175/jtech-d-15-0208.1

[jame21128-bib-0569] Wong, M. , Romine, G. , & Snyder, C. (2019). Model improvement via systematic investigation of physics tendencies. Monthly Weather Review (accepted, 148). 10.1175/mwr-d-19-0255.1

[jame21128-bib-0570] Wood, R. , Irons, S. , & Jonas, P. R. (2002). How important is the spectra ripening effect in stratiform boundary layer clouds? Studies using simple trajectory analysis. Journal of the Atmospheric Sciences, 59, 3620–3634. 10.1175/1520-0469(2002)059<2681:hiitsr>2.0.co;2

[jame21128-bib-0571] Woodcock, A. H. , Duce, R. A. , & Moyers, J. L. (1971). Salt particles and raindrops in Hawaii. Journal of the Atmospheric Sciences, 28, 1251–1257. 10.1175/1520-0469(1971)028<1252:sparih>2.0.co;2

[jame21128-bib-0572] Woods, C. P. , Stoelinga, M. T. , & Locatelli, J. D. (2007). The IMPROVE‐1 storm of 1‐2 February 2001. Part III: Sensitivity of a mesoscale model simulation to the representation of snow particle types and testing of a bulk microphysical scheme with snow habit prediction. Journal of the Atmospheric Sciences, 64, 3927–3948. 10.1175/2007jas2239.1

[jame21128-bib-0573] Woods, S. , Lawson, R. P. , Jensen, E. , Bui, T. P. , Thornberry, T. , Rollins, A. , Pfister, L. , & Avery, M. (2018). Microphysical properties of tropical tropopause layer cirrus. Journal of Geophysical Research: Atmospheres, 123, 6053–6069. 10.1029/2017JD028068 PMC6907018 31832294

[jame21128-bib-0574] Wu, W. , & McFarquhar, G. G. (2018). Statistical theory on the functional form of cloud particle size distributions. Journal of the Atmospheric Sciences, 75, 2801–2814. 10.1175/jas-d-17-0164.1

[jame21128-bib-0575] Wyszogrodzki, A. A. , Grabowski, W. W. , Wang, L.‐P. , & Ayala, O. (2013). Turbulent collision‐coalescence in maritime shallow convection. Atmospheric Chemistry and Physics, 13, 8471–8487. 10.5194/acp-13-8471-2013

[jame21128-bib-0576] Xu, Z. , Mace, G. G. , & Posselt, D. J. (2019). A method for assessing relative skill in retrieving cloud and precipitation properties in next generation cloud radar and radiometer orbiting observatories. Journal of Atmospheric and Oceanic Technology, 36, 2283–2306. 10.1175/JTECH-D-18-0204.1

[jame21128-bib-0577] Xue, L. , Fan, J. , Lebo, Z. J. , Wu, W. , Morrison, H. , Grabowski, W. W. , Chu, X. , Geresdi, I. , North, K. , Stenz, R. , & Gao, Y. (2017). Idealized simulations of a squall line from the MC3E field campaign applying three bin microphysics schemes: Dynamic and thermodynamic structure. Monthly Weather Review, 145(12), 4789–4812. 10.1175/mwr-d-16-0385.1

[jame21128-bib-0578] Yang, F. , Cruikshank, O. , He, W. , Kostinski, A. , & Shaw, R. A. (2018). Nonthermal ice nucleation observed at distorted contact lines of supercooled water drops. Physical Review E, 97, 023103 10.1103/PhysRevE.97.023103 29548219

[jame21128-bib-0579] Yano, J.‐I. , Heymsfield, A. J. , & Phillips, V. T. (2016). Size distributions of hydrometeors: Analysis with the maximum entropy principle. Journal of the Atmospheric Sciences, 73, 95–108. 10.1175/JAS-D-15-0097.1

[jame21128-bib-0580] Yano, J.‐I. , & Phillips, V. T. J. (2010). Ice‐ice collisions: An ice multiplication process in atmospheric clouds. Journal of the Atmospheric Sciences, 68(2), 322–333. 10.1175/2010JAS3607.1

[jame21128-bib-0582] Young, K. C. (1974). A numerical simulation of wintertime, orographic precipitation: Part I: Description of model microphysics and numerical techniques. Journal of the Atmospheric Sciences, 31, 1735–1748. 10.1175/1520-0469(1974)031<1735:ansowo>2.0.co;2

[jame21128-bib-0583] Zhang, C. , & Harrington, J. Y. (2014). Including surface kinetic effects in simple models of ice vapor diffusion. Journal of the Atmospheric Sciences, 71, 372–390. 10.1175/JAS-D-13-0103.1

[jame21128-bib-0584] Zhang, C. , & Harrington, J. Y. (2015). The effects of surface kinetics on crystal growth and homogeneous freezing in parcel simulations of cirrus. Journal of the Atmospheric Sciences, 72, 2929–2946. 10.1175/JAS-D-14-0285.1

[jame21128-bib-0585] Zhang, C. , Wang, M. , Morrison, H. , Somerville, R. C. J. , Zhang, K. , Liu, X. , & Li, J.‐L. F. (2014). Investigating ice nucleation in cirrus clouds with an aerosol‐enabled multiscale modeling framework. Journal of Advances in Modeling Earth Systems, 6, 998–1015. 10.1002/2014MS000343

[jame21128-bib-0586] Zhang, G. , Sun, J. , & Brandes, E. A. (2006). Improving parameterization of rain microphysics with disdrometer and radar observations. Journal of the Atmospheric Sciences, 63, 1273–1290. 10.1175/JAS3680.1

[jame21128-bib-0587] Zhang, J. , Lohmann, U. , & Lin, B. (2002). A new statistically based autoconversion rate parameterization for use in large‐scale models. Journal of Geophysical Research, 107(D24), 4750 10.1029/2001JD001484

[jame21128-bib-0588] Zhang, X. , & Zheng, G. (1994). A simple droplet spectrum derived from entropy theory. Atmospheric Research, 32, 189–193. 10.1016/0169-8095(94)90059-0

[jame21128-bib-0589] Ziegler, C. L. (1985). Retrieval of thermal and microphysical variables in observed convective storms. Part 1: Model development and preliminary testing. Journal of the Atmospheric Sciences, 42, 1487–1509. 10.1175/1520-0469(1985)042<1487:rotamv>2.0.co;2

[jame21128-bib-0590] Zrnic, D. S. , Kimpel, J. F. , Forsyth, D. E. , Shapiro, A. , Crain, G. , Ferek, R. , Heimmer, J. , Benner, W. , McNellis, F. T. J. , & Vogt, R. J. (2007). Agile‐beam phased array radar for weather observations. Bulletin of the American Meteorological Society, 88, 1753–1766. 10.1175/bams-88-11-1753

[jame21128-bib-0591] Zrnic, D. S. , & Ryzhkov, A. V. (1999). Polarimetry for weather surveillance radars. Bulletin of the American Meteorological Society, 80, 389–406. 10.1175/1520-0477(1999)080<0389:pfwsr>2.0.co;2

